# Collagen Sequence Analysis Reveals Evolutionary History of Extinct West Indies *Nesophontes* (Island-Shrews)

**DOI:** 10.1093/molbev/msaa137

**Published:** 2020-06-04

**Authors:** Michael Buckley, Virginia L Harvey, Johanset Orihuela, Alexis M Mychajliw, Joseph N Keating, Juan N Almonte Milan, Craig Lawless, Andrew T Chamberlain, Victoria M Egerton, Phillip L Manning

**Affiliations:** m1 Interdisciplinary Centre for Ancient Life, School of Natural Sciences, University of Manchester, Manchester, United Kingdom; m2 Manchester Institute of Biotechnology, University of Manchester, Manchester, United Kingdom; m3 Department of Earth and Environment, Florida International University, Miami, FL; m4 La Brea Tar Pits & Museum, Natural History Museum of Los Angeles County, Los Angeles, CA; m5 School of Earth Sciences, University of Bristol, Life Sciences Building, Bristol, United Kingdom; m6 Museo Nacional de Historia Natural “Prof. Eugenio de Jesús Marcano”, Santo Domingo, Dominican Republic; m7 School of Biological Sciences, Faculty of Biology, Medicine and Health, University of Manchester, Manchester, United Kingdom; m8 The Children’s Museum of Indianapolis, Natural Sciences, Indianapolis, IN

**Keywords:** *Nesophontes*, collagen fingerprinting, paleoproteomics, biogeography, phylogenetics

## Abstract

Ancient biomolecule analyses are proving increasingly useful in the study of evolutionary patterns, including extinct organisms. Proteomic sequencing techniques complement genomic approaches, having the potential to examine lineages further back in time than achievable using ancient DNA, given the less stringent preservation requirements. In this study, we demonstrate the ability to use collagen sequence analyses via proteomics to assist species delimitation as a foundation for informing evolutionary patterns. We uncover biogeographic information of an enigmatic and recently extinct lineage of *Nesophontes* across their range on the Caribbean islands. First, evolutionary relationships reconstructed from collagen sequences reaffirm the affinity of *Nesophontes* and *Solenodon* as sister taxa within Solenodonota. This relationship helps lay the foundation for testing geographical isolation hypotheses across islands within the Greater Antilles, including movement from Cuba toward Hispaniola. Second, our results are consistent with Cuba having just two species of *Nesophontes* (*N. micrus* and *N. major*) that exhibit intrapopulation morphological variation. Finally, analysis of the recently described species from the Cayman Islands (*N. hemicingulus*) indicates that it is a closer relative to *N. major* rather than *N. micrus* as previously speculated. This proteomic sequencing improves our understanding of the origin, evolution, and distribution of this extinct mammal lineage, particularly with respect to the approximate timing of speciation. Such knowledge is vital for this biodiversity hotspot, where the magnitude of recent extinctions may obscure true estimates of species richness in the past.

## Introduction

The islands of the Caribbean have experienced some of the largest postglacial mammal losses known (Turvey and Fritz 2011). These anthropogenic impacts—including deforestation and predation by invasive species—continue to devastate the surviving fauna (Cooke et al. 2017). Today, 8 of the 12 (described) surviving nonvolant mammals are listed in an IUCN Red List threat category (Vulnerable, Endangered, or Critically Endangered), with all remaining species classified as Near Threatened (Turvey et al. 2017). The rate of current endangerment, as well as the magnitude of past extinctions, are conservative estimates. However, the existence of extinct species, including potentially hitherto unknown species, undermines our ability to accurately assess biodiversity before human interference in this biogeographic system (Mace 2004; Bickford et al. 2007). Including bats, nearly 90% of all Caribbean native land mammals have gone extinct since the late Pleistocene/early Holocene (Cooke et al. 2017).

Holocene extinctions have hindered biogeographic inferences in such systems because species delimitation based on the fossil record alone has its limitations (e.g., the subjectivity of morphological criteria limited to skeletal remains, misinterpretations of sexual dimorphism as distinct species, taphonomic bias, and ontogenetic variation [allometry]). The islands of the Caribbean are widely considered to be important evolutionary laboratories for studying overwater dispersal, vicariance, and in situ diversification that generates species diversity and underlies colonization–extinction dynamics (Ricklefs and Bermingham 2008). The region consists of three distinctive island groups: the Bahamas (low-lying islands on carbonate platforms), the Greater Antilles (large, old fragments of continental crust, volcanic intrusions, plutonic, and mélange units), and the Lesser Antilles (situated on a volcanic arc; Donnelly 1989; Draper and Barros 1994). As a result, different island groups have different proportions of endemic and continental faunas linked to their historical proximity to North, Central, and South America, and historical biogeography has been the focus of significant debate using genetic and paleontological data.

The center of mammalian endemism within the Caribbean is the Greater Antilles (Jamaica, Puerto Rico, Hispaniola, Cuba, and the Cayman Islands), with in situ radiations often attributed to the region’s complex geotectonic history and impacts of Plio-Pleistocene sea-level fluctuations (Davalos 2004). These unique faunas include multiple species of monkeys (Pitheciidae), sloths (Megalonychidae), hutias (Capromyidae), spiny rats (Echimyidae), “giant hutias” (Heptaxodontidae), and the morphologically shrew-like insectivores solenodons (*Solenodon*) (note some authors recognize the two extant species as separate genera, the other being *Atopogale;* Solenodontidae) and island-shrews (*Nesophontes*; Nesophontidae), the large majority of which are now extinct (Brace et al. 2016). Most genetic biogeographic studies in the Greater Antilles have focused on invertebrates (Svenson and Rodrigues 2017) and reptiles (Kemp and Hadly 2015; Tucker et al. 2017), as the severely reduced diversity of extant mammals coupled with the high thermal age of the tropics has limited direct phylogenetic inferences using molecular approaches (Kemp and Hadly 2016).

Recent advances in the amplification of DNA from degraded material opened several windows into the origin of Caribbean mammalian diversity. Insight into the exceptionally rare, giant (∼1 kg) insectivore *Solenodon* using museum collections and degraded modern tissue samples (Grigorev et al. 2018), strongly suggest a Mesozoic, North American origin for the family. Ancient DNA from a ∼750-year-old subfossil of *Nesophontes* represented the first paleogenetic material from the Greater Antilles and was able to confirm longstanding morphological hypotheses uniting Nesophontidae with Solenodontidae as a single lineage the Solenodonota having diverged ∼70 Ma from all other living true insectivores (Eulipotyphla: shrews, hedgehogs, moles) (Roca et al. 2004; Brace et al. 2016). Shared-derived characters that have been proposed for this lineage include modification of the buccal styles on the upper molars, large and funnel-like lacrimal foramen on the orbital margin, and the position of the origin of the *levator labii superioris proprius* (McDowell 1958). It is suggested that *Solenodon* and *Nesophontes* likely diverged from each other ∼57 Ma (between 44 and 69 Ma; Brace et al. 2016).

This common ancestry now lays the foundation for understanding evolutionary trends in this group, including contrasting paleobiogeographic patterns, diversification rates, and ecological strategies to island life. The “island-shrews” of the Nesophontidae were found across all of the Greater Antillean islands except Jamaica and ranged in body size from ∼10–150 g (Turvey et al. 2007). Their extinction ∼500 years ago resulted in the loss of at least 44 My of unique evolutionary history, and likely more given that this estimate was based on only one of these species (Turvey and Fritz 2011).

### *Nesophontes*—A Historical Background

The enigmatic Caribbean “island-shrews” or nesophontids are represented by the single genus *Nesophonte*s and are known only from subfossils found in surficial cave deposits and raptor roosts (Anthony 1916; Miller 1929). Brain endocasts suggest that *Nesophontes* was terrestrial and likely a nocturnal semiburrower (fossorial) with a great sensibility to tactile and olfactory stimuli, similar to living true shrews (Eulipotyphla: Soricidae; Orihuela 2014). *Nesophontes* may have been venomous like the extant *Solenodon* (Turvey 2010) as well as some modern shrews (Ligabue-Braun et al. 2012). Nesophontid remains have been found at a range of altitudes and ecosystems, from coastal plains to humid forest and montane environments, indicating that they tolerated broad ecological conditions. The swift extinction of the different *Nesophontes* species, as well as several of the smallest endemic rodents (e.g., *Brotomys*) across the Greater Antilles, likely resulted from the introduction of rats (*Rattus* spp.), cats, and mongooses after 1492 AD (Diaz-Franco 2004; Cooke et al. 2017).

The lack of *Nesophontes* molecular data has led to numerous conflicting morphology-based taxonomies, with overall size often used to differentiate species on different islands (Miller 1929; Arredondo 1970; McFarlane 1999; Orihuela 2014). Sexual dimorphism has been proposed as a potential cause of the large size variation observed in the genus ever since the first description of *Nesophontes* (Anthony 1916) and most species thereon (McFarlane 1999; Condis Fernández et al. 2005; Silva et al. 2007; Rzebik-Kowalska and Wołoszyn 2012), with putative females (e.g., for *Nesophontes major* and *N. micrus* from Cuba; or *N. edithae* from Puerto Rico) generally smaller and exhibiting less pronunciation of mandibular muscle scars than the males (McFarlane 1999; Silva et al. 2007). Documenting sexual size dimorphism (SSD) in the fossil record can be challenging (Plavcan 1994), and it is noteworthy that modern eulipotyphlans are not known to exhibit SSD (Lindenfors et al. 2007). It has also been suggested that allochronic size variation could be another explanation for the resultant species delimitations (McFarlane 1999), although this would be more appropriately investigated via the inclusion of various dating methods (Harvey et al. 2016).

Depending on the authority, as few as five and as many as ten *Nesophontes* species are currently recognized as being valid: a single species from Puerto Rico (*N. edithae*; Anthony 1916; McFarlane 1999), one (*N. micrus*Allen 1917; Silva et al. 2007), two (*N. major*Arredondo 1970 and *N. micrus*Allen 1917; Condis Fernández et al. 2005), or as many as five species from Cuba (additionally including *N. longirostris*Anthony 1918, 1919), *N. superstes* (Fischer 1977) and *N. submicrus* (Arredondo 1970) depending on the author (Silva et al. 2007), and three species from Hispaniola (*N. paramicrus*, *N. hypomicrus*, and *N. zamicrus*; Miller 1929), along with one recently described species recorded from the Cayman Islands (*N. hemicingulus*, Morgan et al. 2019), present on both Cayman Brac and Grand Cayman.The Cayman Island species has been considered as being derived from one of the Cuban species, but “probably *N. micrus*, based on several shared dental features” (Morgan 1994). *Nesophontes* has also been reported to occur rarely in archaeological sites across Hispaniola and Puerto Rico, with suggestion of the Puerto Rican *N. edithae* found on the Virgin Islands as a potentially human-mediated translocation (Wing and Wing 1998), although natural dispersal may be likely given the connection of these islands with Puerto Rico at least as recently as the Late Pleistocene (Giovas 2019).

In this study, we employed a combination of collagen fingerprinting and in-depth sequencing to assess the multiple competing taxonomic and evolutionary relationship hypotheses regarding the Nesophontidae. The primary hypothesis related to the notion that multiple species are conspecific, particularly the Cuban taxa. The secondary hypothesis was that the Cayman *Nesophontes* relates to one of these. Ultimately, our aim was to use a molecular phylogeny to investigate potential dispersal events between islands and whether their distribution was shaped by more ancient geotectonic processes or was a result of Plio-Pleistocene sea-level fluctuations.

### Molecular Sequence Analysis Using Proteomics

Although it has great potential in terms of information available, DNA sequencing is a difficult approach for obtaining molecular sequence information of specimens from the tropics due to the effects of temperature and humidity on DNA degradation (Smith et al. 2001; Mulligan 2006; Gutiérrez-García et al. 2014). In our current study, we emphasize an alternative method to acquiring molecular sequence information from the dominant structural protein collagen, which has been shown to yield promising phylogenetic results (Buckley 2013, 2015; Buckley et al. 2015). The advantages of protein over aDNA analysis are as follows: 1) since no amplification takes place the contamination problems from foreign collagen are greatly reduced, and 2) peptide mass fingerprinting of bone has been demonstrated to be successful in samples dating back to at least the Pliocene (∼3.5 Ma; Rybczynski et al. 2013). Previous analyses of collagen peptide mass fingerprints (PMFs) of modern and subfossil material demonstrate that this protein is variable enough for identification to the genus level in most mammals (Buckley et al. 2017) and the species level in some (Buckley et al. 2014). Here, we apply proteomic methods to evaluate the phylogeny of various taxa of *Nesophontes*, exploring how collagen variation could be used to improve our understanding of their taxonomy, potential sexual size differences, and biogeographic history while more broadly inferring past mammalian radiations across the Greater Antilles.

## Results

### Molecular Sequence Analysis of Solenodonota

**Fig. 1.**
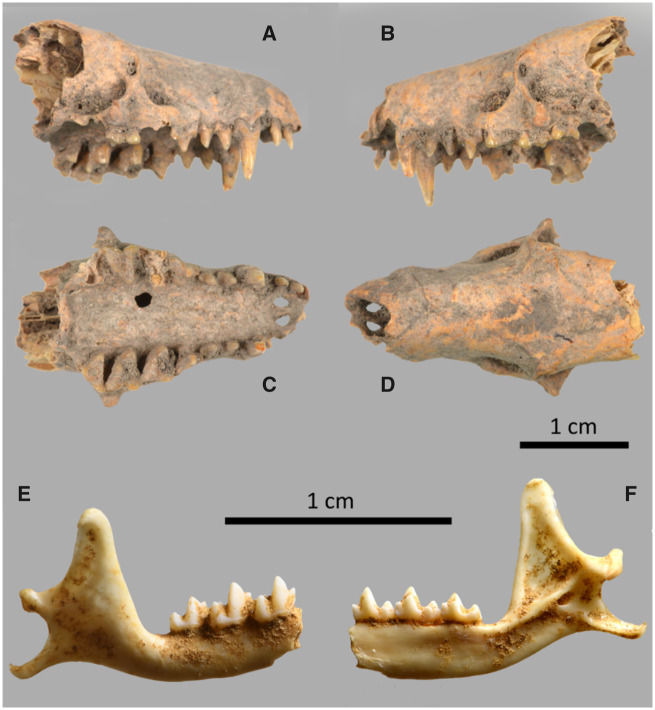
*Nesophontes edithae* skull from St. Thomas, US Virgin Islands (*A–D*) and partial left hemi-mandible of *N. hemicingulus* collected from Patton’s Fissure (*E* and *F*), Cayman Brac (scale 1 cm).

We analyzed 17 *Nesophontes* and 10 *Solenodon* specimens by PMFs and in-depth collagen sequencing for phylogenetic analyses (supplementary table S1, Supplementary Material online). Analysis also focused on two specimens of *N. micrus* and *N. major*, including samples considered to belong to each of the supposed sexes for each pair based on SSD criteria previously described by Anthony (1916) and McFarlane (1999). This assumption was also in agreement with the taxonomic arrangement proposed by Condis Fernández et al. (2005), in which *N. submicrus* is synonymized with and suspected as the female morph of *N. micrus*, and *N. superstes* suspected as the male morph of *N. major* (supplementary table S2, Supplementary Material online). The specimens analyzed here were excavated from Cueva de la Caja, Mayabeque Province (also known as “Cueva de los Nesofontes”; dated to between 1,290±30 BP and 1,418±20 BP; see Orihuela, Pérez Orozco, et al. 2020) and Cueva del Gato Jíbaro, Matanzas Province (dated to 860±30 BP; Orihuela, Viñola, et al. 2020), northwestern Cuba with permission from the Comisión Nacional de Monumentos, and the Registro Nacional de Bienes Culturales, Cuba. A scapula specimen of *Solenodon cubanus* was also recovered from a superficial layer of Cueva de la Caja and dated to 650±15 BP (Orihuela, Viñola, et al. 2020). A *Nesophontes* mandible (fig. 1*e* and *f*) was collected from Patton’s Fissure (Cayman Brac) with permission from the National Trust and the Department of Environment for the Cayman Islands (unknown age) considered as *N. hemicingulus*. From Hispaniola, we analyzed *N. hypomicrus* from Cueva de Mono (MNHNSD FOS 25.406) and *N. paramicrus* from Cayacoa, both in the Dominican Republic (MNHNSD FOS 25.422) as well as *N. zamicrus* (UF 74911) and *Solenodon paradoxus* from Haiti (UF128167/UF134729/UF134734); direct radiocarbon dates are unavailable from these sites, but they are assumed as Late Quaternary based on the contemporary mammal faunal remains in the deposits, including extinct sloths, and previous studies on Hispaniolan mammal cave assemblages (Cooke et al. 2017). From Puerto Rico, specimens of *N. edithae* were analyzed from Cueva Matos in Arecibo, a site that spans the Holocene-Pleistocene as well as a sample from St. Thomas, U.S. Virgin Islands (O.1.952, Unit 1, Square VII, Magens Bay), a site featuring the remains of a village midden, thought to have been inhabited ∼AD 800–1500 (fig. 1*a*–*d*; Wing and Wing 1998).

**Fig. 2.**
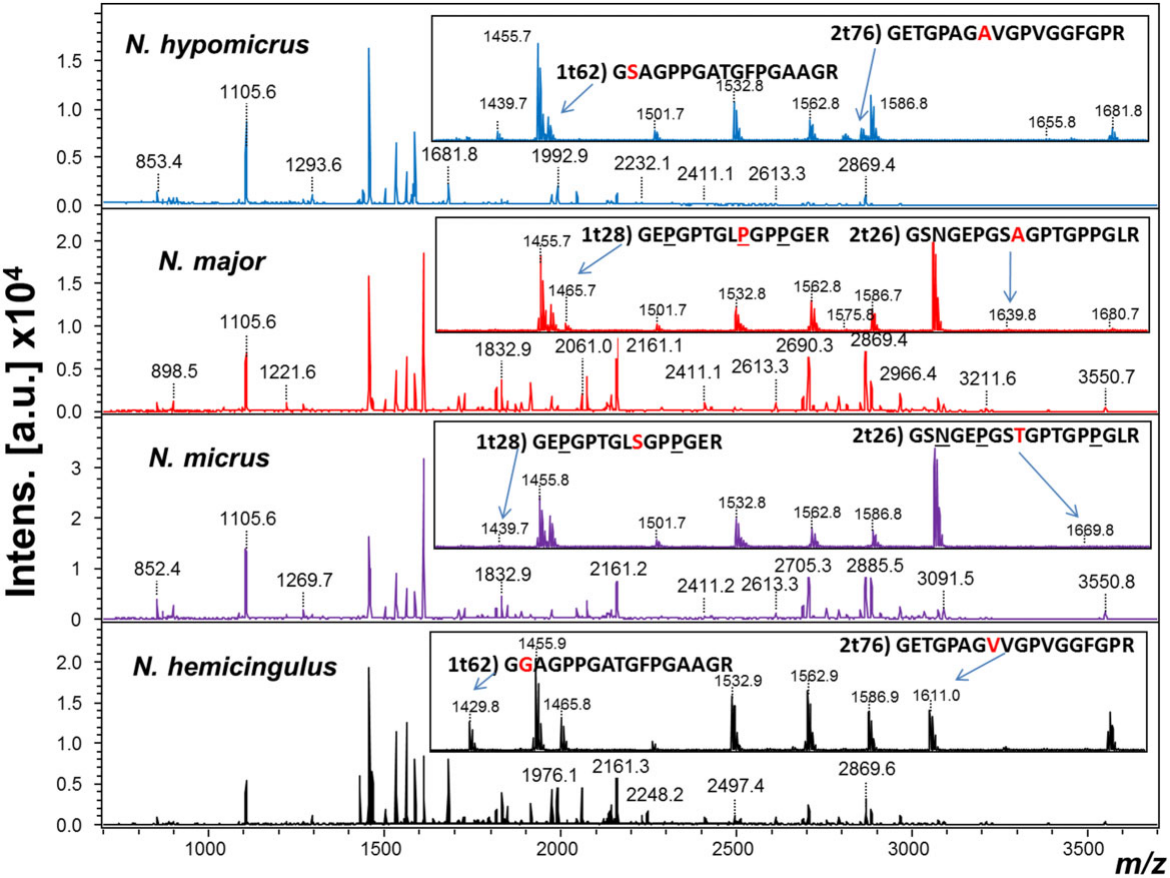
Collagen peptide mass fingerprints for the two Cuban *Nesophontes* species in comparison to the one from Cayman Brac (*N. hemicingulus*) and *N. hypomicrus* from Hispaniola with zoom-in showing some key peptide differences (insets), where red lettering indicates substituted amino acids that vary within the nesophontids. The collagen peptide mass fingerprints of the remaining taxa are shown in supplementary figures S1–S3, Supplementary Material online.

Comparison between PMFs for the seven species sampled (including the two dimorphs of *N. major* and *N. micrus*) identified more peptide peak variations and subsequently confirmed sequence differences than expected for the species level. For example, *N. micrus* exhibits at least three substitutions from *N. major* and four from *N. hemicingulus*, whereas the latter has at least eight substitutions from *N. paramicrus* (supplementary table S3, Supplementary Material online). In contrast, there are five differences observed between the two extant solenodons that are considered by some as separate genera (Casewell et al. 2019). However, these differences derive from a substantially larger number of substitution sites, with 12 sites of variation in the COL1A1 chain and a further five sites in the COL1A2 chain. Nevertheless, we found no discernible sequence differences between the potential sexual dimorphs analyzed for each species (supplementary fig. S1, Supplementary Material online) or between *N. hypomicrus* and *N. zamicrus* from Hispaniola (fig. 2). This supports the specific validity of *N. major* and *N. micrus*, indicating that the size and gracility differences observed within each taxon are likely referable to intraspecific sexual dimorphism, or other form of interspecific variation (allochronic variation is less likely with <200 years difference between these specimens), a phenomenon that likely extends to some of the Hispaniolan taxa also.

Although there were no differences observed between any replicates of the same species, there are at least 14 positions that appear to alter from *N. micrus* to other nesophontids, although there are typically up to nine amino acid variants between any two species (table 1). We compared the number of amino acid variations within these nesophontids with those of other taxa at the genus and species level (supplementary table S3, Supplementary Material online), particularly the bats as one of the only taxa within Laurasiatheria with unique sequences for more than one species of the same genus (*Myotis*). Species differences within the diverse genus *Myotis* ranged from four to ten, but this higher amount is also typical for genus-level differences (e.g., *Eptesicus fuscus* having ten amino acid differences from *Myotis lucifigus*; supplementary table S3, Supplementary Material online). Therefore, given the number of amino acid substitutions, it is entirely plausible that the nesophontid species in this study represent multiple genera by comparison to similar numbers of differences observed for the more complete sequences of known distinct genera.

**Table 1.** Number of COL1A1 and COL1A2 Amino Acid Substitutions Observed between Nesophontid and Solenodontid Taxa.

	*Solenodon cubanus*	*Solenodon paradoxus*	*Nesophontes major*	*Nesophontes hemicingulus*	*Nesophontes micrus*	*Nesophontes zamicrus*	*Nesophontes hypomicrus*	*Nesophontes paramicrus*	*Nesophontes edithae*
*Solenodon cubanus*	X	4	25	25	28	26	26	25	26
*Solenodon paradoxus*	1	X	24	24	25	25	25	24	25
*Nesophontes major*	4	5	X	1	2	1	1	3	2
*Nesophontes hemicingulus*	5	6	0	X	1	1	1	3	2
*Nesophontes micrus*	4	5	2	3	X	2	2	4	2
*Nesophontes zamicrus*	4	5	2	3	2	X	0	2	1
*Nesophontes hypomicrus*	4	5	2	3	2	0	X	2	1
*Nesophontes paramicrus*	6	7	4	6	4	3	3	X	3
*Nesophontes edithae*	7	8	3	4	3	3	3	5	X

Note.—See supplementary table S2, Supplementary Material online, for a summary of peptide differences and supplementary table S3, Supplementary Material online, for full sequences; note that, it was not possible to retrieve >16% collagen sequence from the recently published *Solenodon paradoxus* genomes (Grigorev et al. 2018; Casewell et al. 2019).

Several peptides can distinguish between *N. major* and *N. micrus* (e.g., GEPGPTGLp/sGPPGER, PGEVGPPGPPGPa/tGEK, GSNGEPGSa/tGPTGPPGLR, and GSNGEPGSt/aGPTGPPGLR; substituted amino acids indicated in lower case) and of those that do the specimen from Cayman Brac shares all with *N. major* except for one homologous unique peptide (supplementary table S4, Supplementary Material online). This is reflected in the fingerprint at *m/z* 1,429.7 with the peptide sequence GgAGPPGATGFPGAAGR in *N. hemicingulus*, which is GsAGPPGATGFPGAAGR in most mammals except for some rodents (Buckley et al. 2016), rabbit (*Oryctolagus*), and hedgehog (*Erinaceus*). There are also some peptide sequences unique to the three Hispaniolan *Nesophontes* (e.g., GVPGPPGAIGPAGK, but also in *Solenodon*) and several that appear specific to *N. paramicrus* (GVQGPPGPGGPR, VGPPGPSGgAGpPGPPGPVGK, GIPGPVGAAGASGPR, and GEAGSSGPAGPAGPR) or *N. edithae* (GFPGADGAAGPK, but this is also present in *Sorex*, GESGPSGPGGPTGAR, GETGPAGPPGAPGTPGAPGPVGPAGK, and GSNGEPGSSGPTGPPGLR). Intriguingly, the lack of confidently identified amino acid differences between *N. zamicrus* and *N. hypomicrus* indicates that, at least on molecular grounds, we cannot rule out these two Hispaniolan taxa as being conspecific either, whether due to sexual dimorphism or allochronic variation.

**Fig. 3.**
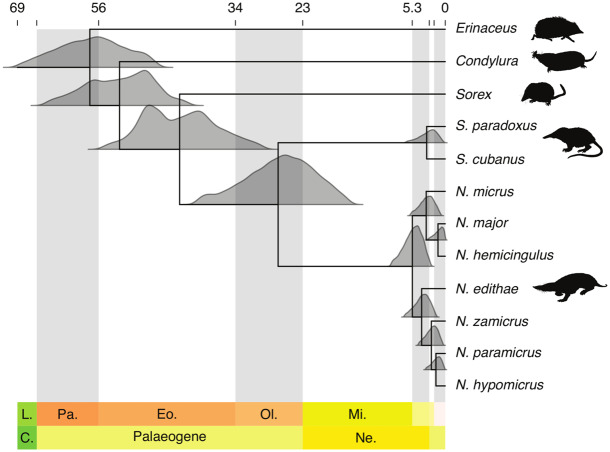
Results of our MCMCtree clock analysis showing divergence estimates within the Eulipotyphla (right). Density plots represent the posterior distribution of age estimates at each node. Silhouettes of *Solenodon* and *Sorex* from PhyloPic, reproduced under Public Domain Dedication 1.0 license. Silhouette of *Erinaceus* by Roberto Díaz Sibaja, reproduced under Creative Commons Attribution 3.0 license (https://creativecommons.org/licenses/by/3.0/). Silhouette of *Nesophontes* modified from original image by Jennifer García, reproduced under Creative Commons Attribution 3.0 license.

In wider consideration of the appropriateness of using collagen sequence analysis for phylogenetic analysis, our unconstrained analysis (supplementary fig. S6, Supplementary Material online) yielded a similar topology to that of a consensus phylogeny published for mammals based on DNA analysis (Tarver et al. 2016). However, where previous molecular phylogenetic analyses using nuclear and mitochondrial DNA consistently place Solenodontidae as the sister clade to all other living eulipotyphlan families, with moles diverging subsequently leaving shrews and hedgehogs as each other’s closest relatives (Roca et al. 2004; Meredith et al. 2011; Foley et al. 2016; Springer et al. 2018) our results place Solenodonota sister to Soricidae (fig. 3 and supplementary fig. S4, Supplementary Material online); we additionally reanalyzed the data with an additional *Sorex* + *Erinaceus* topological constraint (supplementary fig. S5, Supplementary Material online). Although these show paraphyly of a known monophyletic clade, shrews + hedgehogs, it should be noted that the molecular phylogeny could be affected by several factors: 1) as the proteomic data are based upon probability-based sequence matches of tandem spectra from collagen peptides, it contains variable amounts of missing sequence data; 2) the quality of the extant member sequences themselves (e.g., of the three extant eulipotyphlans), only the hedgehog has COL1A1 and COL1A2 sequences available in UniProt, along with partial sequences in the Ensembl genome browser, whereas neither the shrew nor the mole have entries in UniProt, and the latter with no entries in Ensembl either (shrew having only a COL1A2 sequence available) leading to a reliance on the protein BLAST search results that are not curated sequences; and 3) most importantly the effects on topology when entire clades are based on the probability-based matching of peptide sequences (i.e., perhaps in some cases the true peptide sequence is distinct from the extant sequences, but apparently identical across all nesophontids).

We find strong support for the monophyly of Solenodonota and *Nesophontes* under both the *Sorex* unconstrained and the *Sorex* constrained analyses (posterior probabilities equal 1). Within *Nesophontes*, relationships are less certain. Both analyses recover a *N. hypomicrus*–*paramicrus*–*zamicrus* clade (posterior probability equals 0.71 unconstrained, 0.85 constrained) with *N. edithae* as its immediate sister group (posterior probability equals 0.62 unconstrained, 0.66 constrained). Furthermore, both analyses resolve *N. major* and *N. hemicingulus* as sister groups, albeit with low probability (posterior probability equals 0.61 unconstrained, 0.44 constrained). The position of *N. micrus* differs between analyses. If *Sorex* is unconstrained, *N. micrus* forms a clade with *N. major* and *N. hemicingulus* (posterior probability 0.54). If *Sorex* is constrained, *N. micrus* is resolved as the sister of the *N. hypomicrus*–*paramicrus*–*zamicrus–edithae* clade (posterior probability equals 0.65). Our MCMCtree analysis places the 95% highest posterior density for the *Nesophontes* divergence between 2.71 and 9.35 Ma. The solenodonota divergence is less precisely dated at between 16.05 and 40.71 Ma. However, these topological differences have little impact on our age estimates (see supplementary table S5, Supplementary Material online).

### Morphological Measurements and Sexual Dimorphism

**Fig. 4.**
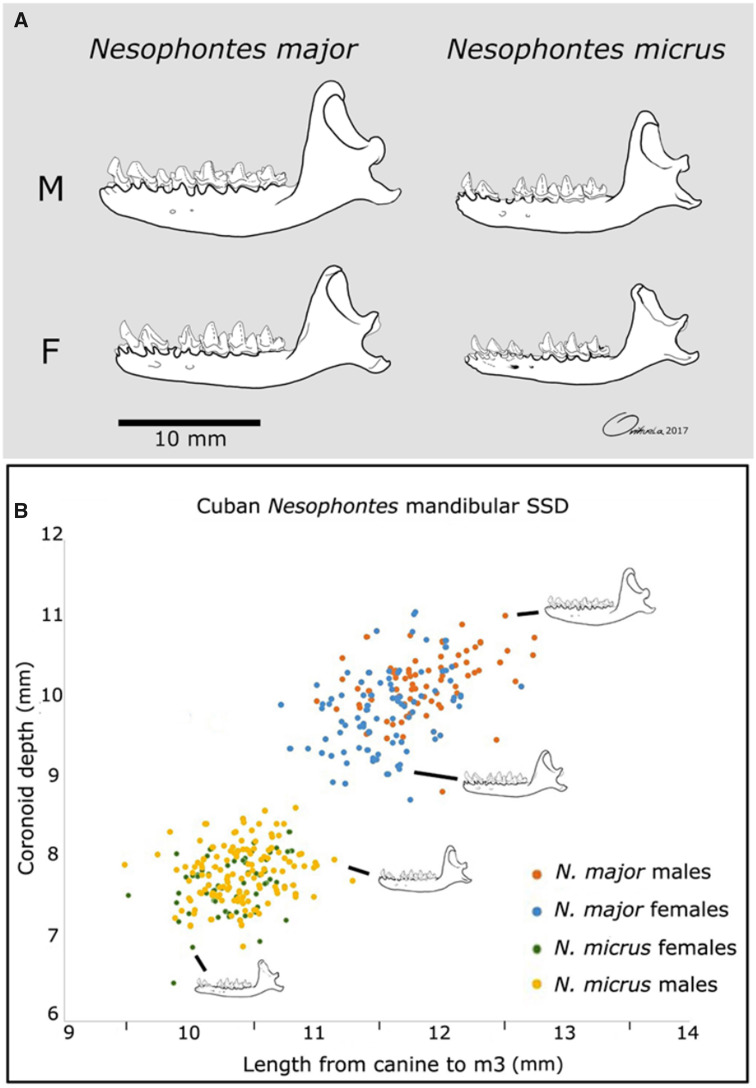
(*A*) Comparison for intraspecific dimorphs between the Cuban *Nesophontes major* and *N. micrus* (see supplementary table S4, Supplementary Material online, for mandible sizes of the two sexes) and (*B*) plot of discriminant mandibular measurements in Cuban *Nesophontes* species and their supposed sexes.

The specimens we selected for our testing sample were identified as possible male and female morphs of each of the Cuban species *N. micrus* and *N. major* (see fig. 4). These specimens fall within previously defined parameters by Condis Fernández et al. (2005), Rzebik-Kowalska and Wołoszyn (2012), and Silva et al. (2007), and these are illustrated in figure 4 and supplementary table S2, Supplementary Material online. These sexual morphs are defined on the basis that within each species there are both large and more robust specimens, plus smaller and gracile specimens (as shown for *N. micrus* and *N. major* from Cuba in fig. 4).

Our analyses support that both robust and gracile morphs within the *N. micrus* specimens are the same, whereas they are separable in peptide sequences from both morphs of *N. major*. This supports the fact that both *N. major* and *N. micrus* are perfectly valid species and that these gracile/robust differences observed within each taxon are likely intraspecific or SSD variation.

## Discussion

### *Nesophontes* Phylogeny in Relation to Geotectonic and Sea-Level History

Our phylogenetic analyses of the available collagen protein sequence data reveal two main clades of *Nesophontes* (fig. 3): one that includes *N. micrus* as sister to *N. major* of Cuba and *N. hemicingulus* of Cayman Brac, and a second that includes Puerto Rican *N. edithae* as sister to the three Hispaniolan species. This topology is consistent with geotectonic and sea-level history of the Greater Antilles as seen in previous studies in Jamaica and the northern Caribbean (Buskirk 1985). The connection between northeastern Hispaniola and Puerto Rico was severed 20-30 Ma by the formation of the Mona Passage (MacPhee et al. 2003). Then, 17–14 Ma, western Hispaniola and Cuba split from each other, which suggests a vicariant origin for the Hispaniolan clade (Iturralde-Vinent 2006), and likely also for *N. edithae* of Puerto Rico. The northern and southern paleoislands of Hispaniola docked ∼15-10 Ma, forming the modern island’s configuration and generating opportunities for speciation with fluctuating Pliocene-Pleistocene sea levels (Iturralde-Vinent 2006).

Until ∼8–6 Ma with the closing of the Havana-Matanzas channel, Cuba was a set of three islands that periodically connected and separated (Iturralde-Vinent 2006), perhaps facilitating the allopatric evolution of *N. micrus* and *N. major*, followed by subsequent secondary contact. Despite their proximity to Cuba, the Cayman Islands never connected, and all faunal and floral colonization has been through overwater dispersals (the three Cayman Islands are peaks on a submerged ridge that rose in the Late Miocene, ∼10 Ma; Jones 1994). Therefore, the earliest that *N. hemicingulus* could have diverged from *N. major* was at this time, which agrees with the very low number of amino acid substitutions between them (most likely within the last few million years according to our analysis; fig. 3). Although our proteomic-only (i.e., partial protein sequence) data do not strongly favor either a Cuban or Hispaniolan origin for Nesophontidae, genetic analysis of ameivas (Tucker et al. 2017) and hutias (Fabre et al. 2014) suggest a Hispaniolan origin, with subsequent diversification east to west into Cuba and the Bahamas along major hurricane tracks and currents (Silva et al. 2007). This is to some extent consistent with our observation that the Cayman species (*N. hemicingulus*) has the fewest number of amino acid substitutions from its closest relative than any other pair of nesophontids from distinct islands but we acknowledge that this apparent late arrival could be due to other reasons (e.g., Pliocene climatic changes). Nevertheless, both studies mentioned above and others (Matos-Maraví et al. 2014) suggest that 14–9 Ma was a time of increased diversification, which also appears to be true for *Nesophontes* based on our findings.

**Fig. 5.**
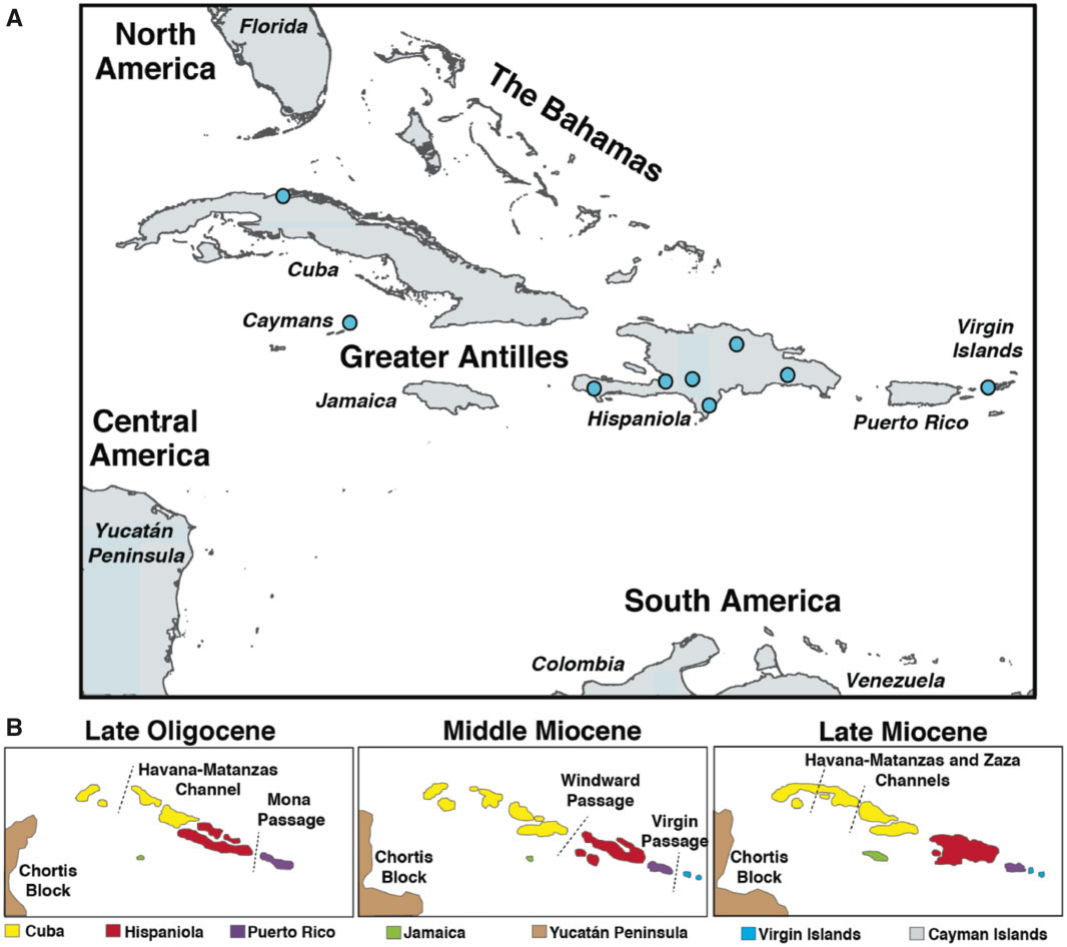
Sampling localities (*A*) and geologic history of the Caribbean (*B*), map information sourced from Cooke et al. (2011), Fabre et al. (2014), Matos-Maraví et al. (2014) and Svenson and Rodrigues (2017).

The biogeographic history of Greater Antillean insectivores has been at the center of an enduring debate, complicated by a dearth of pre-Quaternary fossils, and until recently, a lack of molecular data. Unlike the majority of Caribbean mammals, Solenodonota has been considered to likely have a North American origin due to morphological similarities with other Holarctic taxa including *Asioryctes*, *Cimolestes*, *Batodon*, and the Apternodontidae (Matthew 1918; McDowell 1958; Asher et al. 2002) and its estimated Mesozoic divergence time (∼70 Ma by Springer et al. 2018) is coincident with the positioning of the Cretaceous Island Arc (proto-Antilles) between North and South America, abutting the Chortis block in Mexico (Donnelly 1989), thus providing an opportunity for vicariance and/or short distance dispersal (fig. 5). However, this is relying on an age estimation slightly beyond the oldest extreme of the 44–69 Ma estimate suggested by DNA sequencing (Brace et al. 2016) and far beyond those based on our protein data, the latter implying a much younger divergence.

Following periods of oceanic inundation in which varying parts of the Greater Antillean islands would have been above water, ∼50 Ma marked a time of volcanic activity and movement of Cuba/Hispaniola away from North America (Svenson and Rodrigues 2017). At this time (∼40 Ma), the analysis combining mitochondrial and nuclear DNA sequences (Brace et al. 2016) indicated that Nesophontidae split from Solenodontidae, suggesting that their common ancestor arrived in the intermittent volcanic islands of the proto-Caribbean or the incipient islands of the Caribbean middle Paleogene (the latter being most likely; but two dispersal events, one for each species, cannot be ruled out at present). Perhaps both *Solenodon* and *Nesophontes* or their closest ancestor reached the Caribbean during the latest Eocene or later, but not before due to geologic instability (Draper and Barros 1994).

Although much debate has focused on the arrival of Solenodonota into the Caribbean, the collagen sequences reported here provide new insight into the evolutionary and ecological dynamics following the arrival of this lineage, and, the intra-Antilles radiation of Nesophontidae. Genetic data have suggested an overwater dispersal of *Solenodon* within the Greater Antilles, resulting in the split between Cuban and Hispaniolan solenodons ∼5 Ma (Springer et al. 2018). However, little attention has been paid to within- and between-island divergences of the Nesophontidae. Although collagen evolution is relatively slow, with approximately one amino acid variation per million years (in rodents; Buckley 2018), our analyses suggest rather rapid speciation events for *Nesophontes*, whereas the separation between *Nesophontes* and *Solenodon* remains consistent with that seen from the DNA sequencing at ∼40 Ma (this is at the older end of our estimated range, but as could be expected given the incompleteness of proteomic sequence data). Our data may potentially be biased at the order-level branching because all members of the Solenodonota are represented by proteomics-derived partial sequence information (despite attempts at retrieving sequence information from the recently published genome, yielding only 16% coverage of the two collagen genes of interest; Casewell et al. 2019 ). However, the heterogeneous nature of the matched peptide sequences makes this issue difficult to resolve without substantial losses of sequence information.

Interestingly, our timetree does appear to show similar or even greater evolutionary divergences between several nesophontids than between the two extant solenodons that have most recently been considered distinct genera (*Solenodon paradoxus* and *Atopgale cubana*); on grounds of evolutionary distances, similar arguments could be made for the existence of potentially four genera (e.g., *N. micrus*, *N. major* and *N. hemicingulus*, *N. edithae* and the remaining three hispaniolan species). However, given that this remains unclear for extant taxa, it is unrealistic to make such considerations here in more detail for these extinct taxa. Nevertheless, we also acknowledge that date estimates of divergence do not necessarily relate to speciation, or distinctions of genera.

### Sexual Dimorphism and the Creation of Unnecessary Species

Sexual size dimorphism is common in large mammal species and is typically associated with polygynous mating systems, which is often attributed to one of two causes: sexual selection (competition for mates) or natural selection (where different sexes may face different evolutionary pressures). This is not expected to be present in extremely small mammals, which already face life-history trade-offs associated with thermoregulation and high basal metabolic rates (Weckerly 1998). However, SSD has been assumed in *Nesophontes* since its original description as the underlying cause of body size variation observable in *Nesophontes* and subsequently employed in the diagnosis of several species (Anthony 1916, 1918).

To convincingly diagnose SSD in the fossil record for extinct species, quantitative methods are recommended that include a baseline assessment of SSD in modern relatives (Rehg and Leigh 1999). The closest ecological analog for *Nesophontes* is generally considered to be the true shrews (Soricidae), based on their body size and morphology. The common shrew (*Sorex araneus*) exhibits negligible morphological differences between the sexes (Polly 2007), with the only discernible differences in structures relating to reproductive function such as the pelvis (Brown and Twigg 1970) and the size of the lateral scent gland (Searle 2009). However, White and Searle (2009) recovered minor signatures of sexual dimorphism in *Sorex araneus* where the mechanical potential of the mandible was 1.3% greater in males than in females, potentially implying a greater bite force for males via a longer coronoid-condyle length. This in turn resembles what is observable in Cuban *Nesophontes* where the larger and more robust morphs (the supposed males) have more pronounced maxillomandibular muscle scars than the gracile morphs. Moreover, each taxon has its own set of gracile–robust pairs that do not merge morphologically or chemically in terms of collagen peptides (fig. 4).

Conflicting expectations have been reported for island vertebrates regarding an increase or decrease in SSD, particularly in the context of population densities and reduced interspecific competition for resources. Following the “Island Syndrome,” some researchers have suggested that high population densities in a stable island environment and in the absence of predators (e.g., density compensation) should lead to decreased aggressiveness, reproductive output, and SSD (Adler and Levins 1994). Alternatively, others suggest that SSD should be greater in insular populations, as decreased interspecific competition allows sexes to diverge in trophic characters and occupy vacant ecomorphological space (Greenberg and Danner 2013). In the Greater Antilles, SSD has been shown to operate as the functional equivalent of increased ecomorphological diversity for the endemic radiation of *Anolis* lizards, where increased dimorphism between the sexes acts as an alternative route to achieving an ecological radiation (Butler et al. 2007). If this were operating in *Nesophontes*, we might expect to see the greatest sexual dimorphism in the Puerto Rican *N. edithae*, as it was the only eulipotyphlan on the island (and *Solenodon* is absent). Our molecular phylogeny lays the foundation for testing this and additional hypotheses regarding the tension between sexual and natural selection in this unique island laboratory through further accumulation and investigation of morphological data.

## Conclusions

Understanding relationships between extinct taxa can be difficult and is primarily carried out through the analysis of morphological characters. However, such investigations are limited by the survival or preservation of skeletal elements and the recognition of diagnostic features in the fossil record. Molecular methods aimed to overcome these limitations originally focused on DNA-based approaches following the introduction of the polymerase chain reaction to molecular biology, which allowed for the amplification of minute amounts of DNA into much greater quantities (Pääbo 1989). However, DNA preservation is notoriously poor in ancient remains from the tropics (Gutiérrez-García et al. 2014). Upon providing both near-complete and unique collagen sequences for most of the known nesophontid species present in the Caribbean, this study provides great insight into the evolution and biogeography of *Nesophontes*. Our results support the hypothesis that there are only two nesophontid species known from Cuba, *N. major*, and *N. minor*, and that both species exhibit population variation, perhaps attributable to sexual dimorphism. Conversely, the Hispaniolan species are valid as a distinct clade, even though two of them (*N. hypomicrus* and *N. zamicrus*) appear conspecific, potentially like the situation with *N. micrus* and *N. submicrus*. Interestingly, the specimen from Cayman Brac (*N. hemicingulus*) is also validated as a different species from the Cuban species, and that it likely originated from an *N. major*-like ancestor. Proteomic sequencing has enabled us to improve our understanding of the origin, evolution, and distribution of this extinct mammal lineage. Such knowledge is vital for this biodiversity hotspot, where the magnitude of recent extinctions may obscure true estimates of species richness in the past.

## Materials and Methods

Collagen was extracted using 0.3 M hydrochloric acid following the minimally destructive approach of Buckley et al. (2016) for 3 h and the acid-soluble collagen transferred into 50 mM ammonium bicarbonate using 30-kDa ultrafilters following Van der Sluis et al. (2014). Samples were then digested with 0.4 µg sequencing grade trypsin (Promega, UK) overnight at 37 °C. The peptide digests were then fractionated into 10% and 50% acetonitrile (in 0.1% trifluoroacetic acid; TFA), evaporated, resuspended in 0.1% TFA and spotted onto a stainless steel target plate for fingerprint analysis using a Brüker Ultraflex II Matrix-Assisted Laser Desorption Ionization Time of Flight (MALDI-ToF) mass spectrometer following Buckley et al. (2009). Half of each aliquot was then combined and 2 µl subjected to in-depth sequencing by LC-Orbitrap Elite mass spectrometric analysis. Sequencing was carried out using an UltiMate 3000 Rapid Separation LC (RSLC, Dionex Corporation, Sunnyvale, CA) coupled to an Orbitrap Elite (Thermo Fisher Scientific, Waltham, MA) mass spectrometer (120 k resolution, full scan, positive mode, normal mass range 350–1,500) following analytical methods described by Wadsworth and Buckley (2014). Primarily, sequences were recovered via error-tolerant searches against a local database (Buckley 2015) that included the concatenated COL1A1 and COL1A2 sequences for the three eulipotyphlans available from a protein BLAST search of rat collagen. These sequences were then ordered by position and manually aligned in BioEdit Sequence Alignment Editor V.7.1.3.0 with X representing unknown/unmatched amino acid residues following Buckley (2013). Phylogenetic analyses of the concatenated collagen alpha 1 and alpha 2 sequences from the seven *Nesophontes* and one *Solenodon* analyses (yielding a total length of 2,098 amino acid residues) were then carried out with 46 other mammalian type 1 collagen sequences (concatenated chains) obtained from the Ensembl databases and the protein BLAST searches, including the only known eulipotyphlan sequences (shrew [*Sorex araneus*], mole [*Condylura cristata*], and hedgehog [*Erinaeus europaeus*]) with Tasmanian devil (*Sarcophilus harrisii*) included for use as the outgroup (see supplementary table S6, Supplementary Material online).

We used Partition Finder 2.1.1 (Lanfear et al. 2016) to identify a suitable model and partition scheme for the data. The data were analyzed using unlinked branch lengths, “MrBayes only” models, AICc model selection, and the “greedy” search algorithm. The best scheme identified by PartitionFinder was a single partition analyzed using Dayhoff + invgamma. A topology search was undertaken using MrBayes 3.2.7 (Ronquist et al. 2012). For the purposes of the current study, we were concerned only with the topology within Eulipotyphla. As such, we used a total of 19 topological constraints (see supplementary nexus files, Supplementary Material online) to ensure that the rest of the tree was consistent with previously published phylogenomic studies (dos Reis et al. 2012; Tarver et al. 2016). We also conducted a second analysis with an additional topological constraint for *Sorex* + *Erinaceus*. We used four runs of 5,000,000 generations, sampling every 500 generations and discarding the first 25% of the sample as burnin. Convergence was assessed using Tracer 1.7 (Rambaut et al. 2018). We computed the 50% Majority Rule Consensus (MRC) tree and Maximum Clade Credibility (MCC) tree.

To investigate the timing of the *Nesophontes* divergence, we dated the MCC trees obtained from our *Sorex* unconstrained and *Sorex* constrained analyses using MCMCtree (Yang 2007). We used 12 soft fossil calibrations obtained from Benton et al. (2015) and Phillips (2015) (see supplementary table S7, Supplementary Material online). Prior distributions were defined using the “estimateSkewT” function in the MCMCtreeR package (Puttick 2019). We sampled 10,000 trees using a burnin of 50,000,000 generations, sampling every 2,000 generations. The resulting timetree was plotted using the “MCMC.tree.plot” function in MCMCtreeR (Puttick 2019).

Morphological measurements were taken with a digital caliper and are reported in millimeters (mm). All statistical analyses were conducted with the software PAST v3 and STATISTICA software (1995, v5). Two-way ANOVAs and Tukey’s test for unequal sample sizes were used to compare linear measurements between species. Principal component analysis (PCA) was performed to further explore differences between Cuban *Nesophontes* taxa and their sexual morphs. Probabilities were compared with a significance level of alpha <0.05, and of <0.01 for the PCA. These data were plotted using STATISTICA (1995) and Excel (Orihuela J, unpublished data).

## Data Availability

Raw proteomic files have been made available via proteomeXchange with identifier PXD01846.

## Supplementary Material

msaa137_supplementary_data
