# Supplementary material for: Collagen Sequence Analysis Reveals Evolutionary History of Extinct West Indies Nesophontes (Island-Shrews)
Source: Mol Biol Evol. 2020 Jun 4;37(10):2931–43. doi: 10.1093/molbev/msaa137 (PMC7530613; doi:10.1093/molbev/msaa137)
Supplement: msaa137_supplementary_data [file msaa137_supplementary_data.zip › Nesophontes_Table_S1.pdf]

Specimen	Species	Origin	Location Information	Element	Source	Fail
UF 128167	*S. paradoxus*	Haiti	Trouing Jeremie #1 (Forom)	R. mandible	FLMNH	
UF 128165	*S. paradoxus*	Haiti	Trouing Jeremie #1 (Forom)	L. mandible	FLMNH	X
UF 134729	*S. paradoxus*	Haiti	Trouing Marassa (La Visite)	R. mandible	FLMNH	
UF 134734	*S. paradoxus*	Haiti	Loc. Trouing Marassa (La Visite)	R. maxilla	FLMNH	
UF 278430	*S. paradoxus*	DR	Rancho la Guardia (XD002;Elias Pina Provincia Co.)	L. mandible	FLMNH	X
UF 128163	*S. paradoxus*	Haiti	Trouing Jeremie #1(Foom)	Tooth	FLMNH	X
UF 278431	*S. paradoxus*	DR	Rancho la Guardia (XD002), Elias Pina Provincia Co.	L. mandible	FLMNH	X
UF 134182	*S. paradoxus*	Haiti	Trouing Titwe #2 (Cavalier Plateau)	Tooth Rm3	FLMNH	X
UF 128168	*S. paradoxus*	Haiti	Trouing Jeremie #1 (Forom)	L. mandible	FLMNH	X
	S. paradoxus	Cuba	Cueva de la Caja o Nesofontes (Mayabeque)	Scapula		
JO_150	*N. micrus*	Cuba	Cueva de la Caja o Nesofontes (Mayabeque)	L. mandible	MNHN	
JO_407	*N. micrus*	Cuba	Cueva del Gato Jíbaro (Matanzas)	R. mandible	MNHN	
JO_576	*N. major*	Cuba	Cueva de la Caja o Nesofontes (Mayabeque)	R. mandible	MNHN	
JO_ M381	*N. major*	Cuba	Cueva del Gato Jíbaro (Matanzas)	L. mandible	MNHN	
PF19	*'N. B'*	CB	Patton's Fissure	L. mandible	UoM	
MS2 0.1.952	*N. edithae*	VI	Magens Bay, St. Thomas, US Virgin Islands (The Gudmund Hatt archaeological collection; *Artefact No: O.1.952, Unit 1, Square VII)*	Partial skull	NMD	
FOS 25 409	*N. paramicrus*	DR	Cayacoa, Santo Domningo Este, Boca Chica	Mandible	MNHN	X
FOS 25 422	*N. paramicrus*	DR	Cayacoa (Santo Domningo Este, Boca Chica)	Mandible	MNHN	
UF 74911	*N. zamicrus*	Haiti	Trouing Jean Paul (La Visite)	R. mandible	FLMNH	
UF 24918	*N. zamicrus*	Haiti	Trouing Jean Paul (La Visite)	R. mandible	FLMNH	
FOS 25 418	*N. hypomicrus*	DR	Pedernales (Fondo Paradi; Cueva #24)	Mandible	MNHN	X
FOS 25 407	*N. hypomicrus*	DR	Pedernales, Cueva de Mono, (manuel Goya,Oviedo)	Long bone fragment	MNHN	X
FOS 25 408	*N. hypomicrus*	DR	Cueva de Mono (Oviedo, Pedernales, Manuel Goya)	Fragment	MNHN	X
FOS 25 403	*N. hypomicrus*	DR	Cueva de Toupiti (Pedernales, Tres Charcoals)	Femur	MNHN	X
FOS 25 404	*N. hypomicrus*	DR	Cueva del Campanario (Villa Gonzalez, Santiago)	Femur	MNHN	X
FOS 25 406	*N. hypomicrus*	DR	Cueva de Mono (Oviedo, Pedernales, Manuel Goya)	Fragment	MNHN	X
FOS 25 405	*N. hypomicrus*	DR	Cueva de Mono (Oviedo, Pedernales, Manuel Goya)	Long bone	MNHN	X
