# Supplementary material for: Collagen Sequence Analysis Reveals Evolutionary History of Extinct West Indies Nesophontes (Island-Shrews)
Source: Mol Biol Evol. 2020 Jun 4;37(10):2931–43. doi: 10.1093/molbev/msaa137 (PMC7530613; doi:10.1093/molbev/msaa137)
Supplement: msaa137_supplementary_data [file msaa137_supplementary_data.zip › Nesophontes_Table_S2.pdf]

Measurements*	*N. micrus*		*N. major*	
	Female	Male	Female	Male
Total mandibular length	17.95±0.684 (16.39-19.18) 55	18.16±0.431 (17.0-19.09) 132	20.35±0.542 (19.37-21.44) 72	21.04±0.402 (19.61-22.29) 82
Total dental length	11.47±0.318 (10.18-11.9) 55	11.53±0.284 (10.77-12.45) 132	12.49±0.436 (11.3-13.67) 72	13.14±0.451 (11.44-14.4) 82
Length from Canine to m3**	10.35±0.272 (9.52-10.85) 55	10.43±0.303 (9.49-11.22) 132	11.42±0.504 (9.99-12.66) 72	11.85±0.321 (11.02-12.76) 82
Coronoid Height**	7.65±0.347 (6.47-8.36) 55	7.83±0.362 (6.93-8.74) 132	9.80±0.442 (8.76-10.85) 72	10.18±0.344 (9.53-11.05) 82
Ramus height at m2**	2.22±0.178 (1.65-2.58) 55	2.31±0.171 (1.77-2.89) 132	2.66±0.203(2.66-3.45) 72	3.16±0.206 (2.66-3.74) 82

Sequence: mean ± SD (range min-max) number. All measurements are given in mm to the nearest hundredths. All these measurements (*) where differences are significant (*P* > 0.05) between the species. And **=measurements where differences are significant (*P* > 0.05) between the assumed sexes. Results from two-way ANOVA with post hoc Tukey tests analyzed on PAST statistical software. All specimens analyzed are from a single locality (Cueva de los Nesofontes, Mayabeque, Cuba) and are Late Holocene (pre-Columbian) in age.
