# Supplementary material for: Collagen Sequence Analysis Reveals Evolutionary History of Extinct West Indies Nesophontes (Island-Shrews)
Source: Mol Biol Evol. 2020 Jun 4;37(10):2931–43. doi: 10.1093/molbev/msaa137 (PMC7530613; doi:10.1093/molbev/msaa137)
Supplement: msaa137_supplementary_data [file msaa137_supplementary_data.zip › Nesophontes_Table_S3.pdf]

Species	No. Differences COL1A1	No. Differences COL1A2
Condylura (from *Sorex*)	18	61
Erinaceus (from *Sorex*)	16	58
Myotis brandti (from *Myotis lucifugus*)	0	2
Eptesicus fuscus (from *Myotis lucifugus*)	4	6
Miniopterus natalensis (from *Myotis lucifugus*)	11	37
