# Supplementary material for: Collagen Sequence Analysis Reveals Evolutionary History of Extinct West Indies Nesophontes (Island-Shrews)
Source: Mol Biol Evol. 2020 Jun 4;37(10):2931–43. doi: 10.1093/molbev/msaa137 (PMC7530613; doi:10.1093/molbev/msaa137)
Supplement: msaa137_supplementary_data [file msaa137_supplementary_data.zip › Nesophontes_TableS4.pdf]

Peptide	1t28	1t30	1t37	1t41	1t47
Position	**312-326**	**333-344**	**404-413**	**438-451**	**525-536**
N. major	GEPGPTGLPGPPGER	GFPGSDGAAGPK	PGPAGPPGAR	GVPGPPGAVGPAGK	GVQGPPGPAGPR
N. B	- - - - - - - - - - - - - - -	- - - - - - - - - - - -	- - - - - - - - - -	- - - - - - - - - - - - - -	- - - - - - - - - - - -
N. micrus	- - - - - - - - S - - - - - -	- - - - - - - - - - - -	- - - - - - - - - -	- - - - - - - - - - - - - -	- - - - - - - - - - - -
N. zamicrus	- - - - - - - - S - - - - - -	- - - - - - - - - - - -	- - - - - - - - - -	- - - - - - - - I - - - - -	- - - - - - - - - - - -
N. hypomicrus	- - - - - - - - S - - - - - -	- - - - - - - - - - - -	- - - - - - - - - -	- - - - - - - - I - - - - -	- - - - - - - - - - - -
N. paramicrus	- - - - - - - - S - - - - - -	- - - - - - - - - - - -	- - - - - - - - - -	- - - - - - - - I - - - - -	- - - - - - - - G - - -
N. edithae	- - - - - - - - S - - - - - -	- - - - A - - - - - - -	- - - P - - - - - -	- - - - - - - - - - - - - -	- - - - - - - - - - - -
Peptide	1t56	1t62	1t63	1t67	1t77
Position	**621-635**	**705-721**	**722-742**	**758-773**	**876-901**
N. major	GESGPSGPAGPTGAR	GSAGPPGATGFPGAAGR	VGPPGPSGNAGPPGPPGPVGK	PGEVGPPGPPGPAGEK	GETGPAGPPGAPGAPGAPGPVGPAGK
N. B	- - - - - - - - - - - - - - -	- G - - - - - - - - - - - - - - -	- -	- - - - - - - - - - - - - - - -	- -
N. micrus	- - - - - - - - - - - - - - -	- - - - - - - - - - - - - - - - -	- -	- - - - - - - - - - - - T - - -	- -
N. zamicrus	- - - - - - - - - - - - - - -	- - - - - - - - - - - - - - - - -	- -	- - - - - - - - - - - - - - - -	- -
N. hypomicrus	- - - - - - - - - - - - - - -	- - - - - - - - - - - - - - - - -	- -	- - - - - - - - - - - - - - - -	- -
N. paramicrus	- - - - - - - - - - - - - - -	- - - - - - - - - - - - - - - - -	- - - - - - - - G - - - - - - - - - - - -	- - - - - - - - - - - - - - - -	- -
N. edithae	- - - - - - - - G - - - - - -	- - - - - - - - - - - - - - - - -	- -	- - - - - - - - - - - - - - - -	- - - - - - - - - - - - - T - - - - - - - - - - - -
Peptide	2t21	2t26	2t54	2t76	
Position	**1307-1321**	**1361-1378**	**1673-1687**	**1958-1975**	
N. major	GIPGPVGSAGASGAR	GSNGEPGSAGPTGPPGLR	GEAGPSGPAGPAGPR	GETGPAGVVGPVGGFGPR	
N. B	- - - - - - - - - - - - - - -	- - - - - - - - - - - - - - - - - -	- - - - - - - - - - - - - - -	- - - - - - - - - - - - - - - - - -	
N. micrus	- - - - - - - - - - - - - - -	- - - - - - - - T - - - - - - - - -	- - - - - - - - - - - - - - -	- - - - - - - - - - - - - - - - - -	
N. zamicrus	- - - - - - - - - - - - - - -	- - - - - - - - - - - - - - - - - -	- - - - - - - - - - - - - - -	- - - - - - - A - - - - - - - - - -	
N. hypomicrus	- - - - - - - - - - - - - - -	- - - - - - - - - - - - - - - - - -	- - - - - - - - - - - - - - -	- - - - - - - A - - - - - - - - - -	
N. paramicrus	- - - - - - - - - - - - - P -	- - - - - - - - - - - - - - - - - -	- - - - S - - - - - - - - - -	- - - - - - - A - - - - - - - - - -	
N. edithae	- - - - - - - - - - - - - - -	- - - - - - - - S - - - - - - - - -	- - - - - - - - - - - - - - -	- - - - - - - A - - - - - - - - - -	
