# Supplementary material for: Collagen Sequence Analysis Reveals Evolutionary History of Extinct West Indies Nesophontes (Island-Shrews)
Source: Mol Biol Evol. 2020 Jun 4;37(10):2931–43. doi: 10.1093/molbev/msaa137 (PMC7530613; doi:10.1093/molbev/msaa137)
Supplement: msaa137_supplementary_data [file msaa137_supplementary_data.zip › Nesophontes_TableS5.pdf]

Node	Sorex unconstrained		Sorex constrained	
	Mean	95% HPD	Mean	95% HPD
N. paramicrus-zamicrus-hypomicrus	2.24	0.6-4.85	1.89	0.5-4.04
N. paramicrus-zamicrus-hypomicrus-edithae	3.81	1.7-6.94	3.12	1.3-5.85
N. hemicingulus-major	1.17	0.08-3.37	1.69	0.61-6.85
Nesophontes	5.31	2.71-9.35	5.88	2.93-10.64
Solenodon-Nesophontes	27	16.05-40.71	29.86	17.98-46.28
Eulipotyphla	57.42	46.91-69.94	55.72	45.6-67.28
