# Supplementary material for: Collagen Sequence Analysis Reveals Evolutionary History of Extinct West Indies Nesophontes (Island-Shrews)
Source: Mol Biol Evol. 2020 Jun 4;37(10):2931–43. doi: 10.1093/molbev/msaa137 (PMC7530613; doi:10.1093/molbev/msaa137)
Supplement: msaa137_supplementary_data [file msaa137_supplementary_data.zip › Nesophontes_TableS6.pdf]

>Sorex

QMSYGYDEKSTGG-ISVPGPMGPSGPRGLPGPPGAPGPQGFQGPPGEPGEPGASGPMGPR

GPAGPPGKNGDDGEAGKPGRPGERGPPGPQGARGLPGTAGLPGMKGHRGFSGLDGAKGDS

GPAGPKGEPGSPGENGAPGQMGPRGLPGERGRPGAPGPAGARGNDGATGAAGPPGPTGPA

GPPGFPGAVGAKGEAGPQGARGSEGPQGVRGEPGPPGPAGAAGPAGNPGADGQPGAKGAN

GAPGIAGAPGFPGARGPSGPQGPSGPPGPKGNSGEPGAPGNKGDTGAKGEPGPAGVQGPP

GPAGEEGKRGARGEPGPTGLPGPPGERGGPGGRGFPGADGAAGPKGPAGERGSPGPAGPK

GSPGEAGRPGEAGLPGAKGLTGSPGSPGPDGKTGPPGPAGQDGRPGPPGPPGARGQAGVM

GFPGPKGAAGEPGKAGERGVPGPPGAVGPAGKDGEAGAQGAPGPAGPAGERGEQGPAGSP

GFQGLPGPAGPPGEAGKPGEQGAPGDLGAPGPSGARGERGFPGERGVQGPPGPAGPRGSN

GAPGNDGAKGDAGAPGAPGSQGAPGLQGMPGERGAAGLPGPKGDRGDAGPKGADGSPGKD

GVRGLTGPIGPPGPAGAPGDKGESGPSGPAGPTGARGAPGDRGEPGPPGPAGFAGPPGAD

GQPGAKGEPGDAGAKGDAGPPGPAGPTGAPGPIGNVGAPGPKGARGSAGPPGATGFPGAA

GRVGPPGPSGNAGPPGPPGPVGKEGGKGPRGETGPAGRPGEVGPPGPPGPAGEKGSPGAD

GPAGSPGTPGPQGIAGQRGVVGLPGQRGERGFPGLPGPSGEPGKQGPSGSSGERGPPGPM

GPPGLAGPPGESGREGSPGAEGSPGRDGSPGPKGDRGETGPAGPPGAPGAPGAPGPVGPA

GKSGDRGETGPAGPAGPIGPAGARGPAGPQGPRGDKGETGEQGDRGMKGHRGFSGLQGPP

GPPGSPGEQGPSGASGPAGPRGPPGSAGAAGKDGLNGLPGPIGPPGPRGRTGDAGPVGPP

GPPGPPGPPGPPSGGFDFSFMPQPPQEKAHDGGRYYRAQYD-GKGVGLGPGPMGLMGPRG

PPGASGAPGPQGFQGPAGEPGEPGQTGPAGARGPPGPPGKAGEDGHPGKAGRPGERGVVG

PQGARGFPGTPGLPGFKGIRGHNGLDGLKGQPGAPGVKGEPGAPGENGTPGQAGARGLPG

ERGRVGAPGPAGARGSDGSVGPVGPAGAIGSAGPPGFPGAPGPKGELGAVGNPGPAGPAG

ARGEVGLPGVSGPVGPAGNPGANGLTGAKGAAGLPGVAGAPGLPGPRGIPGPVGAAGASG

PRGLIGEPGPAGSKGEGGNKGEPGSAGPQGPPGPSGEEGKRGQNGEPGSAGPTGPPGLRG

TPGSRGLPGADGRPGVMGPPGSRGASGPAGARGPNGDSGRPGEPGLVGPRGFPGSPGSVG

PAGKEGPVGLPGIEGRPGAIGPAGARGEPGNIGFPGPKGPNGEPGKSGDKGHPGLAGARG

APGPDGNNGAQGPPGPQGVQGGKGEQGPAGPPGFQGLPGPAGTTGEVGKPGERGLPGEFG

LPGPAGPRGERGPPGESGAAGPAGPIGSRGPSGPPGPDGNKGEPGVVGAPGNAGPSGPGG

LPGERGAAGIPGGKGEKGETGLRGEIGNPGRDGARGAPGAVGAPGPSGAAGDRGEAGAAG

PAGPAGPRGSPGERGEVGPAGPNGFAGPAGAAGQPGAKGERGTKGPKGENGVVGPTGPVG

AAGPSGPNGPPGPAGSRGDGGPPGATGFPGAAGRTGPPGPSGITGPPGPPGAAGKEGLRG

PRGDQGPVGRTGETGASGLPGFAGEKGPNGEPGTAGPPGTPGPQGLLGAPGILGLPGSRG

ERGLPGIAGSVGEPGPLGISGPPGARGPPGAVGNPGVNGAPGEAGRDGNPGSDGPPGRDG

QPGHKGERGYPGNAGPVGAVGAPGPHGPVGPTGKHGNRGEPGPAGAVGPAGAFGPRGPSG

PQGIRGDKGEPGDKGARGLPGLKGHNGLQGLPGLAGHHGDQGAPGSVGPAGPRGPAGPSG

PAGKDGRSGHPGTVGPAGIRGSQGNQGPAGPPGPPGPPGPPGPSGGGYDFGYDGDFYRA

>Condylura

QMSYGYDEKSAGG-ISVPGPMGPSGPRGLPGPPGAPGPQGFQGPPGEPGEPGASGPMGPR

GPPGPPGKNGDDGEAGKPGRPGDRGPPGPQGARGLPGTAGLPGMKGHRGFSGLDGAKGDS

GPAGPKGEPGSPGENGAPGQMGPRGLPGERGRPGPSGPAGARGNDGATGAAGPPGPTGPA

GPPGFPGAVGAKGEAGPQGARGSEGPQGVRGEPGPPGPAGAAGPAGNPGADGQPGAKGAN

GAPGIAGAPGFPGARGPSGPQGPSGPPGPKGNSGEPGAPGNKGDTGAKGEPGPTGVQGPP

GPAGEEGKRGARGEPGPTGLPGPPGERGGPGSRGFPGSDGAAGPKGPAGERGSPGPAGPK

GSPGEAGRPGEAGLPGAKGLTGSPGSPGPDGKTGPPGPAGQDGRPGPPGPPGARGQAGVM

GFPGPKGAAGEPGKAGERGVPGPPGAVGAAGKDGEAGAQGPPGPAGPAGERGEQGPAGSP

GFQGLPGPAGPPGEAGKPGEQGAPGDLGAPGPSGARGERGFPGERGVQGPPGPAGPRGSN

GAPGNDGAKGDAGAPGAPGSQGAPGLQGMPGERGAAGLPGPKGDRGDAGPKGADGSPGKD

GVRGLTGPIGPPGPAGAPGDKGESGPSGPAGPTGARGAPGDRGEPGPPGPAGFAGPPGAD

GQPGAKGEPGDAGAKGDAGAPGPAGPAGPPGPIGNVGAPGPKGARGSAGPPGATGFPGAA

GRVGPPGPSGNAGPPGPPGPVGKEGGKGPRGETGPAGRPGEVGPPGPPGPSGEKGSPGAD

GPAGSPGTPGPQGIAGQRGVVGLPGQRGERGFPGLPGPSGEPGKQGPSGSSGERGPPGPM

GPPGLAGPPGESGREGSPGAEGSPGRDGAPGPKGDRGETGPAGPPGAPGAPGAPGPVGPA

GKSGDRGETGPAGPAGPIGPAGARGPAGPQGPRGDKGETGEQGDRGMKGHRGFSGLQGPP

GPPGSPGEQGPSGASGPAGPRGPPGSAGAPGKDGLNGLPGPIGPPGPRGRTGDAGPVGPP

GPPGPPGPPGPPSGGFDFSFLPQPPQEKSHDGGRYYRAQYD-GKGVGLGPGPMGLMGPRG

PPGASGAPGPQGFQGPAGEPGEPGQTGPAGSRGPAGPPGKAGEDGHPGKPGRPGERGVVG

PQGARGFPGTPGLPGFKGIRGHNGLDGLKGQPGAPGVKGEPGAPGENGTPGQTGARGLPG

ERGRVGAPGPAGARGSDGSVGPVGPAGPIGSAGPPGFPGAPGPKGELGAVGNPGPAGPAG

ARGEVGLPGVSGPVGPPGNPGANGLTGAKGAAGLPGVAGAPGLPGPRGIPGPVGSAGASG

ARGLVGEPGPAGSKGETGNKGEPGSAGAQGPPGPSGEEGKRGQNGEAGSTGPTGPPGLRG

NPGSRGLPGADGRPGVMGPPGSRGASGPAGVRGPNGDSGRPGEPGLMGPRGFPGSPGNVG

PTGKEGPVGLPGIDGRPGPIGPAGARGEAGNIGFPGPKGPTGDPGKSGEKGHAGLAGPRG

APGPDGNNGAQGPPGPQGVQGGKGEQGPAGPPGFQGLPGPAGAVGEVGKPGERGLPGEFG

LPGPAGPRGERGPPGESGAAGPSGPIGSRGPSGPPGPDGNKGEPGVVGAPGSPGASGPGG

LPGERGAAGMPGGKGEKGETGLRGEIGNPGRDGARGAPGAVGAPGPAGATGDRGEAGPSG

PAGPAGPRGSPGERGEVGAAGPNGFAGPAGAAGQPGAKGERGTKGPKGENGVVGPMGPVG

AAGPSGPNGPPGPVGGRGDGGPPGVTGFPGAAGRTGPPGPSGITGPPGPPGAAGKEGLRG

PRGDQGPVGRTGETGAAGPPGFTGEKGPSGEPGTAGPPGTPGPQGLLGTPGILGLPGSRG

ERGLPGISGSMGEPGPLGISGPPGARGPPGAVGNPGVNGAPGEAGRDGNPGSDGPPGRDG

QPGHKGDRGYPGNIGPVGAAGAPGPHGNVGPTGKHGNRGEPGPAGVVGPVGAFGPRGPSG

PQGIRGDKGEPGDKGPRGLPGLKGHNGLQGLPGLAGHHGDQGAPGSVGPAGPRGPAGPSG

PAGKDGRSGHPGTVGPAGIRGTQGNQGPAGPPGPPGPPGPPGISGGGYDFGYEGDFYRA

>Erinaceus

QMSYGYDEKSTGG-MSVPGPMGPSGPRGLPGPPGSPGPQGFQGPPGEPGEPGASGPMGPR

GLPGPPGKNGDDGEAGKPGRPGERGPPGPQGARGLPGTAGLPGMKGHRGFSGLDGAKGDS

GPAGPKGEPGSPGENGAPGQMGPRGLPGERGRPGATGPAGARGNDGATGAAGPPGPTGPA

GPPGFPGAVGAKGEAGPQGARGSEGPQGVRGEPGPPGPAGAAGPAGNPGADGQPGAKGAN

GAPGIAGAPGFPGARGPSGPQGPSGPPGPKGNSGEPGAPGNKGDTGAKGEPGPAGVQGPP

GPAGEEGKRGARGEPGPTGLPGPPGERGGPGSRGFPGSDGAAGPKGPAGERGSPGPAGPK

GSPGEAGRPGEAGLPGAKGLTGSPGSPGPDGKTGPPGPAGQDGRPGPPGPPGARGQAGVM

GFPGPKGAAGEPGKAGERGVPGPPGAVGPAGKDGEAGAQGPPGPAGPAGERGEQGPAGSP

GFQGLPGPAGPPGEAGKPGEQGAPGDLGAPGPSGARGERGFPGERGVQGPPGPAGPRGSN

GAPGNDGAKGDAGAPGAPGSQGAPGLQGMPGERGAAGLPGPKGDRGDAGPKGADGSPGKD

GVRGLTGPIGPPGPAGAPGDKGESGPSGPAGPTGARGAPGDRGEPGPPGPAGFAGPPGAD

GQPGAKGEPGDAGAKGDSGPPGPAGPTGPPGPIGNVGAPGPKGARGAAGPPGATGFPGAA

GRVGPPGPSGNAGPPGPPGPVGKEGGKGPRGETGPAGRPGEAGPPGPPGPAGEKGSPGAD

GPAGSPGTPGPQGIAGQRGVVGLPGQRGERGFPGLPGPSGEPGKQGPSGASGERGPPGPM

GPPGLAGPPGESGREGSPGAEGSPGRDGSPGPKGDRGETGPAGPPGAPGAPGAPGPVGPA

GKSGDRGETGPAGPAGPIGPAGARGPAGPQGPRGDKGETGEQGDRGMKGHRGFSGLQGPP

GPPGSPGEQGPSGASGPAGPRGPPGSAGAAGKDGLNGLPGPIGPPGPRGRTGDAGPvGPP

GPPGPPGPPGPPSGGFDLNFLPQPPQEKAHDGGRYYRAQYD-GKGVGLGPGPMGLMGPRG

PPGASGAPGPPGFQGPAGEPGEPGQTGPAGARGPTGPPGKAGEDGHPGKPGRPGERGVVG

PQGARGFPGTPGLPGFKGIRGHNGLDGLKGQPGAPGVKGEPGAPGENGTPGQTGARGLPG

ERGRVGAPGPAGARGSDGSVGPVGPAGPIGSAGPPGFPGAPGPKGELGPVGNPGPSGPAG

ARGEVGLPGVSGPVGPPGNPGANGLTGAKGAAGLPGVAGAPGLPGPRGIPGPVGAAGASG

ARGLVGEPGPAGSKGETGNKGEPGSAGAQGLPGPSGEEGKRGQNGEAGSAGPAGPPGLRG

SPGSRGLPGADGRPGVMGPPGSRGASGPAGVRGPSGDSGRPGEPGLMGPRGFPGSPGNVG

PAGKEGPSGLPGIDGRPGPIGPAGARGEPGNIGFPGPKGPSGDPGKSGDKGHAGLAGARG

APGPDGNNGAQGPPGAQGVQGGKGEQGPAGPPGFQGLPGPAGTTGEVGKPGERGLPGEFG

LPGPAGPRGERGPPGQSGAAGPSGPIGSRGPSGSPGPDGNKGEPGVLGAPGTAGPSGPGG

LPGERGAAGVPGGKGEKGETGLRGEIGNPGRDGARGAPGAIGAPGPSGAAGDRGEAGPAG

PAGPAGPRGSPGERGEVGPAGPNGFAGPAGAAGQPGAKGERGTKGPKGENGIVGPTGPVG

AAGPSGPNGPPGPAGGRGDGGPPGATGFPGAAGRTGPPGPSGITGPPGPPGAAGKEGLRG

PRGDQGPVGRTGETGGSGPPGFTGEKGPAGEPGTAGPPGTAGPQGLLGAPGILGLPGSRG

ERGLPGVFGSVGEPGPLGIAGPPGARGPPGAVGNPGVNGAPGEAGRDGNPGSDGPPGRDG

QPGHKGERGYPGNAGSVGAAGAPGPHGSVGPAGKHGNRGEPGPAGAVGPVGAFGPRGPSG

PQGIRGDKGEPGDKGPRGLPGLKGHNGLQGLPGLAGQHGDQGAPGSVGPAGPRGPAGPSG

PAGKDGxxxxxxxVGPAGIRGsQGNQGPAGPaGPPGPPGPPGPSGGGYDFGYEGDFYRA

>NesophontesMajor

XXXXXXXXXSSGG-MSVPGPMGPSGPRXXXXXXXXXXXXXXXXXXXXXXXXXXXXXXXXX

GPPGPPGKNGDDGEAGKPGRPGERGPPGPQGARGLPGTAGLPGMKGHRGFSGLDGAKGDS

GPAGPKGEPGSPGENGAPGQMGPRXXXXXXGRPGAPGSAGARGNDGATGAAGPPGPTGPA

GPPGFPGAVGAKGEAGPQGARGSEGPQGVRGEPGPPGPAGAAGPAGNPGADGQPGAKGAN

GAPGIAGAPGFPGARGPSGPQGPSGAPGPKGNSGEPGAPGNKGDTGAKGEPGPAGVQGPP

GPAGEEGKRXXXGEPGPTGLPGPPGERGGPGSRGFPGSDGAAGPKGPAGERGSPGPAGPK

GSPGEAGRPGEAGLPGAKGLTGSPGSPGPDGKTGPTGPAGQDGRPGPpGPPGARGQAGVM

GFPGPKXXXXXXXXXAGERGVPGPPGAVGPAGKDGEAGAQGAPGPAGPAGERXXXXXXXXX

XXXXXXXXXXXXXXXXXXXXXXXXXXXXXXXXXXXXXXGFPGERGVQGPPGPAGPRGSN

GAPGNDGAKGDAGAPGAPGSQGAPGLQGMPGERGAAGLPGPKGDRXXXXXXXXXXXXXXD

GGRGLTGPIGPPGPAGAPGDKGESGPSGPAGPTGARGAPGDRGEPGPPGPAGFAGPPGAD

GQPGAKGEPGDAGAKGDAGPAGPAGPTGPPGPIGNVGAPGPKGARGSAGPPGATGFPGAA

GRVGPPGPSGNAGPPGPPGPVGKEGIKGPRGETGPAGRPGEVGPPGPPGPAGEKGSPGAD

GPAGSPGTPGPQGIAGQRGVVGLPGQRGERGFPGLPGPSGEPGKQGPSGSSGERGPPGPM

GPPGLAGPPGESGREGSPGAEGSPGRDGSPGQKGDRGETGPAGPPGAPGAPGAPGPVGPA

GKNGDRGETGPAGPAGPIGPAGARGPAGPQGPRGDKGETGEQGDRXXXGHRGFSGLQGPP

GPPGSPGEQGPSGASGPAGPRGPPGSAGAAGKDGLNGLPGPIGPPGPRGRXXXXXXXXXX

XXXXXXXXXXXXXXXXXXXXXXXXXXXXXXXXXXXXXXXXXXXGVGLGPGPMGLMGPRG

PPGASGAPGPqGFAGPAGEPGEPGQTGPAGARXXXXXXXXAGEDGHPGKPGRPGERXXXX

XXXXXGFPGTPGLPGFKXXXGHNGLDGLKGQPGAPGVKGEPGAPGENGTPGQTGARXXXX

XXXXVGAPGPAGARGSDGSVGPVGPAGPIGSAGPPGFPGAPGPKGELGGVGNPGPAGPAG

PRxxxxxxxxxxxxxxxxxxxxxxxxxxxGAAGLPGVAGAPGLPGPRGIPGPVGaaGASG

aRGLVGEPGPAGSKGETGNKGEPGSAGPQGPPGPSGEEGKRGSNGEPGSAGPTGPPGLRX

XXXXXGLPGADGRAGVMGPPGGRGASGPAGVRGPSGDSGRPGEPGLMGPRGFPGSPGSQG

PAGKEGPMGLPGIDGRPGPIGPAGARGEAGNIGFPGPKGPtGEPGKSGDKGHAGLAGARG

APGPDGNNGAQGPPGPQGVQGGKGEQGPAGPPGFQGLPGPAGTAGEAGKPGERGLNGEFG

LPGPAGPRGERGPPGqSGAAGpAGsiGsRGPSGpPGPDGNKGEPGVVGAPGNAGPSGPGG

LPGERGAAGIPGPKGDKGETGLRGEIGTTGRDGARGAPGAIGAPGPAGATGDRGEAGPSG

PAGPAGPRXXXXXXGEVGPAGPNGFAGPAGAAGQPGAKGERXXXGPKGEQGVVGPTGPVG

AAGPSGPNGPPGPAGTRGDGGPPGVTGFPGAAGRTGPPGPSGITGPPGPPGAAGKXXXXXG

PRGDQGPVGRTGETGASGLPGFAGEKGPAGEPGTAGPPGTPGPQGLLGAPGILGLPGSRG

ERGLPGVAGSLGEPGPLGISGPPGARGPPGAVGNPGVNGAPGEAGRDGNPGSDGPPGRXX

XXXXXXXXGYPGNIGPIGAAGAPGPHGSVGPAGKHGNRGETGPAGVVGPVGGFGPRGPSG

PQGIRGDKGEPGiKXXXXXXXXXXGHNGLQGLPGLAGHHGDQGSPGSVGPAGPRGPAGPIG

PAGKDGRSGHPGTVGPAGIRXXXXXXXXXXXXXXXXXXXXXXXXXXXXXXXXXXXXXXXX

>NesophontesHemicingulus

XXXXXXXXXXXXXxXXXXXXXXXXXXGLPGPAGAPGPQGFQGPPGEPGEPGASGPMGPR

GPPGPPGKNGDDGEAGKPGRPGERGPPGPQGARGLPGTAGLPGMKXXXGFSGLDGAKGDS

GPAGPKGEPGSPGENGAPGQMGPRXXXXXXGRPGAPGSAGARGNDGATGAAGPPGPTGPA

GPPGFPGAVGAKGEAGPQGARGSEGPQGVRGEPGPPGPAGAAGPAGNPGADGQPGAKGAN

GAPGIAGAPGFPGARGPSGPQGPSGAPGPKGNSGEPGAPGNKGDTGAKGEPGPAGVQGPP

GPAGEEGKRXXXGEPGPTGLPGPPGERGGPGSRGFPGSDGAAGPKGPAGERGSPGPAGPK

GSPGEAGRPGEAGLPGAKGLTGSPGSPGPDGKTGPTGPAGQDGRPGPpGPPGARGQAGVM

GFPGPKXXXXXXXXXAGERGVPGPPGAVGPAGKDGEAGAQGAPGPAGPAGERGEQGPAGSP

GFQGLPGPAGPPGEAGKXXXXXXXXXXXXXXXXXXXXXXXGFPGERGVQGPPGPAGPRGSN

GAPGNDGAKGDAGAPGAPGSQGAPGLQGMPGERGAAGLPGPKGDRXXXXXXXXXXXXXXD

GGRGLTGPIGPPGPAGAPGDKGESGPSGPAGPTGARGAPGDRGEPGPPGPAGFAGPPGAD

GQPGAKGEPGDAGAKGDAGPAGPAGPTGPPGPIGNVGAPGPKGARGGAGPPGATGFPGAA

GRVGPPGPSGNAGPPGPPGPVGKEGIKGPRGETGPAGRPGEVGPPGPPGPAGEKGSPGAD

GPAGSPGTPGPQGIAGQRGVVGLPGQRGERGFPGLPGPSGEPGKQGPSGSSGERGPPGPM

GPPGLAGPPGESGREGSPGAEGSPGRDGSPGQKGDRGETGPAGPPGAPGAPGAPGPVGPA

GKNGDRGETGPAGPAGPIGPAGARGPAGPQGPRGDKGETGEQGDRXXXXXXGFSGLQGPP

GPPGSPGEQGPSGASGPAGPRGPPGSAGAAGKDGLNGLPGPIGPPGPRGRXXXXXXXXXX

XXXXXXXXXXXXXXXXXXXXXXXXXXXXXXXXXXXXXXXXXXXXXGVGLPGPMGLMGPRX

XXXXXXXXXXXXXXXXXXXXXXXXXXXXXXXXXXXXXXXXAGEDGHPGKPGRPGERXXXX

XXXXXGFPGTPGLPGFKXXXGHNGLDGLKXXXXXXXXXGEPGAPGENGTPGQTGARXXXX

XXXXVGAPGPAGARGSDGSVGPVGPAGPIGSAGPPGFPGAPGPKGELGGVGNPGPAGPAG

PRXXXXXXXXXXXXXXXXXXXXXXXXXXXGAAGLPGVAGAPGLPGPRGIPGPVGaaGASG

aRGLVGEPGPAGSKGETGNKGEPGSAGPQGPPGPSGEEGKRGSNGEPGSAGPTGPPGLRX

XXXXXXXXXXXXXAGVMGPPGGRGASGPAGVRGPSGDSGRPGEPGLMGPRGFPGSPGSQG

PAGKEGPMGLPGIDGRPGPIGPAGARGEAGNIGFPGPKXXXXXXXXSGDKGHAGLAGARG

APGPDGNNGAQGPPGPQGVQGGKGEQGPAGPPGFQGLPGPAGTAGEAGKPGERGLNGEFG

LPGPAGPRGERGPPGqSGAAGpAGsiGsRGPSGpPGPDGNKGEPGVVGAPGNAGPSGPGG

LPGERGAAGIPGPKXXXXXXXXXGEIGTTGRXXXXGAPGAIGAPGPAGATGDRGEAGPSG

PAGPAGPRXXXXXXGEVGPAGPNGFAGPAGAAGQPGAKGERXXXGPKGEQGVVGPTGPVG

AAGPSGPNGPPGPAGTRGDGGPPGVTGFPGAAGRTGPPGPSGITGPPGPPGAAGKXXXXG

PRGDQGPVGRTGETGASGLPGFAGEKGPAGEPGTAGPPGTPGPQGLLGAPGILGLPGSRG

ERGLPGVAGSLGEPGPLGISGPPGARGPPGAVGNPGVNGAPGEAGRDGNPGSDGPPGRXX

XXXXXXXXGYPGNIGPIGAAGAPGPHGSVGPAGKHGNRGETGPAGVVGPVGGFGPRGPSG

PQGIRXXXXXXXXXXXXXXXXXXXXXXXXXXXXXXXXXXXXXXXXXXXXXXXXGPAGPIG

PAGKDGRSGHPGTVGPAGIRXXXXXXXXXXXXXXXXXXXXXXXXXXXXXXXXXXXXXXXX

>NesophontesMicrus

XXXXXXXXXSSGG-MSVPGPMGPSGPRGLPGPAGAPGPQGFQGPPGEPGEPGASGPMGPR

GPPGPPGKNGDDGEAGKPGRPGERGPPGPQGARGLPGTAGLPGMKGHRGFSGLDGAKGDS

GPAGPKGEPGSPGENGAPGQMGPRXXXXXXGRPGAPGSAGARGNDGATGAAGPPGPTGPA

GPPGFPGAVGAKGEAGPQGARGSEGPQGVRGEPGPPGPAGAAGPAGNPGADGQPGAKGAN

GAPGIAGAPGFPGARGPSGPQGPSGAPGPKGNSGEPGAPGNKGDTGAKGEPGPAGVQGPP

GPAGEEGKRGARGEPGPTGLSGPPGERGGPGSRGFPGSDGAAGPKGPAGERGSPGPAGPK

GSPGEAGRPGEAGLPGAKGLTGSPGSPGPDGKTGPTGPAGQDGRPGPpGPPGARGQAGVM

GFPGPKXXXXXXXXXAGERGVPGPPGAVGPAGKDGEAGAQGAPGPAGPAGERXXXXXXXXX

XXXXXXXXXXXXXXXXXXXXXXXXXXXXXXXXXXXXXGFPGERGVQGPPGPAGPRGSN

GAPGNDGAKGDAGAPGAPGSQGAPGLQGMPGERGAAGLPGPKGDRXXXXXXXXXXXXXXD

GGRGLTGPIGPPGPAGAPGDKGESGPSGPAGPTGARGAPGDRGEPGPPGPAGFAGPPGAD

GQPGAKGEPGDAGAKGDAGPAGPAGPTGPPGPIGNVGAPGPKGARGSAGPPGATGFPGAA

GRVGPPGPSGNAGPPGPPGPVGKXXXXXXXGETGPAGRPGEVGPPGPPGPTGEKGSPGAD

GPAGSPGTPGPQGIAGQRGVVGLPGQRGERGFPGLPGPSGEPGKQGPSGSSGERGPPGPM

GPPGLAGPPGESGREGSPGAEGSPGRDGSPGQKGDRGETGPAGPPGAPGAPGAPGPVGPA

GKNGDRGETGPAGPAGPIGPAGARGPAGPQGPRGDKGETGEQGDRXXXXXXGFSGLQGPP

GPPGSPGEQGPSGASGPAGPRGPPGSAGAAGKDGLNGLPGPIGPPGPRGRXXXXXXXXXX

XXXXXXXXXXXXXXXXXXXXXXXXXXXXXXXXXXXXXXXXXGVGLPGPMGLMGPRG

PPGASGAPGPqGFaGPAGEPGEPGQTGPAGARXXXXXXXXAGEDGHPGKPGRPGERXXXX

XXXXXGFPGTPGLPGFKXXXXXXXXXXXXXXXXXXXXGEPGAPGENGTPGQTGARXXXX

XXGRVGAPGPAGARGSDGSVGPVGPAGPIGSAGPPGFPGAPGPKGELGGVGNPGPAGPAG

PRxxxxxxxxxxxxxxxxxxxxxxxxxxGAAGLPGVAGAPGLPGPRGIPGPVGaaGASG

aRGLVGEPGPAGSKGETGNKGEPGSAGPQGPPGPSGEEGKRGSNGEPGSTGPTGPPGLRx

xxxxxGLPGADGRAGVMGPPGGRGASGPAGVRXXXXXXXXPGEPGLMGPRGFPGSPGSQG

PAGKEGPMGLPGIDGRPGPIGPAGARGEAGNIGFPGPKGPtGEPGKSGDKGHAGLAGARG

APGPDGNNGAQGPPGPQGVQGGKGEQGPAGPPGFQGLPGPAGTAGEAGKPGERGLNGEFG

LPGPAGPRGERGPPGqSGAAGpAGsiGNRGPSGPPGPDGNKGEPGVVGAPGNAGPSGPGG

LPGERGAAGIPGPKGDKGETGLRXXXXXXXXXXXXGAPGAIGAPGPAGATGDRGEAGPSG

PAGPAGPRXXXXXXGEVGPAGPNGFAGPAGAAGQPGAKGERXXXGPKGEQGVVGPTGPVG

AAGPSGPNGPPGPAGTRGDGGPPGVTGFPGAAGRTGPPGPSGITGPPGPPGAAGKEGLRG

PRGDQGPVGRTGETGASGLPGFAGEKGPAGEPGTAGPPGTPGPQGLLGAPGILGLPGSRG

ERGLPGVAGSLGEPGPLGISGPPGARGPPGAVGNPGVNGAPGEAGRDGNPGSDGPPGRXX

XXXXXXXXGYPGNIGPiGAAGAPGPHGSVGPAGKHGNRGETGPAGVVGPVGGFGPRGPSG

PQGIRGDKGEPGiKXXXXXXXXXXXXXXXXXXXXXXXXXXXXXXXXXXXXXXXXXGPAGPIG

PAGKDGRSGHPGTVGPAGIRXXXXXXXXXXXXXXXXXXXXXXXXXXXXXXXXXXXXXXXXX

>NesophontesZamicrus

XXXXXXXXXSSGG-MSVPGPMGPSGPRGLPGPPGAPGPQGFQGPPGEPGEPGASGPMGPR

GPPGPPGKNGDDGEAGKPGRPGERGPPGPQGARGLPGTAGLPGMKXXXGFSGLDGAKGDS

GPAGPKGEPGSPGENGAPGQMGPRXXXXXXGRPGAPGSAGARGNDGATGAAGPPGPTGPA

GPPGFPGAVGAKGEAGPQGARXXXXXXXXXGEPGPPGPAGAAGPAGNPGADGQPGAKGAN

GAPGIAGAPGFPGARGPSGPQGPSGAPGPKGNSGEPGAPGNKGDTGAKGEPGPAGVQGPP

GPAGEEGKRXXXGEPGPTGLSGPPGERXXXXXXGFPGSDGAAGPKXXXXXXGSPGPAGPK

GSPGEAGRPGEAGLPGAKGLTGSPGSPGPDGKTGPTGPAGQDGRPGPpGPPGARGQAGVM

GFPGPKXXXXXXXXXAGERGVPGPPGAIGPAGKDGEAGAQGAPGPAGPAGERGEQGPAGSP

GFQGLPGPAGPPGEAGKXXXXXXXXXXXXXXXXXXXXXXXXXXXXGVQGPPGPAGPRGSN

GAPGNDGAKGDAGAPGAPGSQGAPGLQGMPGERGAAGLPGPKGDRXXXXXXXXXXXXXXX

XXXGLTGPIGPPGPAGAPGDKGESGPSGPAGPTGARGAPGDRGEPGPPGPAGFAGPPGAD

GQPGAKGEPGDAGAKGDAGPAGPAGPTGPPGPIGNVGAPGPKGARGSAGPPGATGFPGAA

GRVGPPGPSGNAGPPGPPGPVGKXXXXXXXGETGPAGRPGEVGPPGPPGPAGEKGSPGAD

GPAGSPGTPGPQGIAGQRGVVGLPGQRXXXGFPGLPGPSGEPGKQGPSGSSGERGPPGPM

GPPGLAGPPGESGRXXXXXXXXXXXXDGSPGQKGDRGETGPAGPPGAPGAPGAPGPVGPA

GKNGDRGETGPAGPAGPIGPAGARGPAGPQGPRXXXXXXXXXXXXXXXXXXGFSGLQGPP

GPPGSPGEQGPSGASGPAGPRGPPGSAGAAGKDGLNGLPGPIGPPGPRXXXXXXXXXXXX

XXXXXXXXXXXXXXXXXXXXXXXXXXXXXXXXXXXXXXXXXXXXGVGLGPGPMGLMGPRX

XXXXXXXXXXXXXXXXXXXXXXXXXXXXXXXXXXXXXXXXAGEDGHPGKPGRPGERXXXX

XXXXXGFPGTPGLPGFKXXXGHNGLDGLKGQPGAPGVKGEPGAPGENGTPGQTGARXXXX

XXXXVGAPGPAGARGSDGSVGPVGPAGPIGSAGPPGFPGAPGPKGELGGVGNPGPAGPAG

PRXXXXXXXXXXXXXXXXXXXXXXXXXXXXGAAGLPGVAGAPGLPGPRGIPGPVGaaGASG

aRGLVGEPGPAGSKGETGNKGEPGSAGPQGPPGPSGEEGKRGSNGEPGSAGPTGPPGLRX

XXXXXXXXXXXXXAGVMGPPGGRGASGPAGVRXXXXXXXXPGEPGLMGPRGFPGSPGSQG

PAGKEGPMGLPGIDGRPGPIGPAGARGEAGNIGFPGPKGPtGEPGKSGDKGHAGLAGARG

APGPDGNNGAQGPPGPQGVQGGKGEQGPAGPPGFQGLPGPAGTAGEAGKPGERGLNGEFG

LPGPAGPRGERGPPGqSGAAGpAGsiGsRGPSGPPGPDGNKGEPGVVGAPGNAGPSGPGG

LPGERGAAGIPGPKGDKGETGLRGEIGTTGRXXXXGAPGAIGAPGPAGATGDRGEAGPSG

PAGPAGPRXXXXXXGEVGPAGPNGFAGPAGAAGQPGAKGERXXXGPKGEQGVVGPTGPVG

AAGPSGPNGPPGPAGTRGDGGPPGVTGFPGAAGRTGPPGPSGItGPPGPPGAAGKXXXXXX

XXGDQGPVGRTGETGASGLPGFAGEKGPAGEPGTAGPPGTPGPQGLLGAPGILGLPGSRG

ERGLPGVAGSLGEPGPLGISGPPGARGPPGAVGNPGVNGAPGEAGRDGNPGSDGPPGRXX

XXXXXXXXGYPGNIGPIGAAGAPGPHGSVGPAGKXXXXGETGPAGaVGPVGGFGPRGPSG

PQGIRGDKGEPGiKGARXXXXXXGHNGLQGLPGLAGHHGDQGSPGSVGPAGPRGPAGPIG

PAGKDGRSGHPGTVGPAGIRXXXXXXXXXXXXXXXXXXXXXXXXXXXXXXXXXXXXXXXX

>NesophontesHypomicrus

XXXXXXXXXSSGG-MSVPGPMGPSGPRGLPGPPGAPGPQGFQGPPGEPGEPGASGPMGPR

GPPGPPGKNGDDGEAGKPGRXXXXGPPGPQGARGLPGTAGLPGMKXXXGFSGLDGAKXXX

XXXXXXGEPGSPGENGAPGQMGPRXXXXXXGRPGAPGSAGARGNDGATGAAGPPGPTGPA

GPPGFPGAVGAKGEAGPQGARGSEGPQGVRGEPGPPGPAGAAGPAGNPGADGQPGAKGAN

GAPGIAGAPGFPGARGPSGPQGPSGAPGPKGNSGEPGAPGNKGDTGAKGEPGPAGVQGPP

GPAGEEGKRGARGEPGPTGLSGPPGERXXXXXXGFPGSDGAAGPKGPAGERGSPGPAGPK

GSPGEAGRPGEAGLPGAKGLTGSPGSPGPDGKTGPTGPAGQDGRPGPpGPPGARGQAGVM

GFPGPKXXXXXXXXXAGERGVPGPPGAIGPAGKDGEAGAQGAPGPAGPAGERXXXXXXXXX

XXXXXXXXXXXXXXXXXXXXXXXXXXXXXXXXXXXXXXXXXXXXXGVQGPPGPAGPRGSN

GAPGNDGAKGDAGAPGAPGSQGAPGLQGMPGERGAAGLPGPKGDRXXXXXXXXXXXXXXX

XXXGLTGPIGPPGPAGAPGDKGESGPSGPAGPTGARGAPGDRGEPGPPGPAGFAGPPGAD

GQPGAKGEPGDAGAKGDAGPAGPAGPTGPPGPIGNVGAPGPKGARGSAGPPGATGFPGAA

GRVGPPGPSGNAGPPGPPGPVGKEGIKGPRGETGPAGRPGEVGPPGPPGPAGEKGSPGAD

GPAGSPGTPGPQGIAGQRGVVGLPGQRXXXGFPGLPGPSGEPGKXXXXXXXXXXGPPGPM

GPPGLAGPPGESGRXXXXXXXXXXXXXXXXXXXGDRGETGPAGPPGAPGAPGAPGPVGPA

GKNGDRGETGPAGPAGPIGPAGARGPAGPQGPRXXXXXXXXXXXXXXXXXXGFSGLQGPP

GPPGSPGEQGPSGASGPAGPRGPPGSAGAAGKDGLNGLPGPIGPPGPRXXXXXXXXXXXX

XXXXXXXXXXXXXXXXXXXXXXXXXXXXXXXXXXXXXXXXXXXXGVGLGPGPMGLMGPRG

PPGASGAPGPQGFaGPAGEPGEPGQTGPAGaRXXXXXXXXXXXXXXXXXXXXXXXXXXXX

XXXXXGFPGTPGLPGFKXXXGHNGLDGLKXXXXXXXXXXGEPGAPGENGTPGQTGARXXXX

XXXXVGAPGPAGARGSDGSVGPVGPAGPIGSAGPPGFPGAPGPKGELGGVGNPGPAGPAG

PRXXXXXXXXXXXXXXXXXXXXXXXXXXXGAAGLPGVAGAPGLPGPRGIPGPVGaaGASG

aRGLVGEPGPAGSKGETGNKGEPGSAGPQGPPGPSGEEGKRGSNGEPGSAGPTGPPGLRX

XXXXXGLPGADGRAGVMGPPGgRGASGPAGVRXXXXXXXXXPGEPGLMGPRGFPGSPGSQG

PAGKEGPMGLPGIDGRPGPIGPAGARGEAGNIGFPGPKXXXXXXXXXSGDKGHAGLAGARG

APGPDGNNGAQGPPGPQGVQGGKGEQGPAGPPGFQGLPGPAGTAGEAGKPGERGLNGEFG

LPGPAGPRGERGPPGqSGAAGpAGsiGsRGPSGPPGPDGNKGEPGVVGAPGNAGPSGPGG

LPGERGAAGIPGPKXXXXXXXXXXGEIGTTGRXXXXGAPGAIGAPGPAGATGDRGEAGPSG

PAGPAGPRXXXXXXGEVGPAGPNGFAGPAGAAGQPGAKGERXXXGPKGEQGVVGPTGPVG

AAGPSGPNGPPGPAGTRGDGGPPGVTGFPGAAGRTGPPGPSGITGPPGPPGAAGKXXXXG

PRGDQGPVGRTGETGASGLPGFAGEKGPAGEPGTAGPPGTPGPQGLLGAPGILGLPGSRG

ERGLPGVAGSLGEPGPLGISGPPGARGPPGAVGNPGVNGAPGEAGRDGNPGSDGPPGRXX

XXXXXXXXGYPGNIGPIGAAGAPGPHGSVGPAGKHGNRGETGPAGaVGPVGGFGPRGPSG

PQGIRXXXGEPGiKGARGLPGLKXXXXXXXXXXXXXXXXXXXXXXXXXXXXXXGPAGPIG

PAGKDGRSGHPGTVGPAGIRXXXXXXXXXXXXXXXXXXXXXXXXXXXXXXXXXXXXXXXX

>NesophontesParamicrus

XXXXXXXXXSSGG-MSVPGPMGPSGPRGLPGPPGAPGPQGFQGPPGEPGEPGASGPMGPR

XXXXXXXXNGDDGEAGKPGRPGERGPPGPQGARGLPGTAGLPGMKGHRGFSGLDGAKGDS

GPAGPKGEPGSPGENGAPGQMGPRXXXXXXGRPGAPGSAGARGNDGATGAAGPPGPTGPA

GPPGFPGAVGAKGEAGPQGARGSEGPQGVRGEPGPPGPAGAAGPAGNPGADGQPGAKGAN

GAPGIAGAPGFPGARGPSGPQGPSGAPGPKGNSGEPGAPGNKGDTGAKGEPGPAGVQGPP

GPAGEEGKRXXXGEPGPTGLSGPPGERXXXXXXGFPGSDGAAGPKXXXXXXXGSPGPAGPK

GSPGEAGRPGEAGLPGAKGLTGSPGSPGPDGKTGPTGPAGQDGRPGPpGPPGARGQAGVM

GFPGPKXXXXXXXXXAGERGVPGPPGAIGPAGKDGEAGAQGAPGPAGPAGERGEQGPAGSP

GFQGLPGPAGPPGEAGKXXXXXXXXXXXXXXXXXXXXXXGFPGERGVQGPPGPGGPRGSN

GAPGNDGAKGDAGAPGAPGSQGAPGLQGMPGERXXXXXXXXXXXXXXXXXXXXXXXXXXD

GGRGLTGPIGPPGPAGAPGDKGESGPSGPAGPTGARGAPGDRGEPGPPGPAGFAGPPGAD

GQPGAKGEPGDAGAKGDAGPAGPAGPTGPPGPIGNVGAPGPKGARGSAGPPGATGFPGAA

GRVGPPGPSGGAGPPGPPGPVGKXXXXXXXGETGPAGRPGEVGPPGPPGPAGEKGSPGAD

GPAGSPGTPGPQGIAGQRGVVGLPGQRGERGFPGLPGPSGEPGKQGPSGSSGERGPPGPM

GPPGLAGPPGESGREGSPGAEGSPGRXXXXXXXGDRGETGPAGPPGAPGAPGAPGPVGPA

GKNGDRGETGPAGPAGPIGPAGARGPAGPQGPRGDKGETGEQGDRXXXXXXGFSGLQGPP

GPPGSPGEQGPSGASGPAGPRGPPGSAGAAGKDGLNGLPGPIGPPGPRGRXXXXXXXXXX

XXXXXXXXXXXXXXXXXXXXXXXXXXXXXXXXXXXXXXXXXXXXGVGLGPGPMGLMGPRG

PPGASGAPGPQGFAGPAGEPGEPGQTGPAGaRXXXXXXXXAGEDGHPGKPGRPGERXXXX

XXXXXGFPGTPGLPGFKXXXXXXXXXXXXGQPGAPGVKGEPGAPGENGTPGQTGARXXXX

XXXXVGAPGPAGARGSDGSVGPVGPAGPIGSAGPPGFPGAPGPKGELGGVGNPGPAGPAG

PRXXXXXXXXXXXXXXXXXXXXXXXXXXXGAAGLPGVAGAPGLPGPRGIPGPVGaaGASG

PRGLVGEPGPAGSKGETGNKGEPGSAGPQGPPGPSGEEGKRGSNGEPGSAGPTGPPGLRX

XXXXXXXXXXXXXxxxxxxxxxxGASGPAGVRXXXXXXXXPGEPGLMGPRGFPGSPGSQG

PAGKEGPMGLPGIDGRPGPIGPAGARGEAGNIGFPGPKGPtGEPGKSGDKGHAGLAGARG

APGPDGNNGAQGPPGPQGVQGGKGEQGPAGPPGFQGLPGPAGTAGEAGKPGERGLNGEFG

LPGPAGPRGERGPPGqSGAAGpAGsiGsRGPSGPPGPDGNKGEPGVVGAPGNAGPSGPGG

LPGERGAAGIPGPKGDKGETGLRGEIGTTGRXXXXGAPGAIGAPGPAGATGDRGEAGSSG

PAGPAGPRXXXXXXGEVGPAGPNGFAGPAGAAGQPGAKGERXXXGPKGEQGVVGPTGPVG

AAGPSGPNGPPGPAGTRGDGGPPGVTGFPGAAGRTGPPGPSGITGPPGPPGAAGKXXXXX

XXGDQGPVGRTGETGASGLPGFAGEKGPAGEPGTAGPPGTPGPQGLLGAPGILGLPGSRG

ERGLPGVAGSLGEPGPLGISGPPGARGPPGAVGNPGVNGAPGEAGRDGNPGSDGPPGRXX

XXXXXXXXGYPGNIGPIGAAGAPGPHGSVGPAGKXXXXGETGPAGaVGPVGGFGPRGPSG

PQGIRGDKGEPGiKGARGLPGLKXXXXXXXXXXXXXXXXXXXXXXXXXXXXXXGPAGPIG

PAGKXXXSGHPGTVGPAGIRXXXXXXXXXXXXXXXXXXXXXXXXXXXXXXXXXXXXXXX

>NesophontesEdithae

XXXXXXXXXXXXXxXXXXXXXXXXXXXGLPGPPGAPGPQGFQGPPGEPGEPGASGPMGPR

GPPGPPGKNGDDGEAGKPGRPGERGPPGPQGARGLPGTAGLPGMKXXXGFSGLDGAKGDS

GPAGPKGEPGSPGENGAPGQMGPRXXXXXXGRPGAPGSAGARGNDGATGAAGPPGPTGPA

GPPGFPGAVGAKGEAGPQGARGSEGPQGVRGEPGPPGPAGAAGPAGNPGADGQPGAKGAN

GAPGIAGAPGFPGARGPSGPQGPSGAPGPKGNSGEPGAPGNKGDTGAKGEPGPAGVQGPP

GPAGEEGKRXXXGEPGPTGLsGPPGERGGPGSRGFPGADGAAGPKGPAGERGSPGPAGPK

GSPGEAGRPGEAGLPGAKGLTGSPGSPGPDGKTGPTGPAGQDGRPGPPGPPGARGQAGVM

GFPGPKGAAGEPGKXXXXGVPGPPGAVGPAGKDGEAGAQGAPGPAGPAGERXXXXXXXXX

XXXXXXXXXXXXXXXXXXXXXXXXXXXXXXXXXXXXXXXXXXXXXGVQGPPGPAGPRGSN

GAPGNDGAKGDAGAPGAPGSQGAPGLQGMPGERGAAGLPGPKGDRXXXXXXXXXXXXXXX

XXXGLTGPIGPPGPAGAPGDKGESGPSGPGGPTGARGAPGDRGEPGPPGPAGFAGPPGAD

GQPGAKGEPGDAGAKGDAGPAGPAGPTGPPGPIGNVGAPGPKXXXGSAGPPGATGFPGAA

GRVGPPGPSGNAGPPGPPGPVGKXXXXXXXGETGPAGRPGEVGPPGPPGPAGEKGSPGAD

GPAGSPGTPGPQGIAGQRGVVGLPGQRXXXGFPGLPGPSGEPGKXXXXXXXXXXGPPGPM

GPPGLAGPPGESGRXXXXXXXXXXXXXXXXXXXGDRGETGPAGPPGAPGTPGAPGPVGPA

GKNGDRGETGPAGPAGPIGPAGARXXXXXXXXXXXXXXXXXXXXXXXXXXXXGFSGLQGPP

GPPGSPGEQGPSGASGPAGPRGPPGSAGAAGKDGLNGLPGPIGPPGPRXXXXXXXXXXXX

XXXXXXXXXXXXXXXXXXXXXXXXXXXXXXXXXXXXXXXXXXXXXGVGLGPGPMGLMGPRG

PPGASGAPGPQGFAGPAGEPGEPGQTGPAGARXXXXXXXXAGEDGHPGKPGRXXXXXXXX

XXXXXGFPGTPGLPGFKXXXXXXXXXXXXXXXXXXXXXGEPGAPGENGTPGQTGARXXXX

XXXXVGAPGPAGARGSDGSVGPVGPAGPIGSAGPPGFPGAPGPKGELGGVGNPGPAGPAG

PRXXXXXXXXXXXXXXXXXXXXXXXXXXXGAAGLPGVAGAPGLPGPRGIPGPVGaaGASG

aRGLVGEPGPAGSKGETGNKGEPGSAGPQGPPGPSGEEGKRGSNGEPGSSGPTGPPGLRX

XXXXXGLPGADGRAGVMGPPGGRGASGPAGVRGPSGDSGRPGEPGLMGPRGFPGSPGSQG

PAGKEGPMGLPGIDGRPGPIGPAGARGEAGNIGFPGPKxxxxxxxxSGDKGHAGLAGARG

APGPDGNNGAQGPPGPQGVQGGKGEQGPAGPPGFQGLPGPAGTAGEAGKPGERGLNGEFG

LPGPAGPRGERGPPGqSGAAGpAGsiGsRGPSGPPGPDGNKGEPGVVGAPGNAGPSGPGG

LPGERGAAGIPGPKGDKGETGLRXXXXXXXXXXXXGAPGAIGAPGPAGATGDRGEAGPSG

PAGPAGPRXXXXXXGEVGPAGPNGFAGPAGAAGQPGAKGERXXXGPKGEQGVVGPTGPVG

AAGPSGPNGPPGPAGTRGDGGPPGVTGFPGAAGRTGPPGPSGITGPPGPPGAAGKXXXXX

XXGDQGPVGRTGETGASGLPGFAGEKXXXXXXXXXXXXXXXXXXXXXXXXXXXXXXXXXX

XXGLPGVAGSLGEPGPLGISGPPGARGPPGAVGNPGVNGAPGEAGRDGNPGSDGPPGRXX

XXXXXXXXXXXXXXXXXXXXXXXXXXXXXXXXXXXXXXGETGPAGAVGPVGGFGPRGPSG

PQGIRXXXXXXXXXXXXXXXXXXXXXXXXXXXXXXXXXXXXXXXXXXXXXXXGPAGPIG

PAGKDGRSGHPGTVGPAGIRXXXXXXXXXXXXXXXXXXXXXXXXXXXXXXXXXXXXXX

>SolenodonParadoxus

QMSYGYDEKSAGG-MSVPGPMXXXXXXXXXXXXXXXXXXXXXXXXXXXXXXXXXXXXXX

GPPGPPGKXXXXXXXXXXXXXXXXGPPGPQGARGLPGTAGLPGMKXXXGFSGLDGAKGDS

GPAGPKGEPGSPGENGAPGQMGPRXXXXXXXXXXXXXXXXXXGNDGATGAAGPPGPTGPA

GPPGFPGAVGAKGEAGPQGSRXXXXXXXXXGEPGPPGPAGAAGPAGNPGADGQPGAKGAN

GAPGIAGAPGFPGARGPSGPQGPSGAPGPKXXXXXXXXXXXXXXXXXXGEPGPAGVQGPP

GPAGEEGKXXXXGEPGPTGLPGPPGERXXXXXXGFPGSDGAAGPKXXXXXXGIPGPAGPK

GSPGEAGRPGEAGLPGAKGLTGSPGSPGPDGKTGPPGPAGQDGRPGPPGPPGARGQAGVM

GFPGPKGAAGEPGKXXXXGVPGPPGAIGAAGKDGEAGAQGAPGPAGPAGERGEQGPAGSP

GFQGLPGPAGPPGEAGKPGEQGAPGDLGAPGPSGARXXXXXXXXXGVQGPPGPAGPRGAN

GAPGNDGAKGDAGAPGAPGSQGAPGLQGMPGERXXXXXXXXXXXXXXXXXXXXXXXXXXD

GGRGLTGPIGPPGPAGAPGDKGESGPSGPAGPTGARGAPGDRGEPGPPGPAGFAGPPGAD

GQPGAKGEPGDAGAKGDAGPPGPAGPTGAPGPIGNVGAPGPKGARGSAGPPGATGFPGAA

GRVGPPGPSGNAGPPGPPGPVGKXXXXXXXGETGPAGRPGEVGPPGPPGPTGEKGSPGAD

GPAGSPGTPGPQGIAGQRGVVGLPGQRGERGFPGLPGPSGEPGKQGPSGSSGERGPPGPM

GPPGLAGPPGESGREGSPGAEGSPGRDGSPGQKGDRGETGPAGPPGAPGAPGAPGPVGPA

GKNGDRGETGPAGPAGPAGPAGARGPAGPQGPRGDKGEAGEQGDRXXXXXXGFSGLQGPP

GPPGSPGEQGPSGASGPAGPRGPPGSAGAAGKDGLNGLPGPIGPPGPRXXXXXXXXXXXX

XXXXXXXXXXXXXXXXXXXXXXXXXXXXXXXXXXXXXXXXXXXXGVGLGPGPMGLMGPRG

PPGASGAPGPQGFQGPAGEPGEPGQTGPAGARXXXXXXXXXXXXXXXXXXXXXXXXXXXX

XXXXXGFPGTPGLPGFKXXXGHNGLDGLKXXXXXXXXXXGEPGAPGENGTPGQAGARXXXX

XXXXXXXXXXXXXXGSDGSVGPVGPAGPIGSAGPPGFPGAPGPKGELGGVGNPGPAGPAG

PRXXXXXXXXXXXXXXXXXXXXXXXXXXXGAAGLPGVAGAPGLPGPRGIPGPAGAaGAsG

ARGLVGEPGPAGSKGEsGNKGEPGSAGAQGPPGPSGEEGKRGQNGEAGSAGPTGPPGLRX

XXXXXXXXXXXXXAGVMGLAGSRGASGPAGARXXXXXXXXPGEPGLMGPRGFPGSPGSqG

PAGKEGPMGLPGIDGRPGPIGPAGARGEAGNIGFPGPKXXXXXXXXXXXXXGHAGLAGARG

APGPDGNNGAQGPPGPQGVQGGKGEQGPAGPPGFQGLPGPAGTTGEVGKXXXXGLPGEFG

LPGPAGPRXXXGPPGqSGAAGpAGsiGNRGPSGPPGPDGNKGEPGVVGAPGNAGASGPGG

LPGERGAAGIPGPKXXXGETGLRGEIGTTGRDGARGAPGAVGAPGPAGATGDRGEAGAAG

PAGPAGPRGSPGERGEVGAAGPNGFAGPAGAAGQPGAKGERGTKGPKGENGGVGPTGPVG

SAGPSGPNGPPGPAGSRGDGGPPGMTGFPGAAGRTGSPGPSGITGPPGPTGAAGKXXXXX

XXGDQGPVGRTGETGASGLPGFPGEKGPAGEPGTAGPPGTAGPQGLLGAPGILGLPGSRX

XXGLPGVSGSLGEPGPLGISGPPGARGPPGAVGNPGVNGAPGEAGRDGNPGSDGPPGRXX

XXXXXXXXXXXXXXXXXXXXXXXXXXXXXXXXXXXXXXGEPGPAGvVGPvGAfGPRGPSG

VQGARGDKGEAGEKGPRGLPGLKGHNGLQGLPGLAGHHGDQGAPGSVGPAGPRGPAGPSG

PVGKDGRTGHPGTVGPAGIRGSQGNQGPAGPPGPPGPPGPPGVSGGGYDFGYDGDFYRA

>SolenodonCubanus

XXXXXXXXXSAGG-MSVPGPMGPSGPRXXXXXXXXXXXXXXXXXXXXXXXXXXXXXXXX

GPPGPPGKNGDDGEAGKPGRPGERGPPGPQGARGLPGTAGLPGMKGHRGFSGLDGAKGDS

GPAGPKGEPGSPGENGAPGQMGPRXXXXXXGRPGPPGSAGARGNDGATGAAGPPGPTGPA

GPPGFPGAVGAKGEAGPQGSRGSEGPQGVRGEPGPPGPAGAAGPAGNPGADGQPGAKGAN

GAPGIAGAPGFPGARxxxxxxxxxxxxxxxGNSGEPGAPGNKXXXXXXGEPGPAGVQGPP

GPAGEEGKRXXXGEPGPTGLPGPPGERGGPGSRGFPGSDGAAGPKXXXXXXGSPGPAGPK

GSPGEAGRPGEAGLPGAKGLTGSPGSPGPDGKTGPPGPAGQDGRPGPpGPPGARGQAGVM

GFPGPKGAAGEPGKXXXXGVPGPPGAIGAAGKDGEAGAQGAPGPAGPAGERXXXXXXXXX

XXXXXXXXXXXXXXXXXXXXXXXXXXXXXXXXXXXXXXXXXXXXXXXXXXGVQGPPGPAGPRGAN

GAPGNDGAKGDAGAPGAPGSQGAPGLQGMPGERGAAGLPGPKGDRGDAGPKXXXXXXXXXD

GGRGLTGPIGPPGPAGAPGDKGESGPSGPAGPTGARGAPGDRGEPGPPGPAGFAGPPGAD

GQPGAKGEPGDAGAKGDAGPPGPAGPTGAPGPIGNVGAPGPKGARGSAGPPGATGFPGAA

GRVGPPGPSGNAGPPGPPGPVGKXXXXXXXGETGPAGRPGEVGPPGPPGPTGEKGSPGAD

GPAGSPGTPGPQGIAGQRGVVGLPGQRGERGFPGLPGPSGEPGKQGPSGSSGERGPPGPM

GPPGLAGPPGESGREGSPGAEGSPGRDGSPGQKGDRGETGPAGPPGAPGAPGAPGPVGPA

GKNGDRGETGPAGPAGPAGPAGARGPAGPQGPRGDKGEAGEQGDRXXXGHRGFSGLQGPP

GPPGSPGEQGPSGASGPAGPRGPPGSAGAAGKDGLNGLPGPIGPPGPRXXXXXXXXXXXX

XXXXXXXXXXXXXXXXXXXXXXXXXXXXXXXXXXXXXXXXXXXXXXXXXGVGLGPGPMGLMGPRG

PPGASGAPGPQGFQGPAGEPGEPGQTGPAGARXXXXXXXXXXXXXXXXXXXXXXXXXXXGVMG

PQGARGFPGTPGLPGFKXXXGHNGLDGLKGQPGAPGVKGEPGAPGENGTPGQAGARXXXX

XXXXxxxxxxxxxxGSDGSVGPVGPAGPIGSAGPPGFPGAPGPKGELGGVGNPGPAGPAG

PRXXXXXXXXXXXXXXXXXXXXXXXXXXXGAAGLPGVAGAPGLPGPRGIPGPAGAAGASG

ARGLVGEPGPAGSKGEGGNKGEPGSAGAQGPPGPSGEEGKRGQNGEAGSAGPTGPTGLRX

XXXXXGLPGADGRAGVMGLAGSRGASGPAGARXXXXXXXXXXXXXXXXXXGFPGSPGSqG

PAGKEGPMGLPGIDGRPGPIGPAGARGEAGNIGFPGPKXXXXXXXXXXXXXGHAGLAGARG

APGPDGNNGAQGPPGPQGVQGGKGEQGPAGPPGFQGLPGPAGTTGEVGKPGERGLPGEFG

LPGPAGPRGERGPPGqSGAAGpAGsiGsRGPSGPPGPDGNKGEPGVVGAPGNAGASGPGG

LPGERGAAGIPGPKXXXGETGLRGEIGTTGRXXXXGAPGAVGAPGPAGATGDRGEAGAAG

PAGPAGPRXXXXXXGEVGAAGPNGFAGPAGAAGQPGAKGERGTKGPKGENGGVGPTGPVG

SAGPSGPNGPPGPAGSRGDGGPPGMTGFPGAAGRTGSPGPSGITGPPGPTGAAGKXXXXX

XXGDQGPVGRTGETGASGLPGFPGEKGPAGEPGTAGPPGTAGPQGLLGAPGILGLPGSRG

ERGLPGVSGSLGEPGPLGISGPPGARGPPGAVGNPGVNGAPGEAGRDGNPGSDGPPGRXX

XXXXXXXXGYPGNAGPVGAVGAPGPHGPVGPTGKXXXXgepgpagvvgpvgvfgprGPSG

VQGARGDKGEAGEKXXXGLPGLKGHNGLQGLPGLAGHHGDQGAPGSVGPAGPRGPAGPsG

PvGKDGRTGHPGTVGPAGIRXXXXXXXXXXXXXXXXXXXXXXXXXXXXXXXXXXXXXXXXXX

>Dipodomys

QMSYGYDEKSAG--VSVPGPMGPSGPRGLPGPPGAPGPQGFQGPPGEPGEPGASGPMGPR

GPPGPPGKNGDDGEAGKPGRPGERGPSGPQGARGLPGTAGLPGMKGHRGFSGLDGAKGDA

GPAGPKGEPGSPGENGAPGQMGPRGLPGERGRPGAPGPAGARGNDGATGAAGPPGPTGPA

GPPGFPGAVGAKGEAGPQGARGSEGPQGVRGEPGPPGPAGAAGPAGNPGADGQPGAKGAN

GAPGIAGAPGFPGARGPSGPQGPSGAPGPKGNSGEPGAPGNKGDTGAKGEPGPAGVQGPP

GPAGEEGKRGARGEPGPAGLPGPPGERGGPGSRGFPGADGVAGPKGPTGERGSPGPAGPK

GSPGEAGRPGEAGLPGAKGLTGSPGSPGPDGKTGPPGPAGQDGRPGPPGPPGARGQAGVM

GFPGPKGAAGEPGKAGERGVPGPPGAVGPAGKDGEAGAQGPPGPSGPAGERGEQGPAGSP

GFQGLPGPAGPPGEAGKPGDQGVPGDLGAPGPSGARGERGFPGERGVQGPPGPAGPRGSN

GAPGNDGAKGDTGAPGAPGSQGAPGLQGMPGERGAAGLPGPKGDRGDAGPKGADGSPGKD

GVRGLTGPIGPPGPAGAPGDKGESGPSGPAGPTGARGAPGDRGEPGPPGPAGFAGPPGAD

GQPGAKGEPGDSGAKGDAGPPGPAGPAGPPGPIGNVGAPGPKGARGSAGPPGATGFPGAA

GRVGPPGPSGNAGPPGPPGPVGKEGGKGPRGETGPAGRPGEVGPPGPPGPAGEKGSPGAD

GPAGSPGTPGPQGIAGQRGVVGLPGQRGERGFPGLPGPSGEPGKQGPSGASGERGPPGPM

GPPGLAGPPGESGREGSPGAEGSPGRDGSPGPKGDRGETGPAGPPGAPGAPGAPGPVGPA

GKSGDRGETGPAGPAGPIGPVGARGPAGPQGPRGDKGETGETGERGIKGHRGFSGLQGPP

GPPGSPGEQGPSGASGPAGPRGPPGSAGAAGKDGLNGLPGPIGPPGPRGRTGDAGPVGPP

GPPGPPGPPGPPSGGFDFSFMPQPPQEKAQD-GRYYRAQYD-GKGASLGPGPMGLMGPRG

PPGASGAPGPQGFQGPAGEPGEPGQTGPAGARGPPGAPGKAGEDGHPGKPGRPGERGVVG

PQGARGFPGTPGLPGFKGIRGHNGLDGLKGQPGAPGIKGEPGAPGENGTPGQSGARGLPG

ERGRVGAPGPAGARGSDGSVGPVGPAGPIGSAGPPGFPGAPGPKGELGPVGSPGASGPAG

PRGEVGLPGLSGPVGPPGNPGANGLTGSKGAAGLPGVAGAPGLPGPRGIPGPVGAAGATG

PRGLVGEPGPAGSKGETGNKGEPGAAGPQGLPGPSGEEGKRGSNGEPGSAGPAGPPGLRG

NPGSRGLPGADGRAGVMGPPGNRGSSGPAGVRGPNGDSGRPGEPGLMGPRGLPGSPGSVG

PTGKEGPVGLPGIDGRPGPIGPAGARGEAGNIGFPGPKGPTGEPGKHGDKGHPGLAGARG

APGPDGNNGAQGPPGPQGVQGGKGEQGPAGPPGFQGLPGPSGSAGEVGKPGERGLPGEFG

LPGPAGPRGERGPPGESGAAGPSGPIGSRGPSGPPGPDGNKGEAGAVGAPGNAGASGPGG

LPGERGAAGIPGGKGEKGETGLRGEIGTPGRDGARGAPGAVGAPGPAGATGDRGEAGAAG

PAGPAGPRGSPGERGEVGPAGPNGFAGPAGAAGQPGAKGERGTKGPKGENGVVGPSGPVG

AAGPSGPNGPPGPVGGRGDGGPPGMTGFPGAAGRTGPPGPSGITGPPGPPGAAGKEGLRG

PRGDQGPVGRTGETGASGPPGFTGEKGPSGEPGTAGPPGTPGPQGLLGAPGILGLPGSRG

ERGLPGISGALGEPGPLGIAGPPGARGPPGAVGSPGVNGAPGEAGRDGNPGSDGPPGRDG

QPGHKGERGYPGNIGPTGAAGAPGPQGSVGPAGKYGNRGEPGPAGSIGPVGAVGPRGPSG

PQGIRGEKGEVGDKGHRGLPGLKGHNGLQGLPGLAGPHGDQGSPGTVGPAGPRGPAGPTG

PVGKDGRSGQPGAVGPAGVRGTQGSQGPAGPPGPPGPPGPPGISGGGYDFGYDGDFYRA

>Microtus

QMSYGYDEKSAG--VSVPGPMGPSGPRGLPGPPGAPGPQGFQGPPGEPGEPGASGPMGPR

GPPGPPGKNGDDGEAGKPGRPGERGPPGPQGARGLPGTAGLPGMKGHRGFSGLDGAKGDA

GPAGPKGEPGSPGENGAPGQMGPRGLPGERGRPGAPGPAGARGNDGATGAAGPPGPTGPT

GPPGFPGAVGAKGEAGPQGARGSEGPQGIRGEPGPPGPAGAAGPAGNPGADGQPGAKGAN

GAPGIAGAPGFPGARGPSGPQGPSGAPGPKGNSGEPGAPGNKGDTGAKGEPGPAGVQGPP

GPAGEEGKRGARGEPGPTGLPGPPGERGGPGSRGFPGADGVAGPKGPAGERGAPGPAGPK

GSPGEAGRPGEAGLPGAKGLTGSPGSPGPDGKTGPPGPAGQDGRPGPPGPPGARGQAGVM

GFPGPKGTAGEPGKTGERGIPGPPGPVGPAGKDGEAGAQGAPGPAGPAGERGEQGPAGSP

GFQGLPGPAGPPGEAGKPGEQGVPGDLGAPGPSGARGERGFPGERGVQGPPGPAGPRGNN

GAPGNDGAKGDTGAPGAPGSQGAPGLQGMPGERGAAGLPGPKGDRGDAGPKGADGSPGKD

GVRGLTGPIGPPGPAGAPGDKGETGPSGPAGPTGARGAPGDRGEPGPPGPAGFAGPPGAD

GQPGAKGEPGETGTKGDSGPPGPAGPAGPPGPIGNVGAPGPKGARGSSGPPGATGFPGAA

GRVGPPGPSGNAGPPGPPGPVGKEGGKGPRGETGPAGRPGEVGPPGPPGPAGEKGAPGAD

GPAGSPGTPGPQGIAGQRGVVGLPGQRGERGFPGLPGPSGEPGKQGPSGSSGERGPPGPM

GPPGLAGPPGESGREGSPGAEGSPGRDGSPGPKGDRGETGPAGPPGAPGAPGAPGPVGPA

GKNGDRGETGPAGPAGPIGPAGARGPAGPQGPRGDKGETGEQGDRGIKGHRGFSGLQGPP

GSPGSPGEQGPSGASGPAGPRGPPGSAGAPGKDGLNGLPGPIGPPGPRGRTGDSGPVGPP

GPPGPPGPPGPPSGGYDFSFLPQPPQEKAHD-GRYYRAQYD-GKGVSSGPGPMGLMGPRG

PPGAVGAPGPQGFQGPAGEPGEPGQTGPAGSRGPAGPPGKAGEDGHPGKPGRPGERGVVG

PQGARGFPGTPGLPGFKGIRGHNGLDGLKGQPGAQGVKGEPGAPGENGTPGQAGARGLPG

ERGRVGAPGPAGARGSDGSVGPVGPAGPIGSAGPPGFPGAPGPKGELGPVGNPGPSGPAG

PRGEVGLPGLSGPVGPPGNPGANGLTGAKGAAGLPGVAGAPGLPGPRGIPGPVGAAGATG

ARGLVGEPGPPGSKGETGNKGEPGSAGAQGPPGPSGEEGKRGSPGEPGSAGPAGPPGLRG

SPGSRGLPGADGRAGVMGPPGNRGSSGPAGVRGPNGDAGRPGEPGLMGPRGLPGSPGNVG

PSGKEGPVGLPGIDGRPGPIGPAGARGEAGNIGFPGPKGPSGDPGKAGDKGHPGLAGARG

APGPDGNNGAQGPPGPQGVQGGKGEQGPAGPPGFQGLPGPSGSAGEVGKPGERGLPGEFG

LPGPAGPRGERGPPGESGAAGPSGPVGSRGPSGAPGPDGNKGEAGAVGAPGTAGASGPGG

LPGERGAAGIPGGKGEKGETGLRGEIGNPGRDGARGAPGAVGAPGPAGATGDRGEAGAAG

PSGPAGPRGSPGERGEVGPAGPNGFAGPAGAAGQPGAKGEKGTKGPKGENGVVGPAGPVG

AAGPSGPNGPPGPAGGRGDGGPPGMTGFPGAAGRTGPPGPSGITGPPGPPGAAGKEGLRG

PRGDQGPVGRTGETGASGPPGFAGEKGPSGEPGTAGPPGTPGPQGFLGPPGILGLPGSRG

ERGLPGVAGALGEPGPLGIAGPPGARGPPGAVGSPGVNGAPGEAGRDGNPGSDGAPGRDG

QPGHKGERGYPGNIGPTGAAGAPGPHGTVGPAGKHGNRGEPGPAGSVGPAGAVGPRGPSG

PQGIRGDKGEPGDKGPRGLPGFKGHNGLQGLPGLAGLHGDQGAPGPVGPAGPRGPAGPSG

PVGKDGRSGHPGPVGPAGVRGSQGSQGPAGPPGPPGPPGPPGASGGGYDFGFEGDFYRA

>Octodon

QMSYGYDEKSVG--AAVPGPMGPSGPRGLPGPPGAPGPQGFQGPPGEPGEPGASGPMGPR

GPPGPPGKNGDDGEAGKPGRPGERGPPGPQGARGLPGTAGLPGMKGHRGFSGLDGAKGDA

GPAGPKGEPGSPGENGAPGQMGPRGLPGERGRPGPPGPAGARGNDGATGAAGPPGPTGPA

GPPGFPGAVGAKGESGPQGARGSEGPQGARGEPGPPGPAGAAGPAGNPGADGQPGAKGAN

GAPGIAGAPGFPGARGPSGPQGPSGAPGPKGNSGEPGAPGNKGDTGAKGEPGPVGVQGPP

GPAGEEGKRGARGEPGPAGLPGPPGERGGPGSRGFPGADGVAGPKGPAGERGSPGPAGPK

GSPGEAGRPGEAGLPGAKGLTGSPGSPGPDGKTGPPGPAGQDGRPGPAGPPGARGQAGVM

GFPGPKGAAGEPGKAGERGIPGPPGAVGPAGKDGEAGAQGPPGPAGPAGERGEQGPAGSP

GFQGLPGPSGPPGEGGKPGEQGIPGDLGAPGPSGARGERGFPGERGVQGPPGPAGPRGSN

GAPGNDGAKGDAGAPGAPGSQGAPGLQGMPGERGAAGLPGPKGDRGDAGPKGADGTPGKD

GPRGLTGPIGPPGPAGAPGDKGETGPSGPAGPTGARGAPGDRGEPGPPGPAGFAGPPGAD

GQPGAKGEPGDAGAKGDAGPPGPAGPAGPPGPIGNVGAPGPKGARGSAGPPGATGFPGAA

GRVGPPGPSGNAGPPGPPGPAGKEGSKGVRGETGPAGRPGEVGPPGPPGPAGEKGSPGAD

GPAGAPGTPGPQGIAGQRGVVGLPGQRGERGFPGLPGPSGEPGKQGPSGVSGERGPPGPT

GPPGLAGPPGESGREGSPGAEGSPGRDGSPGPKGDRGETGPAGPPGAPGAPGAPGPVGPA

GKSGDRGETGPAGPAGPIGPAGARGPAGPQGPRGDKGETGEQGDRGIKGHRGFSGLQGPP

GPPGSPGEQGPSGASGPAGPRGPPGSSGSPGKDGLNGLPGPIGPPGPRGRTGDAGPVGPA

GPPGPPGPPGPPSGGYDLSFLPQPPQEKSGD-GRYYRAQYD-GKGVGLGPGPMGLMGPRG

PPGAVGAPGPQGFQGPAGEPGEPGQSGPAGSRGPAGPPGKAGEDGHPGKPGRPGERGVVG

PQGARGFPGTPGLPGFKGIRGHNGMDGLKGQAGAPGVKGEPGAPGENGTPGQAGARGLPG

ERGRVGAPGPTGARGSDGSVGPVGPVGPVGAAGPPGFPGAPGPKGELGPVGNTGASGPAG

PRGEVGLPGLSGPVGPPGNPGANGLPGSKGAAGLPGVAGAPGLPGPRGIPGPVGAAGATG

ARGLVGDPGPAGSKGETGNKGEPGSAGPQGPPGPSGEEGKRGSNGEVGSAGPPGPPGLRG

TPGSRGLPGADGRAGVMGPAGSRGATGPAGVRGPNGDAGRPGEPGLMGPRGLPGSPGNVG

PAGKEGPVGLPGIDGRPGPIGPAGARGEAGNIGFPGPKGPTGDPGKNGDKGHPGLAGARG

APGPDGNNGAQGPPGPQGVQGGKGEQGPAGPPGFQGLPGPSGPAGEVGKPGERGLPGEFG

LPGPAGARGERGPPGESGAVGPSGPIGSRGPSGPPGPDGNKGEPGVVGAPGTAGASGPGG

LPGERGAAGIPGGKGEKGETGHRGEPGNTGRDGSRGAPGAIGAPGPGGATGDRGEAGAAG

PAGPAGPRGSPGERGEVGPAGPNGFAGPAGAAGQPGAKGERGTKGPKGENGVVGPTGPVG

AAGPSGPNGPPGPAGSRGDGGPPGMTGFPGAAGRTGPPGPSGITGPPGPPGPAGKEGLRG

PRGDQGPVGRAGDTGAGGPPGFVGEKGPSGEPGTAGPPGTPGPQGLLGAPGILGLPGSRG

ERGLPGIAGSSGEPGPLGLAGPPGARGPPGNVGSPGVNGAPGEAGRDGNPGSDGPPGRDG

QPGHKGERGYPGNIGPTGTAGAPGPHGPVGPSGKHGNRGEPGPAGSVGPVGAVGPRGPSG

PQGIRGDKGEVGDKGARGLPGMKGHNGLQGLPGLAGQHGDQGAPGPVGPAGPRGPAGPSG

PAGKDGRAGHPGAVGPAGVRGSQGSQGPAGPPGPPGPPGPPGVSGGGYDFGYEGDFYRA

>Chinchilla

QMSYGYDEKSVG--AAVPGPMGPSGPRGLPGPPGAPGPQGFQGPPGEPGEPGASGPMGPR

GPPGPPGKNGDDGEAGKPGRPGERGPPGPQGARGLPGTAGLPGMKGHRGFSGLDGAKGDA

GPAGPKGEPGSPGENGAPGQMGPRGLPGERGRPGPPGSAGARGNDGATGAAGPPGPTGPA

GPPGFPGAVGAKGESGPQGARGSEGPQGARGEPGPPGPAGAAGPAGNPGADGQPGAKGAN

GAPGIAGAPGFPGARGPSGPQGPSGQPGPKGNSGEPGAPGNKGDTGAKGEPGPAGVQGPP

GPAGEEGKRGARGEPGPTGLPGPPGERGGPGSRGFPGADGVAGPKGPAGERGSPGPAGPK

GSPGEAGRPGEAGLPGAKGLTGSPGSPGPDGKTGPPGPAGQDGRPGPPGPPGARGQAGVM

GFPGPKGAAGEPGKAGERGVPGPPGAVGLAGKDGEAGAQGPPGPAGPSGERGEQGPAGSP

GFQGLPGPSGPPGEAGKPGEQGVPGDLGAPGPSGARGERGFPGERGVQGPPGPAGPRGSN

GAPGNDGAKGDAGAPGAPGSQGAPGLQGMPGERGAAGLPGPKGDRGDAGPKGADGTPGKD

GPRGLTGPIGPPGPAGAPGDKGESGPSGPAGPTGARGAPGDRGEPGPPGPAGFAGPPGAD

GQPGAKGEPGDAGAKGDAGPPGPAGPAGPPGPIGNVGAPGPKGARGSAGPPGATGFPGAA

GRVGPPGPSGNAGPPGPPGPAGKEGAKGIRGETGPAGRPGEVGPPGPPGPAGEKGSPGAD

GPAGAPGTPGPQGIAGQRGVVGLPGQRGERGFPGLPGPSGEPGKQGPSGASGERGPPGPM

GPPGLAGPPGESGREGSPGAEGSPGRDGSPGPKGDRGETGPAGPPGAPGAPGAPGPVGPA

GKNGDRGETGPAGPAGPIGPAGARGPAGPQGPRGDKGETGEQGDRGIKGHRGFSGLQGPP

GPAGSPGEQGPSGASGPAGPRGPPGSAGSPGKDGLNGLPGPIGPPGPRGRTGDAGPVGPP

GPPGPPGPPGPPSGGYDLSFLPQPPQEKSGD-GRYYRAQYD-GKGVGLGPGPMGLMGPRG

PPGAVGAPGPQGFQGPAGEPGEPGQTGPAGSRGPAGPPGKAGEDGHPGKPGRPGERGVVG

PQGARGFPGTPGLPGFKGPRGHNGMDGLKGQAGAPGVKGEPGAPGENGTPGQAGARGLPG

ERGRVGAPGPAGARGSDGSVGPVGPAGPIGAAGPPGFPGAPGPKGELGPVGNTGPSGPAG

PRGELGLPGLSGPVGPPGNPGANGLAGSKGAAGLPGVAGAPGLPGPRGIPGPPGAAGATG

ARGLVGDPGPAGSKGETGNKGEPGSAGAQGPPGPSGEEGKRGSNGEAGSAGPPGPPGLRG

SPGSRGLPGADGRAGVMGPPGSRGATGPAGVRGPSGDAGRPGEPGLMGPRGLPGSPGNVG

PAGKEGPVGLPGIDGRPGPIGPAGARGEAGNIGFPGPKGPTGDPGKSGDKGHPGLAGARG

APGPDGNNGAQGPPGPQGVQGGKGEQGPAGPPGFQGLPGPSGPTGEVGKPGERGLPGEFG

LPGPAGARGERGPPGESGAVGPSGPIGSRGPSGPPGPDGNKGEPGVVGAPGTAGASGPGG

LPGERGAAGIPGGKGEKGETGHRGEPGNTGRDGARGAPGAIGAPGPAGATGDRGEAGAAG

PAGPAGPRGSPGERGEVGPAGPNGFAGPAGAAGQPGAKGERGTKGPKGENGVVGPTGPVG

AAGPSGPNGPPGPAGSRGDGGPPGMTGFPGAAGRTGPPGPSGITGPPGPPGPAGKEGLRG

PRGDQGPVGRAGDTGAGGPPGFAGEKGPSGEAGTAGPPGTPGPQGLLGAPGILGLPGSRG

ERGLPGIAGASGEPGPLGLSGPPGARGPPGNVGSPGVNGAPGEAGRDGNPGSDGPPGRDG

QPGHKGERGYPGNIGPTGAAGAPGPHGPVGPAGKHGNRGEPGPAGSVGPVGAFGPRGPTG

PQGIRGDKGEPGDKGPRGLPGMKGHNGLQGLPGLAGQHGDQGAPGPVGPAGPRGPAGPSG

PAGKDGRSGHPGAVGPAGVRGSQGSQGPAGPPGPPGPPGPPGVSGGGYDFGYEGDFYRA

>Peromyscus

QMSYGYDEKSAG--VSVPGPMGPSGPRGLPGPPGAPGPQGFQGPPGEPGEPGASGPMGPR

GPPGPPGKNGDDGEAGKPGRPGERGPSGPQGARGLPGTAGLPGMKGHRGFSGLDGAKGDA

GPAGPKGEPGSPGENGAPGQMGPRGLPGERGRPGPPGSAGARGNDGATGAAGPPGPTGPT

GPPGFPGAVGAKGEAGPQGARGSEGPQGIRGEPGPPGPAGAAGPAGNPGADGQPGAKGAN

GAPGIAGAPGFPGARGPSGPQGPSGAPGPKGNSGEPGAPGNKGDTGAKGEPGPAGVQGPP

GPAGEEGKRGARGEPGPTGLPGPPGERGGPGSRGFPGADGVAGPKGPAGERGSPGPAGPK

GSPGEAGRPGEAGLPGAKGLTGSPGSPGPDGKTGPPGPAGQDGRPGPPGPPGARGQAGVM

GFPGPKGTAGEPGKVGERGVPGPPGPVGPAGKDGEAGAQGAPGPAGPAGERGEQGPAGSP

GFQGLPGPAGPPGEAGKPGEQGVPGDLGAPGPSGARGERGFPGERGVQGPPGPAGPRGNN

GAPGNDGAKGDTGAPGAPGSQGAPGLQGMPGERGAAGLPGPKGDRGDAGPKGADGTPGKD

GPRGLTGPIGPPGPAGAPGDKGETGPSGPAGPTGARGAPGDRGEPGPPGPAGFAGPPGAD

GQPGAKGEPGETGTKGDSGPPGPAGPAGPPGPIGNVGAPGPKGPRGSAGPPGATGFPGAA

GRVGPPGPSGNAGPPGPPGPVGKEGGKGPRGETGPAGRPGEVGPPGPPGPAGEKGAPGAD

GPAGSPGTPGPQGIAGQRGVVGLPGQRGERGFPGLPGPSGEPGKQGPSGSSGERGPPGPM

GPPGLAGPPGESGREGSPGAEGSPGRDGSPGPKGDRGETGPAGPPGAPGAPGAPGPVGPA

GKSGDRGETGPAGPAGPIGPAGARGPAGPQGPRGDKGETGEQGDRGIKGHRGFSGLQGPP

GSPGSPGEQGPSGASGPAGPRGPPGSAGAPGKDGLNGLPGPIGPPGPRGRTGDSGPAGPP

GPPGPPGPPGPPSGGYDFSFLPQPPQEKAHD-GRYYRAQYD-GKGVSSGPGPMGLMGPRG

PPGAVGAPGPQGFQGPAGEPGEPGQTGPAGSRGPAGPPGKAGEDGHPGKPGRPGERGVVG

PQGARGFPGTPGLPGFKGIRGHNGLDGLKGQPGAQGVKGEPGAPGENGTPGQAGARGLPG

ERGRVGAPGPAGARGSDGSVGPVGPAGPIGSAGPPGFPGAPGPKGElGPVGNPGPSGPAG

PRGEVGLPGLSGPVGPPGNPGANGLTGAKGAAGLPGVAGAPGLPGPRGIPGPVGAAGATG

ARGLVGEPGPAGSKGETGNKGEPGSAGAQGPPGPSGEEGKRGSPGEPGSAGPGGPPGLRG

SPGSRGLPGADGRAGVMGPPGNRGSSGPAGVRGPNGDAGRPGEPGLMGPRGLPGSPGNVG

PAGKEGPVGLPGIDGRPGPIGPAGARGEAGNIGFPGPKGPSGDPGKAGDKGHPGLAGARG

APGPDGNNGAQGPPGPQGVQGGKGEQGPAGPPGFQGLPGPSGTAGEAGKPGERGLQGEFG

LPGPAGPRGERGPPGESGAAGPSGPVGSRGPSGAPGPDGNKGEAGAVGAPGSAGASGPGG

LPGERGAAGVPGGKGEKGETGLRGEIGNSGRDGARGAPGAVGAPGPGGATGDRGEAGAAG

PSGPAGPRGSPGERGEVGPAGPNGFAGPAGAAGQPGAKGEKGTKGPKGENGVVGPSGPVG

AAGPSGPNGPPGPIGSRGDGGPPGMTGFPGAAGRTGPPGPSGITGPPGPPGAAGKEGVRG

PRGDQGPVGRTGETGASGPPGFTGEKGPSGEPGTAGPPGTAGPQGFLGPPGILGLPGSRG

ERGLPGIAGALGEPGPLGIAGPPGARGPPGAVGSPGVNGAPGEAGRDGNPGSDGAPGRDG

QPGHKGERGYPGNIGPTGAAGAPGPHGSVGPAGKHGNRGEPGPAGSVGPVGAVGPRGPSG

PQGIRGDKGEPGDKGPRGLPGLKGHNGLQGLPGLSGLHGDQGSPGPVGPAGPRGPAGPSG

PAGKDGRSGHPGPVGPAGVRGSQGSQGPSGPPGPPGPPGPPGVSGGGYDFGFEGDFYRA

>Marmota

QMSYGYDEKSAG--VSVPGPMGPSGPRGLPGPPGAPGPQGFQGPPGEPGEPGASGPMGPR

GPPGPPGKNGDDGEAGKPGRPGERGPPGPQGARGLPGTAGLPGMKGHRGFSGLDGAKGDA

GPAGPKGEPGSPGENGAPGQMGPRGLPGERGRPGAPGPAGARGNDGATGAAGPPGPTGPA

GPPGFPGAVGAKGEAGPQGARGSEGPQGVRGEPGPPGPAGAAGPAGNPGADGQPGAKGAN

GAPGIAGAPGFPGARGPSGPQGPSGPPGPKGNSGEPGAPGNKGDTGAKGEPGPTGVQGPP

GPAGEEGKRGARGEPGPAGLPGPPGERGGPGSRGFPGADGVAGPKGPAGERGSPGPAGPK

GSPGEAGRPGEAGLPGAKGLTGSPGSPGPDGKTGPPGPAGQDGRPGPPGPPGARGQAGVM

GFPGPKGAAGEPGKAGERGVPGPPGAVGPAGKDGEAGAQGPPGPAGPAGERGEQGPAGSP

GFQGLPGPAGPPGEAGKPGEQGVPGDLGAPGPSGARGERGFPGERGVQGPPGPAGPRGSN

GAPGNDGAKGDAGAPGAPGSQGAPGLQGMPGERGAAGLPGPKGDRGDAGPKGADGSPGKD

GVRGLTGPIGPPGPAGAPGDKGETGPSGPAGPTGARGAPGDRGEPGPPGPAGFAGPPGAD

GQPGAKGEPGDAGAKGDAGPPGPAGPAGPPGPIGNVGAPGPKGARGSAGPPGATGFPGAA

GRVGPPGPSGNAGPPGPPGPAGKEGGKGPRGETGPAGRPGEVGPPGPPGPAGEKGSPGAD

GPAGAPGTPGPQGIAGQRGVVGLPGQRGERGFPGLPGPSGEPGKQGPSGASGERGPPGPM

GPPGLAGPPGESGREGAPGAEGSPGRDGSPGPKGDRGETGPAGPPGAPGAPGAPGPVGPA

GKSGDRGETGPAGPAGPIGPAGARGPAGPQGPRGDKGETGEQGDRGIKGHRGFSGLQGPP

GPPGSPGEQGPSGASGPAGPRGPPGSAGSPGKDGLNGLPGPIGPPGPRGRTGDAGPVGPP

GPPGPPGPPGPPSGGFDFSFMPQPPQEKAGD-GRYYRAQYD-GKGVGMGPGPMGLMGPRG

PPGAAGAPGPQGFQGPAGEPGEPGQTGPAGARGPPGAPGKAGEDGHPGKPGRPGERGVVG

PQGARGFPGTPGLPGFKGIRGHNGLDGLKGQPGAQGVKGEPGAPGENGTPGQAGARGLPG

ERGRVGAPGPAGARGSDGSVGPVGPAGPIGSAGPPGFPGAPGPKGELGPVGNPGPSGPAG

PRGEVGLPGLSGPVGPPGNPGANGLTGAKGAAGLPGVAGAPGLPGPRGIPGPVGAAGATG

ARGLVGEPGPAGSKGESGNKGEPGSAGPQGPPGPSGEEGKRGPNGEPGSAGPAGPPGLRG

NPGSRGLPGADGRAGVMGPPGNRGATGPAGVRGPNGDSGRPGEPGLMGPRGLPGSPGNVG

PAGKEGPVGLPGIDGRPGPIGPAGARGEAGNIGFPGPKGPTGDPGKSGDKGHPGLAGARG

APGPDGNNGAQGPPGPQGVQGGKGEQGPAGPPGFQGLPGPSGTTGEVGKPGERGIPGEFG

LPGPAGPRGERGPPGESGAVGPAGPIGSRGPSGPPGPDGNKGEPGVVGAPGTAGASGPGG

LPGERGAAGIPGGKGEKGEPGLRGEIGNPGRDGARGAPGAVGAPGPAGATGDRGEAGAAG

PAGPAGPRGSPGERGEVGPAGPNGFAGPAGAAGQPGAKGERGTKGPKGENGVVGPTGPVG

AAGPSGPNGPPGPAGGRGDGGPPGMTGFPGAAGRTGPPGPSGITGPPGPPGAAGKEGLRG

PRGDQGPVGRTGETGASGPPGFTGEKGPSGEPGTAGPPGTPGPQGLLGAPGILGLPGSRG

ERGLPGIAGALGEPGPLGIAGPPGARGPPGAVGSPGVNGAPGEAGRDGNPGNDGPPGRDG

QPGHKGERGYPGNIGPAGAAGAPGPHGTVGPAGKHGNRGEPGPAGSVGPVGAVGPRGPSG

PQGVRGDKGEPGDKGPRGLPGLKGHNGLQGLPGLAGQHGDQGSPGPVGPAGPRGPAGPSG

PVGKDGRSGHPGSVGPAGVRGSQGSQGPAGPPGPPGPPGPPGVSGGGYDFGYEGDFYRA

>Oryctolagus

QMSYGYDEKSAG--VSVPGPMGPSGPRGLPGPPGSPGPQGFQGPPGEPGEPGASGPMGPR

GPPGAPGKNGDDGEAGKPGRPGERGPPGPQGARGLPGTAGLPGMKGHRGFSGLDGAKGDA

GPAGPKGEPGSPGENGAPGQMGPRGLPGERGRPGAPGPAGARGNDGATGAAGPPGPTGPA

GPPGFPGAVGAKGEAGPQGARGSEGPQGVRGEPGPPGPAGAAGPAGNPGADGQPGAKGAN

GAPGIAGAPGFPGARGPSGPQGPSGPPGPKGNSGEPGAPGNKGDTGAKGEPGPTGVQGPP

GPAGEEGKRGARGEPGPTGLPGPPGERGGPGSRGFPGADGVAGPKGPAGERGAPGPAGPK

GSPGEAGRPGEAGLPGAKGLTGSPGSPGPDGKTGPPGPAGQDGRPGPPGPPGARGQAGVM

GFPGPKGAAGEPGKAGERGVPGPPGAVGPAGKDGEAGAQGPPGPAGPAGERGEQGPAGSP

GFQGLPGPAGPPGEAGKPGEQGVPGDLGAPGPSGARGERGFPGERGVQGPPGPAGPRGSN

GAPGNDGAKGDAGAPGAPGSQGAPGLQGMPGERGAAGLPGPKGDRGDAGPKGADGSPGKD

GVRGLTGPIGPPGPAGAPGDKGETGPSGPAGPTGARGAPGDRGEPGPPGPAGFAGPPGAD

GQPGAKGEPGDAGAKGDAGPAGPAGPAGPPGPIGNVGAPGPKGARGSPGPPGATGFPGAA

GRVGPPGPSGNAGPPGPPGPVGKEGGKGPRGETGPAGRPGEVGPPGPPGPAGEKGSPGAD

GPAGAPGTPGPQGIAGQRGVVGLPGQRGERGFPGLPGPSGEPGKQGPSGASGERGPPGPM

GPPGLAGPPGESGREGSPGAEGSPGRDGAPGPKGDRGETGPAGPPGAPGAPGAPGPVGPA

GKSGDRGETGPAGPAGPIGPAGARGPAGPQGPRGDKGETGEQGDRGIKGHRGFSGLQGPP

GPPGSPGEQGPSGASGPAGPRGPPGSAGAPGKDGLNGLPGPIGPSGPRGRTGDAGPVXXX

XXXXXXXXXXXXXXXXXXXXXXXXXXXXXXXXXXXXXXQFD-GKGGGPGPxxMGLMGPRG

PPGAAGAPGPQGFQGPAGEPGEPGQTGPAGARGPPGPPGKAGEDGHPGKPGRPGERGVMG

PQGARGFPGTPGLPGFKGIRGHNGLDGLKGQPGAPGVKGEPGAPGENGTPGQTGARGLPG

ERGRVGAPGPAGARGSDGSVGPVGPAGPIGSAGPPGFPGAPGPKGELGPVGNPGPSGPAG

PRGEVGLPGVSGPVGPPGNPGANGLTGAKGAAGLPGVAGAPGLPGPRGIPGPVGAAGATG

ARGLVGEPGPAGTKGESGNKGEPGSAGPQGPPGPSGEEGKRGSPGEPGSAGPAGPPGLRG

SPGSRGLPGADGRAGVMGPPGSRGSTGPAGVRGPNGDSGRPGEPGLMGPRGLPGSPGNVG

PAGKEGPVGLPGIDGRPGPIGPAGARGEPGNIGFPGPKGPTGDPGKNGDKGHPGLAGARG

APGPDGNNGAQGPPGPQGVQGGKGEQGPAGPPGFQGLPGPSGTAGEVGKPGERGLPGEFG

LPGPAGPRGERGAPGESGAAGPPGPIGSRGPSGPPGPDGNKGEPGVVGAPGTAGASGPGG

LPGERGAAGIPGGKGEKGETGLRGEIGNPGRDGARGAPGAVGAPGPAGATGDRGEAGAAG

PAGPAGPRGSPGERGEVGPAGPNGFAGPAGAAGQPGAKGEKGTKGPKGENGVVGPAGPVG

AAGPSGPNGPPGPAGGRGDGGPPGMTGFPGAAGRTGPPGPSGITGPPGPPGAAGKEGLRG

PRGDQGPVGRTGETGASGPPGFPGEKGPSGEAGTAGPPGTPGPQGLLGAPGILGLPGSRG

ERGLPGVAGALGEPGPLGIAGPPGARGPPGAVGSPGVNGAPGEAGRDGNPGSDGPPGRDG

QPGHKGERGYPGNAGPVGAAGAPGPQGSVGPTGKHGNRGEPGPAGSIGPVGAAGPRGPSG

PQGIRGDKGEPGDKGPRGLPGLKGHNGLQGLPGLAGQHGDQGAPGAVGPAGPRGPAGPTG

PAGKDGRSGHPGTVGPAGIRGSQGSQGPAGPPGPPGPPGPPGASGGGYDFGYDGDFYRA

>Ictidomys

QMSYGYDEKSAG--VSVPGPMGPSGPRGLPGPPGAPGPQGFQGPPGEPGEPGASGPMGPR

GPPGAPGKNGDDGEAGKPGRPGDRGPPGPQGARGLPGTAGLPGMKGHRGFSGLDGAKGDA

GPAGPKGEPGSPGENGAPGQMGPRGLPGERGRPGAPGPAGARGNDGATGAAGPPGPTGPA

GPPGFPGAVGAKGEAGPQGARGSEGPQGVRGEPGPPGPAGAAGPAGNPGADGQPGAKGAN

GAPGIAGAPGFPGARGPSGPQGPSGPPGPKGNSGEPGAPGNKGDPGAKGEPGPTGVQGPP

GPAGEEGKRGARGEPGPAGLPGPPGERGGPGSRGFPGADGVAGPKGPAGERGSPGPAGPK

GSPGEAGRPGEAGLPGAKGLTGSPGSPGPDGKTGPPGPAGQDGRPGPPGPPGARGQAGVM

GFPGPKGAAGEPGKTGERGVPGPPGAVGPAGKDGEAGAQGPPGPAGPAGERGEQGPAGSP

GFQGLPGPAGPPGEAGKPGEQGVPGDLGAPGPSGARGERGFPGERGVQGPPGPAGPRGSN

GAPGNDGAKGDAGAPGAPGSQGAPGLQGMPGERGAAGLPGPKGDRGDAGPKGADGSPGKD

GVRGLTGPIGPPGPAGAPGDKGETGPSGPAGPTGARGAPGDRGEAGPPGPAGFAGPPGAD

GQPGAKGEPGDAGAKGDAGPPGPAGPAGPPGPIGNVGAPGPKGARGSAGPPGATGFPGAA

GRVGPPGPSGNAGPPGPPGPAGKEGGKGPRGETGPAGRVGEVGPPGPPGPAGEKGSPGAD

GPAGAPGTPGPQGIAGQRGVVGLPGQRGERGFPGLPGPSGEPGKQGPSGASGERGPPGPM

GPPGLAGPPGESGREGSPGAEGSPGRDGSPGPKGDRGETGPAGPPGAPGAPGAPGPVGPA

GKSGDRGETGPAGPAGPIGPAGARGPAGPQGPRGDKGETGEQGDRGIKGHRGFSGLQGPP

GPPGSPGEQGPSGASGPAGPRGPPGSAGSPGKDGLNGLPGPIGPPGPRGRTGDAGPVGPP

GPPGPPGPPGPPSGGFDFSFMPQPPQEKAGD-GRYYRAQYD-GKGVGMGPGPMGLMGPRG

PPGAAGAPGPQGFQGPAGEPGEPGQTGPAGARGPPGAPGKAGEDGHPGKPGRPGERGVVG

PQGARGFPGTPGLPGFKGIRGHNGLDGLKGQPGAQGVKGEPGAPGENGTPGQAGARGLPG

ERGRVGAPGPAGARGSDGSVGPVGPAGPIGSAGPPGFPGAPGPKGELGPVGNPGPSGPAG

PRGEVGLPGLSGPVGPPGNPGANGLTGAKGAAGLPGVAGAPGLPGPRGIPGPVGAAGATG

ARGLVGEPGPAGSKGESGNKGEPGSAGPQGPPGPSGEEGKRGPNGEPGSAGPAGPPGLRG

NPGSRGLPGADGRAGVMGPPGNRGATGPAGVRGPNGDSGRPGEPGLMGPRGLPGSPGNVG

PAGKEGPVGLPGIDGRPGPIGPAGARGEAGNIGFPGPKGPTGDPGKSGDKGHPGLAGARG

APGPDGNNGAQGPPGPQGVQGGKGEQGPAGPPGFQGLPGPSGTAGEVGKPGERGLPGEFG

LPGPAGPRGERGPPGESGAVGPAGPIGSRGPSGPPGPDGNKGEPGVVGAPGTAGASGPGG

LPGERGAAGIPGGKGEKGEPGLRGEIGNPGRDGARGAPGAVGAPGPAGATGDRGEAGAAG

PAGPPGPRGSPGERGEVGPAGPNGFAGPAGAAGQPGAKGERGTKGPKGENGVVGPAGPVG

AAGPSGPNGPPGPAGGRGDGGPPGMTGFPGAAGRTGPPGPSGITGPPGPPGAAGKEGLRG

PRGDQGPVGRTGETGASGPPGFAGEKGPAGEPGTAGPPGTPGPQGLLGAPGILGLPGSRG

ERGLPGIAGALGEPGPLGIAGPPGARGPPGAVGSPGVNGAPGEAGRDGNPGSDGPPGRDG

QPGHKGERGYPGNIGPAGAAGAPGPHGTVGPAGKHGNRGEPGPAGSVGPVGAVGPRGPSG

PQGVRGDKGEPGDKGPRGLPGLKGHNGLQGLPGLAGQHGDQGSPGPVGPAGPRGPAGPSG

PVGKDGRSGHPGSVGPAGVRGSQGSQGPAGPPGPPGPPGPPGVSGGGYDFGYEGDFYRA

>Rattus

QMSYGYDEKSAG--VSVPGPMGPSGPRGLPGPPGAPGPQGFQGPPGEPGEPGASGPMGPR

GPPGPPGKNGDDGEAGKPGRPGERGPPGPQGARGLPGTAGLPGMKGHRGFSGLDGAKGDT

GPAGPKGEPGSPGENGAPGQMGPRGLPGERGRPGPPGSAGARGNDGAVGAAGPPGPTGPT

GPPGFPGAAGAKGEAGPQGARGSEGPQGVRGEPGPPGPAGAAGPAGNPGADGQPGAKGAN

GAPGIAGAPGFPGARGPSGPQGPSGAPGPKGNSGEPGAPGNKGDTGAKGEPGPAGVQGPP

GPAGEEGKRGARGEPGPSGLPGPPGERGGPGSRGFPGADGVAGPKGPAGERGSPGPAGPK

GSPGEAGRPGEAGLPGAKGLTGSPGSPGPDGKTGPPGPAGQDGRPGPAGPPGARGQAGVM

GFPGPKGTAGEPGKAGERGVPGPPGAVGPAGKDGEAGAQGAPGPAGPAGERGEQGPAGSP

GFQGLPGPAGPPGEAGKPGEQGVPGDLGAPGPSGARGERGFPGERGVQGPPGPAGPRGNN

GAPGNDGAKGDTGAPGAPGSQGAPGLQGMPGERGAAGLPGPKGDRGDAGPKGADGSPGKD

GVRGLTGPIGPPGPAGAPGDKGETGPSGPAGPTGARGAPGDRGEPGPPGPAGFAGPPGAD

GQPGAKGEPGDTGVKGDAGPPGPAGPAGPPGPIGNVGAPGPKGSRGAAGPPGATGFPGAA

GRVGPPGPSGNAGPPGPPGPVGKEGGKGPRGETGPAGRPGEVGPPGPPGPAGEKGSPGAD

GPAGSPGTPGPQGIAGQRGVVGLPGQRGERGFPGLPGPSGEPGKQGPSGASGERGPPGPM

GPPGLAGPPGESGREGSPGAEGSPGRDGAPGAKGDRGETGPAGPPGAPGAPGAPGPVGPA

GKNGDRGETGPAGPAGPIGPAGARGPAGPQGPRGDKGETGEQGDRGIKGHRGFSGLQGPP

GSPGSPGEQGPSGASGPAGPRGPPGSAGSPGKDGLNGLPGPIGPPGPRGRTGDSGPAGPP

GPPGPPGPPGPPSGGYDFSFLPQPPQEKSQDGGRYYRAQYS-DKGVSAGPGPMGLMGPRG

PPGAVGAPGPQGFQGPAGEPGEPGQTGPAGSRGPAGPPGKAGEDGHPGKPGRPGERGVVG

PQGARGFPGTPGLPGFKGIRGHNGLDGLKGQPGAQGVKGEPGAPGENGTPGQAGARGLPG

ERGRVGAPGPAGARGSDGSVGPVGPAGPIGSAGPPGFPGAPGPKGELGPVGNPGPAGPAG

PRGEAGLPGLSGPVGPPGNPGANGLTGAKGATGLPGVAGAPGLPGPRGIPGPVGAAGATG

PRGLVGEPGPAGSKGETGNKGEPGSAGAQGPPGPSGEEGKRGSPGEPGSAGPAGPPGLRG

SPGSRGLPGADGRAGVMGPPGNRGSTGPAGVRGPNGDAGRPGEPGLMGPRGLPGSPGNVG

PAGKEGPVGLPGIDGRPGPIGPAGPRGEAGNIGFPGPKGPSGDPGKPGEKGHPGLAGARG

APGPDGNNGAQGPPGPQGVQGGKGEQGPAGPPGFQGLPGPSGTAGEVGKPGERGLPGEFG

LPGPAGPRGERGPPGESGAAGPSGPIGIRGPSGAPGPDGNKGEAGAVGAPGSAGASGPGG

LPGERGAAGIPGGKGEKGETGLRGEIGNPGRDGARGAPGAIGAPGPAGASGDRGEAGAAG

PSGPAGPRGSPGERGEVGPAGPNGFAGPAGSAGQPGAKGEKGTKGPKGENGIVGPTGPVG

AAGPSGPNGPPGPAGSRGDGGPPGMTGFPGAAGRTGPPGPSGITGPPGPPGAAGKEGLRG

PRGDQGPVGRTGEIGASGPPGFAGEKGPSGEPGTTGPPGTAGPQGLLGAPGILGLPGSRG

ERGQPGIAGALGEPGPLGIAGPPGARGPPGAVGSPGVNGAPGEAGRDGNPGSDGPPGRDG

QPGHKGERGYPGNIGPTGAAGAPGPHGSVGPAGKHGNRGEPGPAGSVGPVGAVGPRGPSG

PQGIRGDKGEPGDKGARGLPGLKGHNGLQGLPGLAGLHGDQGAPGPVGPAGPRGPAGPSG

PIGKDGRSGHPGPVGPAGVRGSQGSQGPAGPPGPPGPPGPPGVSGGGYDFGFEGGFYRA

>Mus

QMSYGYDEKSAG--VSVPGPMGPSGPRGLPGPPGAPGPQGFQGPPGEPGEPGGSGPMGPR

GPPGPPGKNGDDGEAGKPGRPGERGPPGPQGARGLPGTAGLPGMKGHRGFSGLDGAKGDA

GPAGPKGEPGSPGENGAPGQMGPRGLPGERGRPGPPGTAGARGNDGAVGAAGPPGPTGPT

GPPGFPGAVGAKGEAGPQGARGSEGPQGVRGEPGPPGPAGAAGPAGNPGADGQPGAKGAN

GAPGIAGAPGFPGARGPSGPQGPSGPPGPKGNSGEPGAPGNKGDTGAKGEPGATGVQGPP

GPAGEEGKRGARGEPGPSGLPGPPGERGGPGSRGFPGADGVAGPKGPSGERGAPGPAGPK

GSPGEAGRPGEAGLPGAKGLTGSPGSPGPDGKTGPPGPAGQDGRPGPAGPPGARGQAGVM

GFPGPKGTAGEPGKAGERGLPGPPGAVGPAGKDGEAGAQGAPGPAGPAGERGEQGPAGSP

GFQGLPGPAGPPGEAGKPGEQGVPGDLGAPGPSGARGERGFPGERGVQGPPGPAGPRGNN

GAPGNDGAKGDTGAPGAPGSQGAPGLQGMPGERGAAGLPGPKGDRGDAGPKGADGSPGKD

GARGLTGPIGPPGPAGAPGDKGEAGPSGPPGPTGARGAPGDRGEAGPPGPAGFAGPPGAD

GQPGAKGEPGDTGVKGDAGPPGPAGPAGPPGPIGNVGAPGPKGPRGAAGPPGATGFPGAA

GRVGPPGPSGNAGPPGPPGPVGKEGGKGPRGETGPAGRPGEVGPPGPPGPAGEKGSPGAD

GPAGSPGTPGPQGIAGQRGVVGLPGQRGERGFPGLPGPSGEPGKQGPSGSSGERGPPGPM

GPPGLAGPPGESGREGSPGAEGSPGRDGAPGAKGDRGETGPAGPPGAPGAPGAPGPVGPA

GKNGDRGETGPAGPAGPIGPAGARGPAGPQGPRGDKGETGEQGDRGIKGHRGFSGLQGPP

GSPGSPGEQGPSGASGPAGPRGPPGSAGSPGKDGLNGLPGPIGPPGPRGRTGDSGPAGPP

GPPGPPGPPGPPSGGYDFSFLPQPPQEKSQDGGRYYRAQYS-DKGVSSGPGPMGLMGPRG

PPGAVGAPGPQGFQGPAGEPGEPGQTGPAGPRGPAGSPGKAGEDGHPGKPGRPGERGVVG

PQGARGFPGTPGLPGFKGVRGHSGMDGLKGQPGAQGVKGEPGAPGENGTPGQAGARGLPG

ERGRVGAPGPAGARGSDGSVGPVGPAGPIGSAGPPGFPGAPGPKGELGPVGNPGPAGPAG

PRGEVGLPGLSGPVGPPGNPGTNGLTGAKGATGLPGVAGAPGLPGPRGIPGPAGAAGATG

ARGLVGEPGPAGSKGESGNKGEPGSVGAQGPPGPSGEEGKRGSPGEAGSAGPAGPPGLRG

SPGSRGLPGADGRAGVMGPPGNRGSTGPAGIRGPNGDAGRPGEPGLMGPRGLPGSPGNVG

PSGKEGPVGLPGIDGRPGPIGPAGPRGEAGNIGFPGPKGPSGDPGKPGERGHPGLAGARG

APGPDGNNGAQGPPGPQGVQGGKGEQGPAGPPGFQGLPGPSGTTGEVGKPGERGLPGEFG

LPGPAGPRGERGTPGESGAAGPSGPIGSRGPSGAPGPDGNKGEAGAVGAPGSAGASGPGG

LPGERGAAGIPGGKGEKGETGLRGDTGNTGRDGARGIPGAVGAPGPAGASGDRGEAGAAG

PSGPAGPRGSPGERGEVGPAGPNGFAGPAGAAGQPGAKGEKGTKGPKGENGIVGPTGSVG

AAGPSGPNGPPGPVGSRGDGGPPGMTGFPGAAGRTGPPGPSGIAGPPGPPGAAGKEGLRG

PRGDQGPVGRTGETGASGPPGFVGEKGPSGEPGTAGAPGTAGPQGLLGAPGILGLPGSRG

ERGLPGIAGALGEPGPLGISGPPGARGPPGAVGSPGVNGAPGEAGRDGNPGSDGPPGRDG

QPGHKGERGYPGSIGPTGAAGAPGPHGSVGPAGKHGNRGEPGPAGSVGPVGAVGPRGPSG

PQGIRGDKGEPGDKGHRGLPGLKGYSGLQGLPGLAGLHGDQGAPGPVGPAGPRGPAGPSG

PVGKDGRSGQPGPVGPAGVRGSQGSQGPAGPPGPPGPPGPPGVSGGGYDFGFEGDFYRA

>Ochotona

QMSYGYDEKSAG--VSVPGPMGPSGPRGLPGPPGSPGPQGFQGPPGEPGEPGASGPMGPR

GPPGPPGKNGDDGEAGKPGRPGERGPPGPQGARGLTGTAGLPGMKGHRGFSGLDGAKGDA

GPAGPKGEPGSPGENGAPGQMGPRGLPGERGRPGPPGTAGARGNDGATGAAGPPGPTGPA

GPPGFPGAVGAKGEAGPQGARGSEGPQGIRGEPGPPGPAGAAGPAGNPGADGQPGAKGAN

GAPGIAGAPGFPGARGPSGPQGPSGPPGPKGNSGEPGAPGNKGDTGAKGEPGPAGVQGPP

GPAGEEGKRGARGEPGPAGLPGPPGERGGPGSRGFPGADGVAGPKGPAGERGAPGPAGPK

GSPGEAGRPGEAGLPGAKGLTGSPGSPGPDGKTGPPGPAGQDGRPGPPGPPGARGQAGVM

GFPGPKGAAGEPGKAGERGVPGPPGAVGAPGKDGEAGAQGPPGPAGPAGERGEQGPAGSP

GFQGLPGPAGPPGEAGKPGEQGVPGDLGAPGPSGARGERGFPGERGVQGPPGPAGPRGSN

GAPGNDGAKGDAGAPGAPGSQGAPGLQGMPGERGAAGLPGPKGDRGDAGPKGADGSPGKD

GVRGLTGPIGPPGPAGAPGDKGETGPSGPAGPTGARGAPGDRGEPGPPGPAGFAGPPGAD

GQPGAKGEPGDAGAKGDAGPPGPAGPAGPPGPIGNVGAPGPKGARGSAGPPGATGFPGAA

GRVGPPGPSGNAGPPGPPGPAGKEGGKGPRGETGPAGRPGEVGPPGPPGPAGEKGSPGAD

GPAGAPGTPGPQGITGQRGVVGLPGQRGERGFPGLPGPSGEPGKQGPSGASGERGPPGPM

GPPGLAGPPGESGREGSPGAEGSPGRDGSPGPKGDRGETGPAGPPGAPGAPGAPGPVGPA

GKSGDRGETGPAGPAGPIGPAGARGPAGPQGPRGDKGETGEQGDRGIKGHRGFSGLQGPP

GPPGSPGEQGPSGASGPAGPRGPPGSAGAPGKDGLNGLPGPIGPPGXXXXXXXXXXXXXX

XXXXXXXXXXXXXXXXXXXXXPQPPQEKAHDGGRYYRAXXXxxXXXXXXXXXMGLMGPRG

PPGAAGAPGPQGFQGPAGEPGEPGQTGPAGARGPPGAPGKAGEDGHPGKPGRPGERGIMG

PQGARGFPGTPGLPGFKGIRGHNGLDGLKGQPGAPGVKGEPGAPGENGTPGQTGARGLPG

ERGRVGAPGPAGARGSDGSVGPVGPAGPIGSAGPPGFPGAPGPKGELGPVGNPGPSGPAG

PRGEVGLPGVSGPVGPPGNPGTNGLTGAKGAAGLPGVAGAPGLPGPRGLPGPVGAAGATG

ARGLVGEPGPAGSKGESGNKGEPGSAGPQGPPGPSGEEGKRGSTGEPGSAGPPGPPGLRG

SPGSRGLPGADGRAGVMGPPGSRGSTGPAGVRGPNGDSGRPGEPGLVGPRGLPGSPGNVG

PAGKEGPVGLPGIDGRPGPIGPAGARGEPGNIGFPGPKGPSGDAGKSGDKGHPGLAGARG

APGPDGNNGAQGPPGPQGVQGGKGEQGPAGPPGFQGLPGPSGPAGEVGKPGERGLPGEFG

LPGPAGARGERGPPGESGAAGPPGPIGSRGPSGPPGPDGNKGEPGAVGAPGNAGASGPGG

LPGERGAAGIPGGKGEKGETGLRGEVGNPGRDGARGAPGAVGAPGPAGATGDRGEAGAAG

PAGPAGPRGSPGERGEVGPAGPNGFAGPAGAAGQPGAKGERGTKGPKGENGVVGPTGPVG

AAGPSGPNGPPGPVGGRGDGGPPGMTGFPGAAGRTGPPGPSGITGPPGPPGAAGKEGLRG

PRGDQGPVGRTGEPGAAGPPGFAGEKGPSGEAGTAGPPGTPGPQGLLGPPGILGLPGTRG

ERGLPGVAGALGEPGPLGVAGPPGARGPPGAVGSPGVNGAPGEAGRDGNPGSDGPPGRDG

QPGHKGERGYPGNAGPAGAAGAPGPQGSVGPTGKHGNRGEPGPAGSVGPVGAVGPRGPSG

PQGIRGDKGEPGDKGPRGLPGLKGHNGLQGLPGLAGQHGDQGAPGAVGPAGPRGPAGPTG

PAGKDGRSGHPGTVGPAGVRGSQGSQGPAGPPGPPGPPGPPGASGGGYDFGYDGDFYRA

>Myotis

QMSYGYDEKSAG--VSVPGPMGPSGPRGLPGPPGSPGPQGFQGPPGEPGEPGASGPMGPR

GPPGPPGKNGDDGEAGKPGRPGERGPPGPQGARGLPGTAGLPGMKGHRGFSGLDGAKGDA

GPAGPKGEPGSPGENGVPGQMGPRGLPGERGRPGAPGPAGARGNDGATGAAGPPGPTGPA

GPPGFPGAVGAKGEAGPQGSRGSEGPQGVRGEPGPPGPAGAAGPAGNPGADGQPGAKGAN

GAPGIAGAPGFPGARGPSGPQGPSGAPGPKGNSGEPGAPGNKGDTGAKGEPGPTGIQGPP

GPAGEEGKRGARGEPGPAGLPGPPGERGGPGSRGFPGADGVAGPKGPAGERGSPGPAGPK

GSPGEAGRPGEAGLPGAKGLTGSPGSPGPDGKTGPTGPAGQDGRPGPPGPPGARGQAGVM

GFPGPKGAAGEPGKAGERGVPGPPGAVGPAGKDGEAGAQGAPGPAGPAGERGEQGPAGSP

GFQGLPGPAGPPGEAGKPGEQGAPGDLGAPGPSGARGERGFPGERGVQGPPGPAGPRGSN

GAPGNDGAKGDAGAPGAPGSQGAPGLQGMPGERGAAGLPGPKGDRGDAGPKGADGAPGKD

GVRGLTGPIGPPGPAGAPGDKGETGPSGPAGPTGARGAPGDRGEPGPPGPAGFAGPPGAD

GQPGAKGEPGDAGAKGDAGPAGPAGPAGPPGPIGNVGAPGPKGARGSAGPPGATGFPGAA

GRVGPPGPSGNAGPPGPPGPAGKEGGKGPRGETGPAGRPGEVGPPGPPGPAGEKGSPGSD

GPAGSPGTPGPQGIAGQRGVVGLPGQRGERGFPGLPGPSGEPGKQGPSGSSGERGPPGPM

GPPGLAGPPGESGREGSPGAEGSPGRDGSPGPKGDRGETGPAGPPGAPGAPGAPGPVGPA

GKSGDRGETGPAGPAGPIGPAGARGPAGPQGPRGDKGETGEQGDRGIKGHRGFSGLQGPP

GPPGSPGDQGPSGASGPAGPRGPPGSPGAAGKDGLNGLAGPIGPPGPRGRTGDAGPVGPP

GPPGPPGPPGPPSGGFDFSFMPQPPQEKAHDGGRYYRAQFD-GKGVGGGPGPMGLMGPRG

PPGAAGAPGPQGFQGPAGEPGEPGQTGPAGSRGPAGPPGKAGEDGHPGKPGRPGERGVVG

PQGARGFPGTPGLPGFKGIRGHNGLDGLKGQPGAPGIKGEPGAPGENGTPGQTGARGLPG

ERGRVGAPGPAGARGSDGSVGPVGPAGPIGSAGPPGFPGAPGPKGELGPVGNPGPSGPAG

PRGEVGLPGLSGPVGPPGNPGANGLAGAKGAAGLPGVAGAPGLPGPRGIPGPPGAAGAAG

PRGLIGEPGPAGSKGETGNKGEPGSAGAQGPPGPSGEEGKRGTAGEAGPAGPPGPAGLRG

NPGSRGLPGADGRAGVMGPAGPRGATGPAGARGPNGDAGRPGEPGLMGPRGFPGSPGNVG

PAGKEGPVGLPGIDGRPGPIGPAGARGEPGNIGFPGPKGPTGDAGKPGERGHAGLAGARG

APGPDGNNGAQGPPGPQGVQGGKGEQGPAGPPGFQGLPGPAGTAGEAGKPGERGLPGEFG

LPGPAGPRGERGPPGESGAVGPSGPIGSRGPSGPPGPDGNKGEPGSVGAPGSAGAPGPGG

LPGERGAAGIPGGKGDKGEPGLRGEMGTTGRDGARGAPGAMGAPGPSGASGDRGEAGAAG

PAGPAGPRGSPGERGEVGPAGPNGFAGPAGAAGQPGAKGERGTKGPKGENGVVGPTGPVG

AAGPSGPNGPPGPAGTRGDGGPPGMTGFPGAAGRTGPPGPSGITGPPGPPGASGKEGLRG

PRGDQGPVGRTGETGATGPPGFVGEKGPSGEPGAAGPPGTPGPQGLLGAPGILGLPGSRG

ERGLPGVSGSVGEPGPLGIAGPPGARGPPGAVGSPGVNGAPGEAGRDGNPGSDGPPGRDG

QPGHKGDRGYPGNAGPVGTVGAPGPHGPVGPTGKHGNRGEPGPAGSVGPTGAVGPRGPSG

AQGIRGDKGEPGEKGPRGLPGLKGHNGLQGLPGLAGHHGDQGAPGTVGPAGPRGPAGPSG

PPGKDGRNGHPGVVGPAGIRGTQGSQGPAGPPGPPGPPGPPGISGGGYDFGFDGDFYRA

>Pteropus

QMSYGYDEKSAG--VSVPGPMGPSGPRGLPGPPGAPGPQGFQGPPGEPGEPGASGPMGPR

GPPGPPGKNGDDGEAGKPGRPGERGPPGPQGARGLPGTAGLPGMKGHRGFSGLDGAKGDS

GPAGPKGEPGSPGENGAPGQMGPRGLPGERGRPGAPGPAGARGNDGATGAAGPPGPTGPA

GPPGFPGAVGAKGEAGPQGSRGSEGPQGVRGEPGPPGPAGAAGPAGNPGADGQPGAKGAN

GAPGIAGAPGFPGARGPSGPQGPGGPPGPKGNSGEPGAPGNKGDAGAKGEPGPTGIQGPP

GPAGEEGKRGARGEPGPSGLPGPPGERGGPGSRGFPGADGVAGPKGPAGERGSPGPAGPK

GSPGEAGRPGEAGLPGAKGLTGSPGSPGPDGKTGPAGPAGQDGRPXPPGPPXARGQAGVM

GFPGXXXXXGEPGKAGERGVPGPPGAVGAAGKDGEAGAQGPPGPAGPAGERGEQGPAGSP

GFQGLPGPSGPPGEAGKPGEQGVPGDLGAPGPSGARGERGFPGERGVQGPPGPAGPRGAN

GAPGNDGAKGDAGAPGAPGSQGAPGLQGMPGERGAAGLPGPKGDRGDAGPKGADGAPGKD

GVRGLTGPIGPPGPAGAPGDKGESGPSGPAGPTGARGAPGDRGEPGPPGPAGFAGPPGAD

GQPGAKGEPGDAGAKGDAGPAGPAGPAGPPGPIGNVGAPGPKGARGSAGPPGATGFPGAA

GRVGPPGPSGNAGPPGPPGPVGKEGGKGPRGETGPAGRPGEAGPPGPPGPAGEKGSPGAD

GPAGAPGTPGPQGIAGQRGVVGLPGQRGERGFPGLPGPSGEPGKQGPSGTSGERGPPGPM

GPPGLAGPPGESGREGSPGAEGSPGRDGSPGPKGDRGETGPAGAPGAPGAPGAPGPVGPA

GKSGDRGETGPAGPAGPVGPVGARGPTGPQGPRGDKGETGEQGDRGIKGHRGFSGLQGPP

GPPXXXXXXXXXXXXXXXXXXGPPGSAGAAGKDGLNGLPGPIGPPGPRGRTGDAGPVGPP

GPPGPPGPPGPPSGGFDFSFLPQPPQEKAHDGGRYYRAQYD-GKGVGLGPGPMGLMGPRG

PPGAAGAPGPQGFQGPAGEPGEPGQTGPAGARGPTGPPGKAGEDGHPGKPGRPGERGVVG

PQGARGFPGTPGLPGFKGIRGHNGLDGLKGQPGAPGIKXXXXXXXXXXXXXXXGARGLPG

ERGRVGAPGPAGARGSDGSVGPVGPAGPIGSAGPPGFPGAPGPKGELGPVGNPGPAGPAG

PRGEVGLPGLSGPVGPPGNPGANGLTGAKGAAGLPGVAGAPGLPGPRGIPGPPGAVGATG

ARGLVGEPGPAGSKGESGNKGEPGSAGAQGPPGPSGEEGKRGSNGEAGSAGPPGPPGLRG

SPGSRGLPGADGRAGVMGPAGSRGATGPAGVRGPSGDSGRXXXXGLMGPRGFPGSPGNVG

PAGKEGPMGLPGIDGRPGPIGPAGARGEPGNIGFPGPKGPTGDPGKSGEKGHAGLAGPRG

APGPDGNNGAQGPPGLQGVQGGKGEQGPAGPPGFQGLPGPAGTTGEVGKPGERGLPGEFG

LPGPAGPRGERGPPGESGAVGPSGPIGSRGPSGPPGPDGNKGEPGGVGAPGTAGASGSGG

LPGERGAAGIPGGKGEKGETGLRGEVGSTGRDGARGAPGAIGAPGPAGATGDRGEAGPAG

PAGPAGPRGSPGERGEVGPAGPNGFAGPAGAAGQPGAKGERGTKGPKGENGPVGPTGPAG

SSGPAGPNGPPGPAGSRGDGGPPXXXXXXGAAGRTGPSGPSGITGPPGPPGAAGKEGVRG

PRGDQGPVGRTGETGAGGPPGFTGEKGPSGEPGTAGPPGTPGPQGLLGAPGILGLPGSRG

ERGLPGVAGSVGEPGPLGISGPPGARGPPGAVGNPGVNGAPGEAGRDGNPGNDGPPGRDG

QPGHKGERGYPGNPGPVGALGAPGPHGPVGPTGKHGNRGEPGPAGSVGPTGAVGPRGPSG

PQGIRGDKGEPGDKGPRGLPGLKGHNGLQGLPGLAGHHGDQGSPGSVGPAGPRGPAGPSG

PAGKDGRTGHPGTVGPAGIRGSQGSQGPAGPPGPPGPPGPPGVSGGGYDFGFDGDFYRA

>Odocoileus

QLSYGYDEKSTG--ISVPGPMGPSGPRGLPGPPGAPGPQGFQGPPGEPGEPGASGPMGPR

GPPGPPGKNGDDGEAGKPGRPGERGPPGPQGARGLPGTAGLPGMKGHRGFSGLDGAKGDA

GPAGPKGEPGSPGENGAPGQMGPRGLPGERGRPGAPGPAGARGNDGATGAAGPPGPTGPA

GPPGFPGAVGAKGEAGPQGPRGSEGPQGVRGEPGPPGPAGAAGPAGNPGADGQPGGKGAN

GAPGIAGAPGFPGARGPSGPQGPSGPPGPKGNSGEPGAPGSKGDTGAKGEPGPTGIQGPP

GPAGEEGKRGARGEPGPAGLPGPPGERGGPGSRGFPGADGVAGPKGPAGERGSPGPAGPK

GSPGEAGRPGEAGLPGAKGLTGSPGSPGPDGKTGPPGPAGQDGRPGPPGPPGARGQAGVM

GFPGPKGAAGEPGKAGERGVPGPPGAVGPAGKDGEAGAQGPPGPAGPAGERGEQGPAGSP

GFQGLPGPAGPPGEAGKPGEQGVPGDLGAPGPSGARGERGFPGERGVQGPPGPAGPRGAN

GAPGNDGAKGDAGAPGAPGSQGAPGLQGMPGERGAAGLPGPKGDRGDAGPKGADGAPGKD

GVRGLTGPIGPPGPAGAPGDKGETGPSGPAGPTGARGAPGDRGEPGPPGPAGFAGPPGAD

GQPGAKGEPGDAGAKGDAGPPGPAGPAGPPGPIGNVGAPGPKGARGSAGPPGATGFPGAA

GRVGPPGPSGNAGPPGPPGPAGKEGSKGPRGETGPAGRPGEVGPPGPPGPAGEKGAPGAD

GPAGAPGTPGPQGIAGQRGVVGLPGQRGERGFPGLPGPSGEPGKQGPSGASGERGPPGPM

GPPGLAGPPGESGREGAPGAEGSPGRDGSPGPKGDRGETGPAGPPGAPGAPGAPGPVGPA

GKSGDRGETGPAGPAGPIGPVGARGPAGPQGPRGDKGETGEQGDRGIKGHRGFSGLQGPP

GPPGSPGEQGPSGASGPAGPRGPPGSAGTPGKDGLNGLPGPIGPPGPRGRTGDAGPAGPP

GPPGPPGPPGPPSGGYDLSFLPQPPQEKAHDGGRYYRAQFD-GKG-G-GPGPMGLMGPRG

PPGASGAPGPQGFQGPPGEPGEPGQTGPAGARGPPGPPGKAGEDGHPGKPGRPGERGVVG

PQGARGFPGTPGLPGFKGIRGHNGLDGLKGQPGAPGVKGEPGAPGENGTPGQTGARGLPG

ERGRVGAPGPAGARGSDGSVGPVGPAGPIGSAGPPGFPGAPGPKGELGPVGNPGPAGPAG

PRGEVGLPGLSGPVGPPGNPGANGLPGAKGAAGLPGVAGAPGLPGPRGIPGPVGAAGATG

ARGLVGEPGPAGSKGESGNKGEPGAVGQPGPPGPSGEEGKRGSTGEIGPAGPPGPPGLRG

NPGSRGLPGADGRAGVMGPAGSRGAMGPAGVRGPNGDSGRPGEPGLMGPRGFPGSPGNIG

PAGKEGPVGLPGIDGRPGPIGPAGARGEPGNIGFPGPKGPTGDPGKAGEKGHAGLAGARG

APGPDGNNGAQGPPGPQGVQGGKGEQGPAGPPGFQGLPGPAGTAGEAGKPGERGIPGEFG

LPGPAGARGERGPPGESGAAGPTGPIGSRGPSGPPGPDGNKGEPGVVGAPGTAGPSGPSG

LPGERGAAGIPGGKGEKGETGLRGDIGSPGRDGARGAPGAVGAPGPAGANGDRGEAGPAG

PAGPAGPRGSPGERGEVGPAGPNGFAGPAGAAGQAGAKGERGTKGPKGENGPVGPTGPVG

AAGPSGPNGPPGPAGSRGDGGPPGATGFPGAAGRTGPPGPSGISGPPGPPGPAGKEGLRG

PRGDQGPVGRSGETGASGPPGFAGEKGPSGEPGTAGPPGTPGPQGLLGPPGFLGLPGSRG

ERGLPGVAGSVGEPGPLGIAGPPGARGPPGNVGNPGVNGAPGEAGRDGNPGNDGPPGRDG

QPGHKGERGYPGNAGPVGTAGAPGPQGPVGPTGKHGNRGEPGPAGAVGPAGAVGPRGPSG

PQGIRGDKGEPGDKGPRGLPGLKGHNGLQGLPGLAGHHGDQGAPGAVGPAGPRGPAGPSG

PAGKDGRTGQPGAVGPAGIRGSQGSQGPAGPPGPPGPPGPPGPSGGGYDFGFDGDFYRA

>Bos

QLSYGYDEKSTG--ISVPGPMGPSGPRGLPGPPGAPGPQGFQGPPGEPGEPGASGPMGPR

GPPGPPGKNGDDGEAGKPGRPGERGPPGPQGARGLPGTAGLPGMKGHRGFSGLDGAKGDA

GPAGPKGEPGSPGENGAPGQMGPRGLPGERGRPGAPGPAGARGNDGATGAAGPPGPTGPA

GPPGFPGAVGAKGEGGPQGPRGSEGPQGVRGEPGPPGPAGAAGPAGNPGADGQPGAKGAN

GAPGIAGAPGFPGARGPSGPQGPSGPPGPKGNSGEPGAPGSKGDTGAKGEPGPTGIQGPP

GPAGEEGKRGARGEPGPAGLPGPPGERGGPGSRGFPGADGVAGPKGPAGERGAPGPAGPK

GSPGEAGRPGEAGLPGAKGLTGSPGSPGPDGKTGPPGPAGQDGRPGPPGPPGARGQAGVM

GFPGPKGAAGEPGKAGERGVPGPPGAVGPAGKDGEAGAQGPPGPAGPAGERGEQGPAGSP

GFQGLPGPAGPPGEAGKPGEQGVPGDLGAPGPSGARGERGFPGERGVQGPPGPAGPRGAN

GAPGNDGAKGDAGAPGAPGSQGAPGLQGMPGERGAAGLPGPKGDRGDAGPKGADGAPGKD

GVRGLTGPIGPPGPAGAPGDKGEAGPSGPAGPTGARGAPGDRGEPGPPGPAGFAGPPGAD

GQPGAKGEPGDAGAKGDAGPPGPAGPAGPPGPIGNVGAPGPKGARGSAGPPGATGFPGAA

GRVGPPGPSGNAGPPGPPGPAGKEGSKGPRGETGPAGRPGEVGPPGPPGPAGEKGAPGAD

GPAGAPGTPGPQGIAGQRGVVGLPGQRGERGFPGLPGPSGEPGKQGPSGASGERGPPGPM

GPPGLAGPPGESGREGAPGAEGSPGRDGSPGAKGDRGETGPAGPPGAPGAPGAPGPVGPA

GKSGDRGETGPAGPAGPIGPVGARGPAGPQGPRGDKGETGEQGDRGIKGHRGFSGLQGPP

GPPGSPGEQGPSGASGPAGPRGPPGSAGSPGKDGLNGLPGPIGPPGPRGRTGDAGPAGPP

GPPGPPGPPGPPSGGYDLSFLPQPPQEKAHDGGRYYRAQFD-AKG-G-GPGPMGLMGPRG

PPGASGAPGPQGFQGPPGEPGEPGQTGPAGARGPPGPPGKAGEDGHPGKPGRPGERGVVG

PQGARGFPGTPGLPGFKGIRGHNGLDGLKGQPGAPGVKGEPGAPGENGTPGQTGARGLPG

ERGRVGAPGPAGARGSDGSVGPVGPAGPIGSAGPPGFPGAPGPKGELGPVGNPGPAGPAG

PRGEVGLPGLSGPVGPPGNPGANGLPGAKGAAGLPGVAGAPGLPGPRGIPGPVGAAGATG

ARGLVGEPGPAGSKGESGNKGEPGAVGQPGPPGPSGEEGKRGSTGEIGPAGPPGPPGLRG

NPGSRGLPGADGRAGVMGPAGSRGATGPAGVRGPNGDSGRPGEPGLMGPRGFPGSPGNIG

PAGKEGPVGLPGIDGRPGPIGPAGARGEPGNIGFPGPKGPSGDPGKAGEKGHAGLAGARG

APGPDGNNGAQGPPGLQGVQGGKGEQGPAGPPGFQGLPGPAGTAGEAGKPGERGIPGEFG

LPGPAGARGERGPPGESGAAGPTGPIGSRGPSGPPGPDGNKGEPGVVGAPGTAGPSGPSG

LPGERGAAGIPGGKGEKGETGLRGDIGSPGRDGARGAPGAIGAPGPAGANGDRGEAGPAG

PAGPAGPRGSPGERGEVGPAGPNGFAGPAGAAGQPGAKGERGTKGPKGENGPVGPTGPVG

AAGPSGPNGPPGPAGSRGDGGPPGATGFPGAAGRTGPPGPSGISGPPGPPGPAGKEGLRG

PRGDQGPVGRSGETGASGPPGFVGEKGPSGEPGTAGPPGTPGPQGLLGAPGFLGLPGSRG

ERGLPGVAGSVGEPGPLGIAGPPGARGPPGNVGNPGVNGAPGEAGRDGNPGNDGPPGRDG

QPGHKGERGYPGNAGPVGAAGAPGPQGPVGPVGKHGNRGEPGPAGAVGPAGAVGPRGPSG

PQGIRGDKGEPGDKGPRGLPGLKGHNGLQGLPGLAGHHGDQGAPGAVGPAGPRGPAGPSG

PAGKDGRIGQPGAVGPAGIRGSQGSQGPAGPPGPPGPPGPPGPSGGGYEFGFDGDFYRA

>Delphinapterus

QMSYGYDEKSTG--ISVPGPMGPSGPRGLPGPPGAPGPQGFQGPPGEPGEPGASGPMGPR

GPPGPPGKNGDDGEAGKPGRPGERGPPGPQGARGLPGTAGLPGMKGHRGFSGLDGAKGDA

GPAGPKGEPGSPGENGAPGQMGPRGLPGERGRPGAPGPAGARGNDGATGAAGPPGSTGPA

GPPGFPGAVGAKGEAGPQGSRGSEGPQGVRGEPGPPGPAGAAGPAGNPGTDGQPGAKGAN

GAPGIAGAPGFPGARGPSGPQGPSGPPGPKGNSGEPGAPGNKGDTGAKGEPGPTGIQGPP

GPAGEEGKRGARGEPGPAGLPGPPGERGGPGSRGFPGSDGLAGPKGPAGERGAPGPAGPK

GSPGEAGRPGEAGLPGAKGLTGSPGSPGPDGKTGPPGPAGQDGRPGPPGPPGSRGQAGVM

GFPGPKGAAGEPGKAGERGVPGPPGAAGPAGKDGEAGAQGPPGPAGPAGERGEQGPAGSP

GFQGLPGPSGPPGEAGKPGEQGVPGDLGAPGPSGARGERGFPGERGVQGPSGPAGPRGSN

GAPGNDGAKGDAGAPGAPGNQGAPGLQGMPGERGAAGLPGPKGDRGDAGPKGADGAPGKD

GVRGLTGPIGPPGPAGAPGDKGETGPSGPAGPTGARGAPGDRGEPGPPGPAGFAGPPGAD

GQPGAKGEPGDAGAKGDAGAPGTAGPTGPPGPIGNVGAPGPKGARGSAGPPGATGFPGAA

GRVGPPGPSGNAGPPGPSGPAGKEGSKGPRGETGPAGRAGEVGPPGPPGPAGEKGAPGSD

GPAGAPGSPGPQGIAGQRGVVGLPGQRGERGFPGLPGPSGEPGKQGPSGASGERGPPGPM

GPPGLAGPPGESGREGAPGAEGSPGRDGSPGPKGDRGETGPAGPPGAPGAPGAPGPVGPA

GKSGDRGETGPAGPAGPIGPVGARGPTGPQGPRGDKGETGEQGDRGIKGHRGFSGLQGPP

GPPGSPGEQGPSGASGPAGPRGPPGSAGTPGKDGLNGLPGPIGPPGPRGRTGDAGPAGPP

GPPGPPGPPGPPSGGYDFSFLPQPPQEKAQDGGRYYRAQYD-GKGVGLGPGPMGLMGPRG

PPGASGAPGPQGFQGPPGEPGEPGQTGPAGARGPPGPPGKAGEDGHPGKPGRPGERGVVG

PQGARGFPGTPGLPGFKGIRGHNGLDGLKGQPGTPGVKGEPGAPGENGIPGQIGARGLPG

ERGRVGAPGPAGARGSDGSVGPVGPAGPIGSAGPPGFPGAPGPKGELGPVGNPGPAGPAG

SRGEVGLPGVSGPVGPPGNPGANGLHGAKGAAGLPGVAGAPGLPGPRGIPGPVGAAGATG

ARGLVGEPGPAGSKGESGSKGEPGAAGPTGPPGPSGEEGKRGTTGEIGSAGPPGPPGLRG

NPGSRGLPGADGRAGVMGPHGSRGGTGPAGVRGPSGDSGRPGEPGLMGPRGFPGSPGNVG

PAGKEGPMGLPGIDGRPGPIGPAGTRGEPGNIGFPGPKGPTGDPGKNGEKGHAGLAGPRG

APGPDGNNGAQGPPGPQGVSGGKGEQGPAGPPGFQGLPGPAGTAGEAGKAGERGIPGEFG

LPGPAGSRGERGPPGESGAAGPAGPIGSRGPSGPAGPDGNKGEPGVVGAPGTAGPSGPNG

LPGERGAAGIPGGKGEKGETGLRGDVGSHGRDGARGAPGAVGAPGPAGANGDRGEAGPAG

PAGPVGPRGSPGERGEVGPAGPNGFAGPAGAAGQPGAKGERGTKGPKGENGPTGPTGPVG

AVGPAGPNGPPGPAGSRGDGGPPGATGFPGAAGRTGPPGPSGITGPPGPPGPAGKEGLRG

PRGDQGPVGRTGETGASGPPGFVGEKGPSGEPGTAGSPGTPGPQGLLGVPGFLGLPGSRG

ERGLPGVAGSMGEPGPLGIAGPAGARGPPGAVGNPGVNGAPGEAGRDGNPGNDGPPGRDG

QAGHKGDRGYPGNAGPTGTAGAPGPQGPVGPTGKHGNRGEPGPSGPAGLAGAVGPRGPSG

PQGIRGDKGEPGDKGPRGLPGLKGHNGLQGLPGLAGHHGDQGAPGTVGPAGPRGPSGPSG

PSGKDGRTGHPGAVGPAGIRGSQGSQGPSGPPGPPGPPGPPGPSGGGYDFGFEGDFYRA

>Tursiops

QMSYGYDEKSTG--ISVPGPMGPSGPRGLPGPPGTPGPQGFQGPPGEPGEPGASGPMGPR

GPPGPPGKNGDDGEAGKPGRPGERGPPGPQGARGLPGTAGLPGMKGHRGFSGLDGAKGDA

GPAGPKGEPGSPGENGAPGQMGPRGLPGERGRPGAPGPAGARGNDGATGAAGPPGSTGPA

GPPGFPGAVGAKGEAGPQGSRGSEGPQGVRGEPGPPGPAGATGPAGNPGADGQPGAKGAN

GAPGIAGAPGFPGARGPSGPQGPSGPPGPKGNSXXXXXXXXXXXXXXXXXXXXXXXXXXX

XXXXXXXXXXXXXXXXXXXXXXXXXXXXXXXXXXXXXXXXXXXXXxGPAERGAPGLAGPK

ASPGEAGRPGEAGLPGAKGLTGSPGSPGPDGKTGPPGPAGQDGRPGPPGPPGSRGQAGVM

GFPGPKGAAGEPGKAGERGVPGPPGAAGPAGKDGEAGAQGPPGPAGPAGERGEQGPAGSP

GFQGLPGPSGPPGEAGKPGEQGAPGDLGAPGPSGARGERGFPGERGVQGPSGPAGPRGSN

GPPGNDGAKGDAGAPGAPGNQGAPGLQGMPGERGAAGLPGPKGDRGDAGPKGADGAPGKD

GVRGLTGPIGPPGPAGAPGDKGETGPSGPAGPTGARGAPGDRGEPGPPGPAGFAGPPGAD

GQPGAKGEPGDAGAKGDAGAPGSAGPTGPPGPIGNVGAPGPKGARGSAGPPGATGFPGAA

GRVGPPGPSGNAGPPGPSGPAGKEGSKGPRGETGPAGRAGEVGPPGPPGPAGEKGAPGAD

GPAGSPGSPGPQGIAGQRGVVGLPGQRGERGFPGLPGPSGEPGKQGPSGASGERGPPGPM

GPPGLAGPPGESGREGAPGAEGSPGRDGSPGPKGDRGETGPAGPPGAPGSPGAPGPVGPA

GKSGDRGETGPAGPAGPIGPAGARGPTGPQGPRGDKGETGEQGDRGIKGHRGFSGLQGPP

GPPGSPGEQGPSGASGPAGPRGPPGSAGTPGKDGLNGLPGPIGPPGPRGRTGDAGPAGPP

GPPGPPGPPGPPSGGYDFSFLPQPPQEKAQDGGRYYRAQYD-GKGVGLGPGPMGLMGPRG

PPGASGVPGPQGFQGPPGEPGEPGQTGPAGARGPPGPPGKAGEDGHPGKPGRPGERGVVG

PQGARGFPGTPGLPGFKGIRGHNGLDGLKGQPGTPGVKGEPGAPGENGIPGQVGARGLPG

ERGRVGAPGPAGARGSDGSVGPVGPAGPVGSAGPPGFPGAPGPKGELGPVGNPGPAGPAG

SRGEVGLPGVSGPVGPPGNPGANGLHGAKGAAGLPGVAGAPGLPGPRGIPGPVGAAGATG

ARGLVGEPGPAGSKGESGNKGEPGAAGPTGPPGPSGEEGKRGSTGEIGSAGPPGPPGLRG

NPGSRGLPGADGRAGVMGPHGSRGGTGPAGVRGPSGDSGRPGEPGLMGPRGFPGSPGNVG

PAGKEGPMGLPGIDGRPGPIGPAGARGEPGNIGFPGPKGPTGDPGKNGEKGHAGLAGPRG

APGPDGNNGAQGPPGPQGVSG-KGEQGPAGPPGFQGLPGPAGTAGEAGKAGERGIPGEFG

LPGPAGPRGERDPPGESxAAGPTGPVGSRGPSGPAXXXXXXGEPGVVGAPGSAGPSGPNG

LPGERGAAGIPGGKGEKGETGLRGDAGSHGRDGARGAPGAVGAPGPAGANGDRGEAGPAG

PAGPAGPRGSPGERGEVGPAGPNGFAGPAGAAGQPGAKGERGTKGPKGENGPTGPTGPVG

AAGPAGPNGPPGPAGSRGDGGPPGATGFPGAAGRTGPPGPSGITGPPGPPGPAGKEGLRG

PRGDQGPVGRTGETGASGPPGFVGEKGPSGEPGTAGSPGTPGPQGLLGAPGFLGLPGSRG

ERGLPGVAGSVGEPGPLGIAGPTGARGPPGAVGNPGVNGAPGEAGRDGNPGNDGPPGRDG

QAGHKGDRGYPGNAGPTGTVGAPGPQGPVGPTGKHGHRGEPGPSGPIGLAGAVGPRGPSG

PQGIRGDKGEPGDKGPRGLPGLKGHNGLQGLPGLAGHHGDQGAPGTVGPAGPRGPSGPSG

PSGKDGRTGHPGAVGPAGIRGSQGSQGPSGPPGPPGPPGPPGPSGGGYDFGFDGDFYRA

>Balaenoptera

QMSYGYDEKSTG--ISVPGPMGPSGPRGLPGPPGAPGPQGFQGPPGEPGEPGASGPMGPR

GPPGPPGKNGDDGEAGKPGRPGERGPPGPQGARGLPGTAGLPGMKGHRGFSGLDGAKGDA

GPAGPKGEPGSPGENGAPGQMGPRGLPGERGRPGASGPAGARGNDGATGAAGPPGPTGPA

GPPGFPGAVGAKGEAGPQGSRGSEGPQGVRGEPGPPGPAGATGPAGNPGTDGQPGAKGAN

GAPGIAGAPGFPGARGPSGPQGPSGPPGPKGNSGEPGAPGNKGDTGAKGEPGPTGIQGPP

GPAGEEGKRGTRGEPGPAGLPGPPGERGGPGSRGFPGADGVSGPKGPAGERGAPGPAGPK

GSPGEAGRPGEAGLPGAKGLTGSPGSPGPDGKTGPPGPAGQDGRPGPPGPPGSRGQAGVM

GFPGPKGAAGEPGKAGERGVPGPPGAAGAAGKDGETGAQGPPGPAGPAGERGEQGPAGSP

GFQGLPGPAGPPGEAGKPGEQGAPGDVGAPGPSGARGERGFPGERGVQGPSGPAGPRGSN

GAPGNDGAKGDAGAPGAPGNQGAPGLQGMPGERGAAGLPGLKGDRGDVGPKGADGAPGKD

GVRGLTGPIGPPGPAGAPGDKGETGPSGPAGPTGARGAPGDRGEPGPPGPAGFAGPPGAD

GQPGAKGEPGDAGAKGDAGLPGAAGPTGPPGPMGNVGAPGPKGARGSAGPPGATGFPGAA

GRVGPPGPSGNAGPPGPSGPAGKEGSKGARGETGPAGRAGEVGPPGPPGPAGEKGAPGAD

GPAGAPGSPGPQGIAGQRGVVGLPGQRGERGFPGLPGPSGEPGKQGPSGASGERGPPGPM

GPPGLAGPPGESGREGAPGAEGSPGRDGSPGPKGDRGETGPAGPPGAPGAPGAPGPVGPA

GKSGDRGETGPAGPAGPIGPVGARGPTGPQGPRGDKGETGEQGDRGIKGHRGFSGLQGPP

GPPGSPGEQGPSGASGPAGPRGPPGSAGTPGKDGLNGLPGPIGPPGPRGRTGDAGPAGPP

GPPGPPGPPGPPSGGYDFSFMPQPPQEKAQDGGRYYRAQND-GKGVGLGPGPMGLMGPRG

PPGASGAPGPQGFQGLPGEPGEPGQTGPAGSRGPPGPPGKAGEDGHPGKPGRPGERGVVG

PQGARGFPGTPGLPGFKGIRGHNGLDGLKGQPGTPGVKGEPGAPGENGIPGQIGARGLPG

ERGRVGAPGPAGARGSDGSVGPVGPAGPIGSAGPPGFPGAPGPKGELGPVGNPGPPGPAG

SRGEVGLAGVSGPVGPPGNPGANGLPGAKGAAGLPGVAGAPGLPGPRGIPGPVGAAGATG

ARGLIGEPGPAGSKGESGNKGEPGAAGPTGPPGPSGEEGKRGTTGEIGSAGPPGPPGLRG

NPGSRGLPGADGRAGVMGPHGSRGGTGPAGMRGPSGDSGRPGEPGLMGPRGFPGSPGNVG

PAGKEGPMGLPGIDGRPGAIGPAGARGEPGNIGFPGPKGPTGDPGKNGEKGHAGLAGPRG

APGPEGNNGAQGPPGLQGVSGGKGEQGPAGPPGFQGLPGPSGTAGEAGKAGERGTPGEFG

LPGPAGPRGERGPPGESGAAGPAGPIGNRGPSGPAGPDGNKGEPGVVGAPGTAGPSGPSG

LPGERGAAGIPGGKGEKGETGLRGDAGSHGRDGARGAPGAVGAPGPAGANGDRGEAGPAG

SAGPAGPRGMPGERGEVGPAGPNGFAGPAGAAGQPGAKGERGTKGPKGENGPAGPTGPVG

AAGPSGPNGPPGPAGSRGDGGPPGVTGFPGAAGRTGPPGPSGITGPPGPTGPAGKEGLRG

PRGDQGPVGRTGETGASGPPGFVGEKGPSGEPGTAGSPGTPGPQGLLGAPGFLGLPGSRG

ERGLPGVAGSVGEPGPLGISGPTGARGPPGAVGNPGVNGAPGEAGRDGNPGSDGPPGRDG

QAGHKGDRGYPGNAGPTGTAGAPGPQGPVGPTGKHGNRGESGPSGPTGLAGAVGPRGPSG

PQGIRGDKGEPGDKGPRGLPGLKGHNGLQGLPGFAGHHGDQGAPGTVGPAGPRGPAGPSG

PSGKDGRTGHPGAVGPAGIRGSQGSQGPAGPPGPPGPPGPPGQSGGGYDFGFEGDFYRA

>Sus

QLSYGYDEKSAG--ISVPGPMGPSGPRGLPGPPGAPGPQGFQGPPGEPGEPGASGPMGPR

GPPGPPGKNGDDGEAGKPGRPGERGPPGPQGARGLPGTAGLPGMKGHRGFSGLDGAKGDA

GPAGPKGEPGSPGENGAPGQMGPRGLPGERGRPGPPGPAGARGNDGATGAAGPPGPTGPA

GPPGFPGAVGAKGEAGPQGARGSEGPQGVRGEPGPPGPAGAAGPAGNPGADGQPGGKGAN

GAPGIAGAPGFPGARGPSGPQGPSGPPGPKGNSGEPGAPGSKGDTGAKGEPGPTGVQGPP

GPAGEEGKRGARGEPGPAGLPGPPGERGGPGSRGFPGADGVAGPKGPAGERGSPGPAGPK

GSPGEAGRPGEAGLPGAKGLTGSPGSPGPDGKTGPPGPAGQDGRPGPPGPPGARGQAGVM

GFPGPKGAAGEPGKAGERGVPGPPGAVGPAGKDGEAGAQGPPGPAGPAGERGEQGPAGSP

GFQGLPGPAGPPGEAGKPGEQGVPGDLGAPGPSGARGERGFPGERGVQGPPGPAGPRGAN

GAPGNDGAKGDAGAPGAPGSQGAPGLQGMPGERGAAGLPGPKGDRGDAGPKGADGAPGKD

GVRGLTGPIGPPGPAGAPGDKGETGPSGPAGPTGARGAPGDRGEPGPPGPAGFAGPPGAD

GQPGAKGEPGDAGAKGDAGPPGPAGPTGPPGPIGSVGAPGPKGARGSAGPPGATGFPGAA

GRVGPPGPSGNAGPPGPPGPAGKEGSKGPRGETGPAGRPGEAGPPGPPGPAGEKGSPGAD

GPAGAPGTPGPQGIAGQRGVVGLPGQRGERGFPGLPGPSGEPGKQGPSGPSGERGPPGPM

GPPGLAGPPGESGREGAPGAEGSPGRDGAPGPKGDRGESGPAGPPGAPGAPGAPGPVGPA

GKSGDRGETGPAGPAGPVGPVGARGPAGPQGPRGDKGETGEQGDRGIKGHRGFSGLQGPP

GPPGSPGEQGPSGASGPAGPRGPPGSAGAPGKDGLNGLPGPIGPPGPRGRTGDAGPVGPP

GPPGPPGPPGPPSGGFDFSFLPQPPQEKAHDGGRYYRAQYD-GKGVGAGPGPMGLMGPRG

PPGAVGAPGPQGFQGPAGEPGEPGQTGPAGARGPPGPPGKAGEDGHPGKPGRPGERGVVG

PQGARGFPGTPGLPGFKGIRGHNGLDGLKGQPGAPGVKGEPGAPGENGTPGQTGARGLPG

ERGRVGAPGPAGARGNDGSVGPVDPAGPIGSAGPPGFPGAPGPKGELGPVGNPGPAGPAG

PRGEVGLPGVSGPVGPPGNPGANGLPGAKGAAGLPGVAGAPGLPGPRGIPGPAGAAGATG

ARGLVGEPGPAGSKGESGNKGEPGAAGPQGPPGPSGEEGKRGPNGEVGSAGPPGPPGLRG

NPGSRGLPGADGRAGVMGPPGSRGPTGPAGVRGPNGDSGRPGEPGLMGPRGFPGSPGNVG

PAGKEGPAGLPGIDGRPGPIGPAGARGEPGNIGFPGPKGPTGDPGKNGEKGHAGLAGARG

APGPDGNNGAQGPPGPQGVQGGKGEQGPAGPPGFQGLPGPAGTAGEVGKPGERGIPGEFG

LPGPAGPRGERGPPGESGAAGPAGPIGSRGPSGPPGPDGNKGEPGVLGAPGTAGPSGPSG

LPGERGAAGIPGGKGEKGETGLRGDVGSPGRDGARGAPGAVGAPGPAGANGDRGEAGPAG

PAGPAGPRGSPGERGEVGPAGPNGFAGPAGAAGQPGAKGERGTKGPKGENGPVGPTGPVG

AAGPAGPNGPPGPAGSRGDGGPPGATGFPGAAGRIGPPGPSGISGPPGPPGPAGKEGLRG

PRGDQGPVGRTGETGASGPPGFAGEKGPSGEPGTAGPPGTPGPQGLLGAPGFLGLPGSRG

ERGLPGVAGSVGEPGPLGIAGPPGARGPPGAVGNPGVNGAPGEAGRDGNPGSDGPPGRDG

QAGHKGERGYPGNPGPAGAAGAPGPQGAVGPAGKHGNRGEPGPAGSVGPAGAVGPRGPSG

PQGIRGEKGEPGDKGPRGLPGLKGHNGLQGLPGLAGHHGDQGAPGPVGPAGPRGPAGPSG

PAGKDGRTGQPGAVGPAGIRGSQGSQGPAGPPGPPGPPGPPGPSGGGYDFGYEGDFYRA

>Equus

QLSYGYDEKSAG--ISVPGPMGPSGPRGLPGPPGAPGPQGFQGPPGEPGEPGASGPMGPR

GPPGPPGKNGDDGEAGKPGRPGERGPPGPQGARGLPGTAGLPGMKGHRGFSGLDGAKGDA

GPAGPKGEPGSPGENGAPGQMGPRGLPGERGRPGAPGPAGARGNDGATGAAGPPGPTGPA

GPPGFPGAVGAKGEAGPQGARGSEGPQGVRGEPGPPGPAGAAGPAGNPGADGQPGAKGAN

GAPGIAGAPGFPGARGPSGPQGPSGPPGPKGNSGEPGAPGNKGDTGAKGEPGPTGIQGPP

GPAGEEGKRGARGEPGPTGLPGPPGERGGPGARGFPGADGVAGPKGPAGERGAPGPAGPK

GSPGEAGRPGEAGLPGAKGLTGSPGSPGPDGKTGPPGPAGQDGRPGPPGPPGARGQAGVM

GFPGPKGAAGEPGKAGERGVPGPPGAVGPAGKDGEAGAQGPPGPAGPAGERGEQGPAGSP

GFQGLPGPAGPPGESGKPGEQGVPGDLGAPGPSGARGERGFPGERGVQGPPGPAGPRGSN

GAPGNDGAKGDAGAPGAPGSQGAPGLQGMPGERGAAGLPGPKGDRGDAGPKGADGSPGKD

GVRGLTGPIGPPGPAGAPGDKGETGPSGPAGPTGARGAPGDRGEPGPPGPAGFAGPPGAD

GQPGAKGEPGDAGAKGDAGPPGPAGPAGPPGPIGSVGAPGPKGARGSAGPPGATGFPGAA

GRVGPPGPSGNAGPPGPPGPVGKEGGKGPRGETGPAGRPGEAGPPGPPGPAGEKGSPGAD

GPAGAPGTPGPQGIAGQRGVVGLPGQRGERGFPGLPGPSGEPGKQGPSGASGERGPPGPV

GPPGLAGPPGESGREGSPGAEGSPGRDGSPGPKGDRGETGPAGPPGAPGAPGAPGPVGPA

GKSGDRGEAGPAGPAGPIGPVGARGPAGPQGPRGDKGETGEQGDRGIKGHRGFSGLQGPP

GPPGSPGEQGPSGASGPAGPRGPPGSAGAPGKDGLNGLPGPIGPPGPRGRTGDAGPVGPP

GPPGPPGPPGPPSGGFDFSFLPQPPQEKSHDGGRYYRAQFD-AKG-G-GPGPMGLMGPRG

PPGASGAPGPQGFQGPAGEPGEPGQTGPAGARGPPGPPGKAGEDGHPGKPGRPGERGVVG

PQGARGFPGTPGLPGFKGIRGHNGLDGLKGQPGAPGVKGEPGAPGENGTPGQAGARGLPG

ERGRVGAPGPAGARGSDGSVGPVGPAGPIGSAGPPGFPGAPGPKGELGPVGNPGPAGPAG

PRGEVGLPGLSGPVGPPGNPGANGLTGAKGAAGLPGVAGAPGLPGPRGIPGPAGAAGATG

ARGLVGEPGPAGSKGESGNKGEPGAAGPQGPPGPSGEEGKRGPNGEPGSTGPAGPPGLRG

SPGSRGLPGADGRAGVMGPAGSRGASGPAGVRGPNGDSGRPGEPGLMGPRGFPGSPGNIG

PAGKEGPVGLPGIDGRPGPIGPAGARGEPGNIGFPGPKGPSGEPGKPGDKGHAGLAGARG

APGPDGNNGAQGPPGPQGVQGGKGEQGPAGPPGFQGLPGPAGTAGEVGKPGERGLPGEFG

LPGPAGARGERGPPGESGAAGPAGPIGSRGPSGPPGPDGNKGEPGVLGAPGTAGPSGPSG

LPGERGAAGIPGGKGEKGETGLRGEIGNPGRDGARGAPGAVGAPGPAGANGDRGEAGAAG

PAGPAGPRGSPGERGEVGPAGPNGFAGPAGAAGQPGAKGERGTKGPKGENGPVGPTGPVG

AAGPSGPNGPPGPAGSRGDGGPPGVTGFPGAAGRTGPPGPSGISGPPGPPGAAGKEGLRG

PRGDQGPVGRAGETGASGPPGFAGEKGPSGEPGTAGPPGTPGPQGLLGAPGILGLPGSRG

ERGLPGVAGSLGEPGPLGIAGPPGARGPPGAVGAPGVNGAPGEAGRDGNPGSDGPPGRDG

QPGHKGERGYPGNAGPVGAVGAPGPHGPVGPTGKHGHRGEPGPVGSVGPVGAVGPRGPSG

PQGVRGDKGEPGDKGPRGLPGIKGHNGLQGLPGLAGQHGDQGAPGSVGPAGPRGPAGPTG

PVGKDGRSGQPGTVGPAGVRGSQGSQGPAGPPGPPGPPGPPGPSGGGYDFGYDGDFYRA

>Vulpes

QMSYGYDEKSTGG-ISVPGPMGPSGPRGLPGPPGAPGPQGFQGPPGEPGEPGASGPMGPR

GPPGPPGKNGDDGEAGKPGRPGERGPPGPQGARGLPGTAGLPGMKGHRGFSGLDGAKGDA

GPAGPKGEPGSPGENGAPGQMGPRGLPGERGRPGAPGPAGARGNDGATGAAGPPGPTGPA

GPPGFPGAVGAKGEAGPQGARGSEGPQGVRGEPGPPGPAGAAGPAGNPGADGQPGAKGAN

GAPGIAGAPGFPGARGPSGPQGPSGPPGPKGNSGEPGAPGNKGDTGAKGEPGPTGIQGPP

GPAGEEGKRGARGEPGPTGLPGPPGERGGPGSRGFPGADGVAGPKGPAGERGSPGPAGPK

GSPGEAGRPGEAGLPGAKGLTGSPGSPGPDGKTGPPGPAGQDGRPGPPGPPGARGQAGVM

GFPGPKGAAGEPGKAGERGVPGPPGAVGPAGKDGEAGAQGPPGPAGPAGERGEQGPAGSP

GFQGLPGPAGPPGEAGKPGEQGVPGDLGAPGPSGARGERGFPGERGVQGPPGPAGPRGAN

GAPGNDGAKGDAGAPGAPGSQGAPGLQGMPGERGAAGLPGPKGDRGDAGPKGADGSPGKD

GVRGLTGPIGPPGPAGAPGDKGEAGPSGPAGPTGARGAPGDRGEPGPPGPAGFAGPPGAD

GQPGAKGEPGDAGAKGDAGPPGPAGPTGPPGPIGNVGAPGPKGARGSAGPPGATGFPGAA

GRVGPPGPSGNAGPPGPPGPAGKEGGKGPRGETGPAGRPGEVGPPGPPGPAGEKGSPGAD

GPAGAPGTPGPQGIAGQRGVVGLPGQRGERGFPGLPGPSGEPGKQGPSGASGERGPPGPM

GPPGLAGPPGESGREGSPGAEGSPGRDGSPGPKGDRGETGPAGPPGAPGAPGAPGPVGPA

GKNGDRGETGPAGPAGPIGPVGARGPAGPQGPRGDKGETGEQGDRGIKGHRGFSGLQGPP

GPPGSPGEQGPSGASGPAGPRGPPGSAGSPGKDGLNGLPGPIGPPGPRGRTGDAGPVGPP

GPPGPPGPPGPPSGGFDFSFLPQPPQEKAHDGGRYYRAQYD-GKGVGLGPGPMGLMGPRG

PPGASGAPGPQGFQGPAGEPGEPGQTGPAGARGPPGPPGKAGEDGHPGKPGRPGERGVVG

PQGARGFPGTPGLPGFKGIRGHNGLDGLKGQPGAPGVKGEPGAPGENGTPGQTGARGLPG

ERGRVGAPGPAGARGSDGSVGPVGPAGPIGSAGPPGFPGAPGPKGELGPVGNPGPAGPAG

PRGEVGLPGVSGPVGPPGNPGANGLTGAKGAAGLPGVAGAPGLPGPRGIPGPVGAAGATG

ARGLVGEPGPAGSKGESGNKGEPGSAGAQGPPGPSGEEGKRGPNGEAGSAGPSGPPGLRG

SPGSRGLPGADGRAGVMGPPGPRGATGPAGVRGPNGDSGRPGEPGLMGPRGFPGAPGNVG

PAGKEGPMGLPGIDGRPGPIGPAGARGEPGNIGFPGPKGPTGDPGKNGDKGHAGLAGARG

APGPDGNNGAQGPPGPQGVQGGKGEQGPAGPPGFQGLPGPAGTAGEVGKPGERGPPGEFG

LPGPAGPRGERGPPGESGAAGPSGPIGSRGPSGPPGPDGNKGEPGVLGAPGTAGASGPGG

LPGERGAAGIPGGKGEKGETGLRGEIGNPGRDGARGAPGAMGAPGPAGATGDRGEAGPAG

PAGPAGPRGSPGERGEVGPAGPNGFAGPAGAAGQPGAKGERGTKGPKGENGPVGPTGPIG

SAGPSGPNGPPGPAGSRGDGGPPGATGFPGAAGRTGPPGPSGITGPPGPPGAAGKEGLRG

PRGDQGPVGRTGETGASGPPGFTGEKGPSGEPGTAGPPGTPGPQGLLGAPGILGLPGSRG

ERGLPGVAGSVGEPGPLGIAGPPGARGPPGAVGAPGVNGAPGEAGRDGNPGNDGPPGRDG

QAGHKGERGYPGNIGPVGAVGAPGPHGPVGPAGKHGNRGEPGPAGSVGPAGAVGPRGPSG

PQGIRGDKGEPGEKGPRGLPGLKGHNGLQGLPGLAGQHGDQGAPGSVGPAGPRGPAGPSG

PAGKDGRTGQPGTVGPAGIRGSQGSQGPAGPPGPPGPPGPPGPSGGGYDFGYEGDFYRA

>Callorhinus

QMSYGYDEKSTGG-ISVPGPMGPSGPRGLPGPPGAPGPQGFQGPPGEPGEPGASGPMGPR

GPPGPPGKNGDDGEAGKPGRPGERGPPGPQGARGLPGTAGLPGMKGHRGFSGLDGAKGDA

GPAGPKGEPGSPGENGAPGQMGPRGLPGERGRPGAPGPAGARGNDGATGAAGPPGPTGPA

GPPGFPGAVGAKGEAGPQGARGSEGPQGVRGEPGPPGPAGAAGPAGNPGADGQPGAKGAN

GAPGIAGAPGFPGARGPSGPQGPSGPPGPKGNSGEPGAPGNKGDTGAKGEPGPTGIQGPP

GPAGEEGKRGARGEPGPTGLPGPPGERGGPGSRGFPGADGVAGPKGPAGERGSPGPAGPK

GSPGEAGRPGEAGLPGAKGLTGSPGSPGPDGKTGPPGPAGQDGRPGPPGPPGARGQAGVM

GFPGPKGAAGEPGKAGERGVPGPPGAVGPAGKDGEAGAQGAPGPAGPAGERGEQGPAGSP

GFQGLPGPAGPPGEAGKPGEQGVPGDLGAPGPSGARGERGFPGERGVQGPPGPAGPRGAN

GAPGNDGAKGDAGAPGAPGSQGAPGLQGMPGERGAAGLPGPKGDRGDAGPKGADGSPGKD

GVRGLTGPIGPPGPAGAPGDKGEAGPSGPAGPTGARGAPGDRGEPGPPGPAGFAGPPGAD

GQPGAKGEPGDAGAKGDAGPPGPAGPTGPPGPIGNVGAPGPKGARGSAGPPGATGFPGAA

GRVGPPGPSGNAGPPGPPGPAGKEGGKGPRGETGPAGRPGEVGPPGPPGPAGEKGSPGAD

GPAGAPGTPGPQGIAGQRGVVGLPGQRGERGFPGLPGPSGEPGKQGPSGASGERGPPGPM

GPPGLAGPPGESGREGSPGAEGSPGRDGSPGPKGDRGETGPSGPPGAPGAPGAPGPVGPA

GKNGDRGETGPAGPAGPIGPVGARGPAGPQGPRGDKGETGEQGDRGIKGHRGFSGLQGPP

GPPGSPGEQGPSGASGPAGPRGPPGSAGSPGKDGLNGLPGPIGPPGPRGRTGDAGPVGPP

GPPGPPGPPGPPSGGFDFSFLPQPPQEKAHDGGRYYRAQYDPGKGVGLGPGPMGLMGPRG

PPGASGAPGPQGFQGPAGEPGEPGQTGPAGARGPPGPPGKAGEDGHPGKPGRPGERGVVG

PQGARGFPGTPGLPGFKGIRGHNGLDGLKGQPGAPGVKGEPGAPGENGTPGQTGARGLPG

ERGRVGAPGPAGARGSDGSVGPVGPAGPIGSAGPPGFPGAPGPKGELGPVGNPGPAGPAG

PRGEVGLPGVSGPVGPPGNPGANGLTGAKGAAGLPGVAGAPGLPGPRGIPGPVGAAGATG

ARGLVGEPGPAGSKGEGGNKGEPGSAGPQGPPGPSGEEGKRGPNGEAGSTGPSGPPGLRG

GPGSRGLPGADGRAGVMGPPGPRGATGPAGVRGPNGDSGRPGEPGLMGPRGFPGAPGNVG

PAGKEGPMGLPGIDGRPGPIGPAGARGEPGNIGFPGPKGPTGDPGKAGEKGHAGLAGARG

APGPDGNNGAQGPPGPQGVQGGKGEQGPAGPPGFQGLPGPSGTAGEAGKPGERGLPGEFG

LPGPAGPRGERGPPGESGAAGPSGPIGSRGPSGPPGPDGNKGEPGVLGAPGTAGPSGPGG

LPGERGAAGIPGGKGEKGETGLRGEVGNPGRDGARGAPGAVGAPGPAGATGDRGEAGTAG

PAGPAGPRGSPGERGEVGAAGPNGFAGPAGAAGQPGAKGERGTKGPKGENGPVGPTGPVG

SAGPSGPNGPPGPAGSRGDGGPPGATGFPGAAGRTGPPGPSGITGPPGPPGAAGKEGLRG

PRGDQGPVGRTGETGAHGPPGFAGEKGPSGEPGTAGPPGTAGPQGLLGAPGILGLPGSRG

ERGLPGVSGSVGEPGPLGIAGPSGARGPPGAVGAPGVNGAPGEAGRDGNPGNDGPPGRDG

QPGHKGERGYPGNIGPVGAVGAPGPHGPVGPTGKHGNRGEPGPAGSVGPVGAAGPRGPSG

PQGVRGDKGEPGDKGPRGLPGLKGHNGLQGLPGLAGQHGDQGAPGSVGPAGPRGPAGPSG

PAGKDGRTGHPGTVGPAGVRGSQGSQGPAGPPGPPGPPGPPGPSGGGYDFGYEGDFYRA

>Enhydra

QMSYGYDEKSTGG-ISVPGPMGPSGPRGLPGPPGAPGPQGFQGPPGEPGEPGASGPMGPR

GPPGPPGKNGDDGEAGKPGRPGERGPPGPQGARGLPGTAGLPGMKGHRGFSGLDGAKGDA

GPAGPKGEPGSPGENGAPGQMGPRGLPGERGRPGAPGPAGARGNDGATGAAGPPGPTGPA

GPPGFPGAVGAKGEAGPQGARGSEGPQGVRGEPGPPGPAGAAGPAGNPGADGQPGAKGAN

GAPGIAGAPGFPGARGPSGPQGPSGPPGPKGNSGEPGAPGNKGDTGAKGEPGPTGIQGPP

GPAGEEGKRGARGEPGPTGLPGPPGERGGPGSRGFPGADGVAGPKGPAGERGSPGPAGPK

GSPGEAGRPGEAGLPGAKGLTGSPGSPGPDGKTGPPGPAGQDGRPGPPGPPGARGQAGVM

GFPGPKGAAGEPGKAGERGVPGPPGAVGPAGKDGEAGAQGAPGPAGPAGERGEQGPAGSP

GFQGLPGPAGPPGEAGKPGEQGVPGDLGAPGPSGARGERGFPGERGVQGPPGPAGPRGAN

GAPGNDGAKGDAGAPGAPGSQGAPGLQGMPGERGAAGLPGPKGDRGDAGPKGADGSPGKD

GVRGLTGPIGPPGPAGAPGDKGEAGPSGPAGPTGARGAPGDRGEPGPPGPAGFAGPPGAD

GQPGAKGEPGDAGAKGDAGPPGPAGPTGPPGPIGNVGAPGPKGARGSAGPPGATGFPGAA

GRVGPPGPSGNAGPPGPPGPAGKEGGKGPRGETGPAGRPGEVGPPGPPGPAGEKGSPGAD

GPAGAPGTPGPQGIAGQRGVVGLPGQRGERGFPGLPGPSGEPGKQGPSGASGERGPPGPM

GPPGLAGPPGESGREGSPGAEGSPGRDGSPGPKGDRGETGPAGPPGAPGAPGAPGPVGPA

GKNGDRGETGPAGPAGPIGPVGARGPTGPQGPRGDKGETGEQGDRGIKGHRGFSGLQGPP

GPPGSPGEQGPSGASGPAGPRGPPGSAGSPGKDGLNGLPGPIGPPGPRGRTGDAGPVGPP

GPPGPPGPPGPPSGGFDFSFLPQPPQEKAHDGGRYYRAQYDPGKGVGLGPGPMGLMGPRG

PPGASGAPGPQGFQGPAGEPGEPGQTGPAGARGPPGPPGKAGEDGHPGKPGRPGERGVVG

PQGARGFPGTPGLPGFKGIRGHNGLDGLKGQPGAPGVKGEPGAPGENGTPGQTGARGLPG

ERGRVGAPGPAGARGSDGSVGPVGPAGPIGSAGPPGFPGAPGPKGELGPVGNPGPAGPAG

PRGEVGLPGVSGPVGPPGNPGANGLTGAKGAAGLPGVAGAPGLPGPRGIPGPVGAAGATG

ARGLVGEPGPAGSKGESGNKGEPGSAGPQGPPGPSGEEGKRGPNGEAGSTGPSGPPGLRG

SPGSRGLPGADGRAGVMGPPGPRGATGPAGVRGPNGDSGRPGEPGLMGPRGFPGAPGNIG

PAGKEGPMGLPGIDGRPGPIGPAGARGEPGNIGFPGPKGPTGDPGKPGEKGHAGLAGARG

APGPDGNNGAQGPPGPQGVQGGKGEQGPAGPPGFQGLPGPAGTAGEVGKPGERGLPGEFG

LPGPAGPRGERGPPGESGAAGPSGPIGSRGPSGPPGPDGNKGEPGVLGAPGTAGPSGPGG

LPGERGSAGVPGGKGEKGETGLRGEVGNPGRDGARGAPGAVGAPGPAGATGDRGEAGPAG

PSGPAGPRGSPGERGEVGPAGPNGFAGPAGAAGQPGAKGERGTKGPKGENGPVGPTGPVG

SAGPSGPNGPPGPAGSRGDGGPPGATGFPGAAGRTGPPGPSGITGPPGPPGAAGKEGLRG

PRGDQGPVGRTGETGAHGPPGFAGEKGPSGEPGTAGPPGTSGPQGLLGAPGILGLPGSRG

ERGLPGVSGSVGEPGPLGIAGPPGARGPPGAVGAPGVNGAPGEAGRDGNPGNDGPPGRDG

QPGHKGERGYPGNIGSVGAVGAPGPHGPVGPTGKHGNRGEPGPAGSVGPVGAAGPRGPSG

PQGVRGDKGEPGDKGPRGLPGLKGHNGLQGLPGLAGQHGDQGAPGSLGPAGPRGPAGPSG

PAGKDGRTGHPGTVGPAGIRGSQGSQGPAGPPGPPGPPGPPGPSGGGYDFGYEGDFYRA

>Felis

QMSYGYDEKSTGG-ISVPGPMGPSGPRGLPGPPGAPGPQGFQGPPGEPGEPGASGPMGPR

GPPGPPGKNGDDGEAGKPGRPGERGPPGPQGARGLPGTAGLPGMKGHRGFSGLDGAKGDA

GPAGPKGEPGSPGENGAPGQMGPRGLPGERGRPGAPGPAGARGNDGATGAAGPPGPTGPA

GPPGFPGAVGAKGEAGPQGARGSEGPQGVRGEPGPPGPAGAAGPAGNPGADGQPGAKGAN

GAPGIAGAPGFPGARGPSGPQGPSGPPGPKGNSGEPGAPGNKGDTGAKGEPGPTGIQGPP

GPAGEEGKRGARGEPGPTGLPGPPGERGGPGSRGFPGADGVAGPKGPAGERGSPGPAGPK

GSPGEAGRPGEAGLPGAKGLTGSPGSPGPDGKTGPPGPAGQDGRPGPPGPPGARGQAGVM

GFPGPKGAAGEPGKAGERGVPGPPGAVGPAGKDGEAGAQGPPGPAGPAGERGEQGPAGSP

GFQGLPGPAGPPGEAGKPGEQGVPGDLGAPGPSGARGERGFPGERGVQGPPGPAGPRGAN

GAPGNDGAKGDAGAPGAPGSQGAPGLQGMPGERGAAGLPGPKGDRGDAGPKGADGSPGKD

GVRGLTGPIGPPGPAGAPGDKGEAGPSGPAGPTGARGAPGDRGEPGPPGPAGFAGPPGAD

GQPGAKGEPGDAGAKGDAGPPGPAGPTGPPGPIGNVGAPGPKGARGSAGPPGATGFPGAA

GRVGPPGPSGNAGPPGPPGPVGKEGGKGPRGETGPAGRPGEVGPPGPPGPAGEKGSPGAD

GPAGAPGTPGPQGIAGQRGVVGLPGQRGERGFPGLPGPSGEPGKQGPSGPSGERGPPGPM

GPPGLAGPPGESGREGSPGAEGSPGRDGSPGPKGDRGETGPAGPPGAPGAPGAPGPVGPA

GKSGDRGETGPAGPAGPIGPVGARGPAGPQGPRGDKGETGEQGDRGIKGHRGFSGLQGPP

GPPGSPGEQGPSGASGPAGPRGPPGAAGSPGKDGLNGLPGPIGPPGPRGRTGDAGPVGPP

GPPGPPGPPGPPSGGFDFSFLPQPPQEKAHDGGRYYRAQYDPGKGVGLGPGPMGLMGPRG

PPGASGAPGPQGFQGPAGEPGEPGQTGPAGARGPPGPPGKAGEDGHPGKPGRPGERGVVG

PQGARGFPGTPGLPGFKGIRGHNGLDGLKGQPGAPGVKGEPGAPGENGTPGQTGARGLPG

ERGRVGAPGPAGARGSDGSVGPVGPAGPIGSAGPPGFPGAPGPKGELGPVGNPGPAGPAG

PRGEMGLPGVSGPVGPPGNPGANGLTGAKGAAGLPGVAGAPGLPGPRGIPGPVGAAGATG

ARGLVGEPGPAGSKGESGNKGEPGSAGPQGPPGPSGEEGKRGPNGEAGSAGPSGPPGLRG

SPGSRGLPGADGRAGVMGPPGPRGATGPAGVRGPNGDAGRPGEPGLMGPRGFPGAPGNVG

PAGKEGPMGLPGIDGRPGPIGPAGARGEPGNIGFPGPKGPTGDPGKNGDKGHAGLAGARG

APGPDGNNGAQGPPGPQGVQGGKGEQGPAGPPGFQGLPGPAGTAGEVGKPGERGLPGEFG

LPGPAGPRGERGPPGESGAAGPSGPIGSRGPSGPPGPDGNKGEPGVLGAPGTAGPSGPSG

LPGERGAAGIPGGKGEKGETGLRGEIGNPGRDGARGAPGAVGAPGPAGATGDRGEAGPAG

PAGPAGPRGSPGERGEVGPAGPNGFAGPAGAAGQPGAKGERGTKGPKGENGPVGPTGPVG

SAGPSGPNGPPGPAGSRGDGGPPGATGFPGAAGRTGPPGPSGITGPPGPPGAAGKEGLRG

PRGDQGPVGRTGETGASGPPGFAGEKGPSGEPGTAGPPGTPGPQGLLGAPGILGLPGSRG

ERGLPGVSGSVGEPGPLGISGPPGARGPSGAVGAPGVNGAPGEAGRDGNPGNDGPPGRDG

QPGHKGERGYPGNIGPVGAVGAPGPHGPVGPTGKHGNRGEPGPAGVVGPVGAVGPRGPTG

PQGIRGDKGEPGDKGPRGLPGLKGHNGLQGLPGLAGQHGDQGAPGSVGPAGPRGPAGPSG

PMGKDGRTGHPGSVGPAGVRGSQGSQGPAGPPGPPGPPGPPGPSGGGYDFGYEGDFYRA

>Canis

QMSYGYDEKSTGG-ISVPGPMGPSGPRGLPGPPGAPGPQGFQGPPGEPGEPGASGPMGPR

GPPGPPGKNGDDGEAGKPGRPGERGPPGPQGARGLPGTAGLPGMKGHRGFSGLDGAKGDA

GPAGPKGEPGSPGENGAPGQMGPRGLPGERGRPGAPGPAGARGNDGATGAAGPPGPTGPA

GPPGFPGAVGAKGEAGPQGARGSEGPQGVRGEPGPPGPAGAAGPAGNPGADGQPGAKGAN

GAPGIAGAPGFPGARGPSGPQGPSGPPGPKGNSGEPGAPGNKGDTGAKGEPGPTGIQGPP

GPAGEEGKRGARGEPGPTGLPGPPGERGGPGSRGFPGADGVAGPKGPAGERGSPGPAGPK

GSPGEAGRPGEAGLPGAKGLTGSPGSPGPDGKTGPPGPAGQDGRPGPPGPPGARGQAGVM

GFPGPKGAAGEPGKAGERGVPGPPGAVGPAGKDGEAGAQGPPGPAGPAGERGEQGPAGSP

GFQGLPGPAGPPGEAGKPGEQGVPGDLGAPGPSGARGERGFPGERGVQGPPGPAGPRGAN

GAPGNDGAKGDAGAPGAPGSQGAPGLQGMPGERGAAGLPGPKGDRGDAGPKGADGSPGKD

GVRGLTGPIGPPGPAGAPGDKGEAGPSGPAGPTGARGAPGDRGEPGPPGPAGFAGPPGAD

GQPGAKGEPGDAGAKGDAGPPGPAGPTGPPGPIGNVGAPGPKGARGSAGPPGATGFPGAA

GRVGPPGPSGNAGPPGPPGPAGKEGGKGARGETGPAGRPGEVGPPGPPGPAGEKGSPGAD

GPAGAPGTPGPQGIAGQRGVVGLPGQRGERGFPGLPGPSGEPGKQGPSGTSGERGPPGPM

GPPGLAGPPGESGREGSPGAEGSPGRDGSPGPKGDRGETGPAGPPGAPGAPGAPGPVGPA

GKNGDRGETGPAGPAGPIGPVGARGPAGPQGPRGDKGETGEQGDRGIKGHRGFSGLQGPP

GPPGSPGEQGPSGASGPAGPRGPPGSAGSPGKDGLNGLPGPIGPPGPRGRTGDAGPVGPP

GPPGPPGPPGPPSGGFDFSFLPQPPQEKAHDGGRYYRAQYD-GKGVGLGPGPMGLMGPRG

PPGASGAPGPQGFQGPAGEPGEPGQTGPAGARGPPGPPGKAGEDGHPGKPGRPGERGVVG

PQGARGFPGTPGLPGFKGIRGHNGLDGLKGQPGAPGVKGEPGAPGENGTPGQTGARGLPG

ERGRVGAPGPAGARGSDGSVGPVGPAGPIGSAGPPGFPGAPGPKGELGPVGNPGPAGPAG

PRGEVGLPGVSGPVGPPGNPGANGLTGAKGAAGLPGVAGAPGLPGPRGIPGPVGAAGATG

ARGIVGEPGPAGSKGESGNKGEPGSAGAQGPPGPSGEEGKRGPNGEAGSAGPSGPPGLRG

SPGSRGLPGADGPAGVMGPPGPRGATGPAGVRGPNGDSGRPGEPGLMGPRGFPGAPGNVG

PAGKEGPMGLPGIDGRPGPIGPAGARGEPGNIGFPGPKGPTGDPGKNGDKGHAGLAGARG

APGPDGNNGAQGPPGPQGVQGGKGEQGPAGPPGFQGLPGPAGTAGEVGKPGERGLPGEFG

LPGPAGPRGERGPPGESGAAGPSGPIGSRGPSGPPGPDGNKGEPGVLGAPGTAGASGPGG

LPGERGAAGIPGGKGEKGETGLRGEIGNPGRDGARGAPGAMGAPGPAGATGDRGEAGPAG

PAGPAGPRGTPGERGEVGPAGPNGFAGPAGAAGQPGAKGERGTKGPKGENGPVGPTGPIG

SAGPSGPNGPPGPAGSRGDGGPPGATGFPGAAGRTGPPGPSGITGPPGPPGAAGKEGLRG

PRGDQGPVGRTGETGASGPPGFTGEKGPSGEPGTAGPPGTPGPQGLLGAPGILGLPGSRG

ERGLPGVAGSVGEPGPLGIAGPPGARGPPGAVGAPGVNGAPGEAGRDGNPGNDGPPGRDG

QAGHKGERGYPGNIGPVGAVGAPGPHGPVGPTGKHGNRGEPGPAGSVGPVGAVGPRGPSG

PQGIRGDKGEPGEKGPRGLPGLKGHNGLQGLPGLAGQHGDQGAPGSVGPAGPRGPAGPSG

PAGKDGRTGQPGTVGPAGIRGSQGSQGPAGPPGPPGPPGPPGPSGGGYDFGYEGDFYRA

>Suricata

QMSYGYDEKSVGG-VSVPGPMGPSGPRGLPGPPGAPGPQGFQGPPGEPGEPGASGPMGPR

GPPGPPGKNGDDGEAGKPGRPGERGPPGPQGARGLPGTAGLPGMKGHRGFSGLDGAKGDA

GPAGPKGEPGSPGENGAPGQMGPRGLPGERGRPGAPGPAGARGNDGATGAAGPPGPTGPA

GPPGFPGAVGAKGEAGPQGARGSEGPQGVRGEPGPPGPAGAAGPAGNPGADGQPGAKGAN

GAPGIAGAPGFPGARGPSGPQGPSGPPGPKGNSGEPGAPGNKGDTGAKGEPGPTGIQGPP

GPAGEEGKRGARGEPGPTGLPGPPGERGGPGSRGFPGADGVAGPKGPAGERGSPGPAGPK

GSPGEAGRPGEAGLPGAKGLTGSPGSPGPDGKTGPPGPAGQDGRPGPPGPPGARGQAGVM

GFPGPKGAAGEPGKAGERGVPGPPGAVGPAGKDGEAGAQGPPGPAGPAGERGEQGPAGSP

GFQGLPGPAGPPGEAGKPGEQGVPGDLGAPGPSGARGERGFPGERGVQGPPGPAGPRGAN

GAPGNDGAKGDAGAPGAPGSQGAPGLQGMPGERGAAGLPGPKGDRGDAGPKGADGSPGKD

GVRGLTGPIGPPGPAGAPGDKGEAGPSGPAGPTGARGAPGDRGEPGPPGPAGFAGPPGAD

GQPGAKGEPGDAGAKGDAGPPGPAGPTGPPGPIGNVGAPGPKGARGSAGPPGATGFPGAA

GRVGPPGPSGNAGPPGPPGPVGKEGGKGPRGETGPAGRPGEVGPPGPPGPAGEKGSPGAD

GPAGAPGTPGPQGIAGQRGVVGLPGQRGERGFPGLPGPSGEPGKQGPSGPSGERGPPGPM

GPPGLAGPPGESGREGSPGAEGSPGRDGSPGPKGDRGETGPAGPPGAPGAPGAPGPVGPA

GKNGDRGETGPAGPAGPIGPVGARGPAGPQGPRGDKGETGEQGDRGIKGHRGFSGLQGPP

GPPGSPGEQGPSGASGPAGPRGPPGAAGSPGKDGLNGLPGPIGPPGPRGRTGDAGPVGPP

GPPGPPGPPGPPSGGFDFSFLPQPPQEKAHDGGRYYRAQYDPGKGVGLGPGPMGLMGPRG

PPGASGAPGPQGFQGPAGEPGEPGQTGPAGARGPPGPPGKAGEDGHPGKPGRPGERGVVG

PQGARGFPGTPGLPGFKGIRGHNGLDGLKGQPGAPGVKGEPGAPGENGTPGQTGARGLPG

ERGRVGAPGPAGARGSDGSVGPVGPAGPIGSAGPPGFPGAPGPKGELGPVGNPGPAGPAG

PRGEMGLPGVSGPVGPPGNPGANGLTGAKGAAGLPGVAGAPGLPGPRGIPGPVGAAGATG

ARGLVGEPGPAGTKGESGNKGEPGSAGPQGPPGPSGEEGKRGPNGEAGSAGPSGPPGLRG

SPGSRGLPGADGRAGVMGPPGPRGATGPAGVRGPNGDAGRPGEPGLMGPRGFPGAPGNVG

PAGKEGPMGLPGIDGRPGPIGPAGARGEPGNIGFPGPKGPTGDPGKSGDKGHAGLAGARG

APGPDGNNGAQGPPGPQGVQGGKGEQGPAGPPGFQGLPGPAGTAGEVGKPGERGLSGEFG

LPGPAGPRGERGPPGESGAAGPSGPIGSRGPSGPPGPDGNKGEPGVLGAPGTAGPSGPSG

LPGERGAAGIPGGKGEKGEPGLRGEIGNPGRDGARGAPGAVGAPGPAGATGDRGEAGPAG

PAGPAGPRGSPGERGEVGPAGPNGFAGPAGAAGQPGAKGERGTKGPKGENGPVGPTGPVG

SAGPSGPNGPPGPAGSRGDGGPPGATGFPGAAGRTGPPGPSGITGPPGPPGAAGKEGLRG

PRGDQGPVGRTGETGASGPPGFAGEKGPSGEPGTAGPPGTAGPQGLLGAPGILGLPGSRG

ERGLPGVSGSVGEPGPLGISGPPGARGPPGAVGAPGVNGAPGEAGRDGNPGNDGPPGRDG

QPGHKGERGYPGNIGPVGAVGAPGPHGPVGPTGKHGNRGEPGPAGVVGPVGAVGPRGPTG

PQGTRGDKGEPGDKGPRGLPGLKGHNGLQGLPGLAGQHGDQGAPGSVGPAGPRGPAGPTG

PIGKDGRTGHPGSVGPAGVRGSQGSQGPAGPPGPPGPPGPPGPSGGGYDFGYEGDFYRA

>Ailuropoda

QMSYGYDEKSTGG-ISVPGPMGPSGPRGLPGPPGAPGPQGFQGPPGEPGEPGASGPMGPR

GPPGPPGKNGDDGEAGKPGRPGERGPPGPQGARGLPGTAGLPGMKGHRGFSGLDGAKGDA

GPAGPKGEPGSPGENGAPGQMGPRGLPGERGRPGAPGPAGARGNDGATGAAGPPGPTGPA

GPPGFPGAVGAKGEAGPQGARGSEGPQGVRGEPGPPGPAGAAGPAGNPGADGQPGAKGAN

GAPGIAGAPGFPGARGPSGPQGPSGPPGPKGNSGEPGAPGNKGDTGAKGEPGPTGIQGPP

GPAGEEGKRGARGEPGPTGLPGPPGERGGPGSRGFPGADGVAGPKGPAGERGSPGPAGPK

GSPGEAGRPGEAGLPGAKGLTGSPGSPGPDGKTGPPGPAGQDGRPGPPGPPGARGQAGVM

GFPGPKGAAGEPGKAGERGVPGPPGAVGPAGKDGEAGAQGPPGPAGPAGERGEQGPAGSP

GFQGLPGPAGPPGEAGKPGEQGVPGDLGAPGPSGARGERGFPGERGVQGPPGPAGPRGAN

GAPGNDGAKGDAGAPGAPGSQGAPGLQGMPGERGAAGLPGPKGDRGDAGPKGADGSPGKD

GVRGLTGPIGPPGPAGAPGDKGEAGPSGPAGPTGARGAPGDRGEPGPPGPAGFAGPPGAD

GQPGAKGEPGDAGAKGDAGPPGPAGPTGPPGPIGNVGAPGPKGARGSAGPPGATGFPGAA

GRVGPPGPSGNAGPPGPPGPAGKEGGKGPRGETGPAGRPGEVGPPGPPGPAGEKGSPGAD

GPAGAPGTPGPQGIAGQRGVVGLPGQRGERGFPGLPGPSGEPGKQGPSGASGERGPPGPM

GPPGLAGPPGESGREGSPGAEGSPGRDGSPGPKGDRGETGPAGPPGAPGAPGAPGPVGPA

GKSGDRGETGPAGPAGPIGPVGARGPAGPQGPRGDKGETGEQGDRGIKGHRGFSGLQGPP

GPPGSPGEQGPSGASGPAGPRGPPGSAGSPGKDGLNGLPGPIGPPGPRGRTGDAGPVGPP

GPPGPPGPPGPPSGGFDFSFLPQPPQEKAHDGGRYYRAQYDPGKGVGLGPGPMGLMGPRG

PPGASGAPGPQGFQGPAGEPGEPGQTGPAGARGPPGPPGKAGEDGHPGKPGRPGERGVVG

PQGARGFPGTPGLPGFKGIRGHNGLDGLKGQPGAPGVKGEPGAPGENGTPGQTGARGLPG

ERGRVGAPGPAGARGSDGSVGPVGPAGPIGSAGPPGFPGAPGPKGELGPVGNPGPAGPAG

PRGEVGLPGVSGPVGPPGNPGANGLTGAKGAAGLPGVAGAPGLPGPRGIPGPVGAAGATG

ARGLVGEPGPAGSKGESGNKGEPGSVGPQGPPGPSGEEGKRGPNGEAGSAGPSGPPGLRG

SPGSRGLPGADGRAGVMGPPGPRGSTGPAGVRGPNGDSGRPGEPGLMGPRGFPGAPGNVG

PAGKEGPMGLPGIDGRPGPIGPAGARGEPGNIGFPGPKGPSGEPGKAGEKGHAGLAGARG

APGPDGNNGAQGPPGPQGVQGGKGEQGPAGPPGFQGLPGPAGTAGEVGKPGERGLPGEFG

LPGPAGPRGERGPPGESGAAGPSGPIGSRGPSGPPGPDGNKGEPGVLGAPGTAGPSGPGG

LPGERGAAGVPGGKGEKGETGLRGEVGNPGRDGARGAPGAVGAPGPAGATGDRGEAGPAG

PAGPAGPRGSPGERGEVGPAGPNGFAGPAGAAGQPGAKGERGTKGPKGENGPVGPTGPVG

SAGPSGPNGPPGPAGSRGDGGPPGATGFPGAAGRTGPPGPSGITGPPGPPGAAGKEGLRG

PRGDQGPVGRTGETGAHGPPGFAGEKGPSGEPGTAGPPGTAGPQGLLGAPGILGLPGSRG

ERGLPGVSGSVGEPGPLGIAGPPGARGPPGAVGAPGVNGAPGEAGRDGNPGNDGPPGRDG

QPGHKGERGYPGNIGPVGTVGAPGPHGPVGPTGKHGNRGEPGPAGAVGPVGAVGPRGPSG

PQGVRGDKGEPGDKGPRGLPGLKGHNGLQGLPGLAGQHGDQGAPGSVGPAGPRGPAGPSG

PAGKDGRTGHPGTVGPAGVRGSQGSQGPAGPPGPPGPPGPPGPSGGGYDFGYEGDFYRA

>Ursus

QMSYGYDEKSTGG-ISVPGPMGPSGPRGLPGPPGAPGPQGFQGPPGEPGEPGASGPMGPR

GPPGPPGKNGDDGEAGKPGRPGERGPPGPQGARGLPGTAGLPGMKGHRGFSGLDGAKGDA

GPAGPKGEPGSPGENGAPGQMGPRGLPGERGRPGAPGPAGARGNDGATGAAGPPGPTGPA

GPPGFPGAVGAKGEAGPQGARGSEGPQGVRGEPGPPGPAGAAGPAGNPGADGQPGAKGAN

GAPGIAGAPGFPGARGPSGPQGPSGPPGPKGNSGEPGAPGNKGDTGAKGEPGPTGIQGPP

GPAGEEGKRGARGEPGPTGLPGPPGERGGPGSRGFPGADGVAGPKGPAGERGSPGPAGPK

GSPGEAGRPGEAGLPGAKGLTGSPGSPGPDGKTGPPGPAGQDGRPGPPGPPGARGQAGVM

GFPGPKGAAGEPGKAGERGVPGPPGAVGPAGKDGEAGAQGPPGPAGPAGERGEQGPAGSP

GFQGLPGPAGPPGEAGKPGEQGVPGDLGAPGPSGARGERGFPGERGVQGPPGPAGPRGAN

GAPGNDGAKGDAGAPGAPGSQGAPGLQGMPGERGAAGLPGPKGDRGDAGPKGADGSPGKD

GVRGLTGPIGPPGPAGAPGDKGEAGPSGPAGPTGARGAPGDRGEPGPPGPAGFAGPPGAD

GQPGAKGEPGDAGAKGDAGPPGPAGPTGPPGPIGNVGAPGPKGARGSAGPPGATGFPGAA

GRVGPxXxxGNAGPPGPPGPAGKEGGKGPRGETGPAGRPGEVGPPGPPGPAGEKGSPGAD

GPAGAPGTPGPQGIAGQRGVVGLPGQRGERGFPGLPGPSGEPGKQGPSGASGERGPPGPM

GPPGLAGPPGESGREGSPGAEGSPGRDGSPGPKGDRGETGPAGPPGAPGAPGAPGPVGPA

GKSGDRGETGPAGPAGPIGPVGARGPAGPQGPRGDKGETGEQGDRGIKGHRGFSGLQGPP

GPPGSPGEQGPSGASGPAGPRGPPGSAGSPGKDGLNGLPGPIGPPGPRGRTGDAGPVGPP

GPPGPPGPPGPPSGGYDFSFLPQPPQEKAHDGGRYYRAQYDPGKGVGLGPGPMGLMGPRG

PPGASGAPGPQGFQGPAGEPGEPGQTGPAGARGPPGPPGKAGEDGHPGKPGRPGERGVVG

PQGARGFPGTPGLPGFKGIRGHNGLDGLKGQPGAPGVKGEPGAPGENGTPGQTGARGLPG

ERGRVGAPGPAGARGSDGSVGPVGPAGPIGSAGPPGFPGAPGPKGELGPVGNPGPAGPAG

PRGEVGLPGVSGPVGPPGNPGANGLTGAKGAAGLPGVAGAPGLPGPRGIPGPVGAAGATG

ARGLVGEPGPAGSKGESGNKGEPGSVGPQGPPGPSGEEGKRGPNGEAGSAGPSGPPGLRG

SPGSRGLPGADGRAGVMGPPGPRGATGPAGVRGPNGDSGRPGEPGLMGPRGFPGAPGNVG

PAGKEGPMGLPGIDGRPGPIGPAGARGEPGNIGFPGPKGPSGEPGKAGEKGHAGLAGARG

APGPDGNNGAQGPPGPQGVQGGKGEQGPAGPPGFQGLPGPAGTAGEAGKPGERGLPGEFG

LPGPAGPRGERGPPGESGAAGPSGPIGSRGPSGPPGPDGNKGEPGVLGAPGTAGPSGPGG

LPGERGAAGIPGGKGEKGETGLRGDVGNPGRDGARGAPGAVGAPGPAGATGDRGEAGPAG

PAGPAGPRGSPGERGEVGPAGPNGFAGPAGAAGQPGAKGERGTKGPKGENGPVGPTGPVG

SAGPSGPNGPPGPAGSRGDGGPPGATGFPGAAGRTGPPGPSGITGPPGPPGAAGKEGLRG

PRGDQGPVGRTGETGAHGPPGFAGEKGPSGEPGTAGPPGTAGPQGLLGAPGILGLPGSRG

ERGLPGVSGSVGEPGPLGISGPPGARGPPGAVGAPGVNGAPGEAGRDGNPGNDGPPGRDG

QPGHKGERGYPGNIGPVGAVGAPGPHGPVGPTGKHGNRGEPGPAGAVGPVGAVGPRGPSG

PQGVRGDKGEPGDKGPRGLPGLKGHNGLQGLPGLAGQHGDQGAPGSVGPAGPRGPAGPSG

PAGKDGRIGHPGTVGPAGVRGSQGSQGPAGPPGPPGPPGPPGPSGGGYDFGYEGDFYRA

>Odobenus

QMSYGYDEKSTGG-ISVPGPMGPSGPRGLPGPPGAPGPQGFQGPPGEPGEPGASGPMGPR

GPPGPPGKNGDDGEAGKPGRPGERGPPGPQGARGLPGTAGLPGMKGHRGFSGLDGAKGDA

GPAGPKGEPGSPGENGAPGQMGPRGLPGERGRPGAPGPAGARGNDGATGAAGPPGPTGPA

GPPGFPGAVGAKGETGPQGARGSEGPQGVRGEPGPPGPAGAAGPAGNPGADGQPGAKGAN

GAPGIAGAPGFPGARGPSGPQGPSGPPGPKGNSGEPGTPGNKGDTGAKGEPGPTGIQGPP

GPAGEEGKRGARGEPGPTGLPGPPGERGGPGSRGFPGADGVAGPKGPAGERGSPGPAGPK

GSPGEAGRPGEAGLPGAKGLTGSPGSPGPDGKTGPPGPAGQDGRPGPPGPPGARGQAGVM

GFPGPKGAAGEPGKAGERGVPGPPGAVGPAGKDGEAGAQGAPGPAGPAGERGEQGPAGSP

GFQGLPGPAGPPGEAGKPGEQGVPGDLGAPGPSGARGERGFPGERGVQGPPGPAGPRGAN

GAPGNDGAKGDAGAPGAPGSQGAPGLQGMPGERGAAGLPGPKGDRGDAGPKGADGSPGKD

GVRGLTGPIGPPGPAGAPGDKGEAGPSGPAGPTGARGAPGDRGEPGPPGPAGFAGPPGAD

GQPGAKGEPGDAGAKGDAGPPGPAGPTGPPGSIGNVGAPGPKGARGSAGPPGATGFPGAA

GRVGPPGPSGNAGPPGPPGPAGKEGGKGPRGETGPAGRPGEVGPPGPPGPTGEKGSPGAD

GPAGAPGTPGPQGIAGQRGVVGLPGQRGERGFPGLPGPSGEPGKQGPSGASGERGPPGPM

GPPGLAGPPGESGREGSPGAEGSPGRDGSPGPKGDRGETGPSGPPGAPGAPGAPGPVGPA

GKNGDRGETGPAGPAGPIGPVGARGPTGPQGPRGDKGETGEQGDRGIKGHRGFSGLQGPP

GPPGSPGEQGPSGASGPAGPRGPPGSAGSPGKDGLNGLPGPIGPPGPRGRTGDAGPVGPP

GPPGPPGPPGPPSGGFDFSFLPQPPQEKAHDGGRYYRAQYDPGKGVGLGPGPMGLMGPRG

PPGASGAPGPQGFQGPAGEPGEPGQTGPAGARGPPGPPGKAGEDGHPGKPGRPGERGVVG

PQGARGFPGTPGLPGFKGIRGHNGLDGLKGQPGAPGVKGEPGAPGENGTPGQTGARGLPG

ERGRVGAPGPAGARGSDGSVGPLGPAGPIGSAGPPGFPGAPGPKGELGPVGNPGPAGPAG

PRGEVGLPGVSGPVGPPGNPGANGLTGAKGAAGLPGVAGAPGLPGPRGIPGPVGAAGATG

ARGLVGEPGPAGSKGESGNKGEPGSAGPQGPPGPSGEEGKRGPNGEAGSTGPSGPPGLRG

GPGSRGLPGADGRAGVMGPPGPRGVTGPAGVRGPNGDSGRPGEPGLMGPRGFPGAPGNVG

PAGKEGPMGLPGIDGRPGPIGPAGARGEPGNIGFPGPKGPTGDPGKAGEKGHAGLAGARG

APGPDGNNGAQGPPGPQGVQGGKGEQGPAGPPGFQGLPGPAGTAGEAGKPGERGLPGEFG

LPGPAGPRGERGPPGESGAAGPSGPIGSRGPSGPPGPDGNKGEPGVLGAPGTAGPSGPGG

LPGERGAAGIPGGKGEKGETGLRGEVGNPGRDGARGAPGAVGAPGPAGATGDRGEAGTAG

AAGPAGPRGSPGERGEVGAAGPNGFAGPAGAAGQPGAKGERGTKGPKGENGPVGPTGPVG

SAGPSGPNGPPGPAGSRGDGGPPGATGFPGAAGRTGPPGPSGITGPPGPPGAAGKEGLRG

PRGDQGPVGRTGETGAHGPPGFAGEKGPSGEPGTAGPPGTTGPQGLLGSPGILGLPGSRG

ERGLPGVSGSVGEPGPLGIAGPSGARGPPGAVGAPGVNGAPGEAGRDGNPGNDGPPGRDG

QPGHKGERGYPGNIGPVGAVGAPGPHGPVGPTGKHGNRGEPGPAGSVGPVGAAGPRGPSG

PQGVRGDKGEPGDKGPRGLPGLKGHNGLQGLPGLAGQHGDQGAPGSVGPAGPRGPAGPSG

PAGKDGRTGHPGTVGPAGVRGSQGSQGPAGPPGPPGPPGPPGPSGGGYDFGYEGDFYRA

>Pongo

QLSYGYDEKSTGG-ISVPGPMGPSGPRGLPGPPGAPGPQGFQGPPGEPGEPGASGPMGPR

GPPGPPGKNGDDGEAGKPGRPGERGPPGPQGARGLPGTAGLPGMKGHRGFSGLDGAKGDA

GPAGPKGEPGSPGENGAPGQMGPRGLPGERGRPGAPGPAGARGNDGATGAAGPPGPTGPA

GPPGFPGAVGAKGEAGPQGPRGSEGPQGVRGEPGPPGPAGAAGPAGNPGADGQPGAKGAN

GAPGIAGAPGFPGARGPSGPQGPGGPPGPKGNSGEPGAPGSKGDTGAKGEPGPVGVQGPP

GPAGEEGKRGARGEPGPTGLPGPPGERGGPGSRGFPGADGVAGPKGPAGERGSPGPAGPK

GSPGEAGRPGEAGLPGAKGLTGSPGSPGPDGKTGPPGPAGQDGRPGPPGPPGARGQAGVM

GFPGPKGAAGEPGKAGERGVPGPPGAVGPAGKDGEAGAQGPPGPAGPAGERGEQGPAGSP

GFQGLPGPAGPPGEAGKPGEQGVPGDLGAPGPSGARGERGFPGERGVQGPPGPAGPRGAN

GAPGNDGAKGDAGAPGAPGSQGAPGLQGMPGERGAAGLPGPKGDRGDAGPKGADGSPGKD

GVRGLTGPIGPPGPAGAPGDKGETGPSGPAGPTGARGAPGDRGEPGPPGPAGFAGPPGAD

GQPGAKGEPGDAGAKGDAGPPGPAGPAGPPGPIGNVGAPGAKGARGSAGPPGATGFPGAA

GRVGPPGPSGNAGPPGPPGPAGKEGGKGPRGETGPAGRPGEVGPPGPPGPAGEKGSPGAD

GPAGAPGTPGPQGIAGQRGVVGLPGQRGERGFPGLPGPSGEPGKQGPSGASGERGPPGPM

GPPGLAGPPGESGREGAPGAEGSPGRDGSPGAKGDRGETGPAGPPGAPGAPGAPGPVGPA

GKSGDRGETGPAGPAGPVGPVGARGPAGPQGPRGDKGETGEQGDRGIKGHRGFSGLQGPP

GPPGSPGEQGPSGASGPAGPRGPPGSAGAPGKDGLNGLPGPIGPPGPRGRTGDAGPVGPP

GPPGPPGPPGPPSGGFDFSFLPQPPQEKAHDGGRYYRAQYD-GKGVGLGPGPMGLMGPRG

PPGAAGAPGPQGFQGPAGEPGEPGQTGPAGARGPAGPPGKAGEDGHPGKPGRPGERGVVG

PQGARGFPGTPGLPGFKGIRGHNGLDGLKGQPGAPGVKGEPGAPGENGTPGQTGARGLPG

ERGRVGAPGPAGARGSDGSVGPVGPAGPIGSAGPPGFPGAPGPKGELGAVGNAGPAGPAG

PRGEVGLPGLSGPVGPPGNPGANGLTGAKGAAGLPGVAGAPGLPGPRGIPGPVGAAGATG

ARGLVGEPGPAGSKGESGNKGEPGSAGPQGPPGPSGEEGKRGPNGEAGSAGPPGPPGLRG

SPGSRGLPGADGRAGVMGPPGSRGASGPAGVRGPSGDAGRPGEPGLMGPRGLPGSPGNIG

PAGKEGPVGLPGIDGRPGPIGPAGARGEPGNIGFPGPKGPTGDPGKNGDKGHAGLAGARG

APGPDGNNGAQGPPGPQGVQGGKGEQGPAGPPGFQGLPGPSGPAGEVGKPGERGLHGEFG

LPGPAGPRGERGPPGESGAAGPTGPIGSRGPSGPPGPDGNKGEPGVVGAVGTAGPSGPSG

LPGERGAAGIPGGKGEKGEPGLRGEIGNPGRDGARGAPGAVGAPGPAGATGDRGEAGAAG

PAGPAGPRGSPGERGEVGPAGPNGFAGPAGAAGQPGAKGERGTKGPKGENGVVGPTGPVG

AAGPAGPNGPPGPAGSRGDGGPPGMTGFPGAAGRTGPPGPSGISGPPGPPGPAGKEGLRG

PRGDQGPVGRTGEVGAVGPPGFAGEKGPSGEAGTAGPPGTPGPQGLLGAPGILGLPGSRG

ERGLPGVAGAVGEPGPLGIAGPPGARGPPGAVGSPGVNGAPGEAGRDGNPGNDGPPGRDG

QPGHKGERGYPGNIGPVGAAGAPGPHGPVGPAGKHGNRGETGPSGPVGPVGAVGPRGPSG

PQGIRGDKGEPGEKGPRGLPGLKGHNGLQGLPGIAGHHGDQGAPGSVGPAGPRGPAGPSG

PAGKDGRTGHPGTVGPAGIRGPQGHQGPAGPPGPPGPPGPPGVSGGGYDFGYDGDFYRA

>Gorilla

QLSYGYDEKSAGG-ISVPGPMGPSGPRGLPGPPGAPGPQGFQGPPGEPGEPGASGPMGPR

GPPGPPGKNGDDGEAGKPGRPGERGPPGPQGARGLPGTAGLPGMKGHRGFSGLDGAKGDA

GPAGPKGEPGSPGENGAPGQMGPRGLPGERGRPGAPGPAGARGNDGATGAAGPPGPTGPA

GPPGFPGAVGAKGEAGPQGPRGSEGPQGVRGEPGPPGPAGAAGPAGNPGADGQPGAKGAN

GAPGIAGAPGFPGARGPSGPQGPGGPPGPKGNSGEPGAPGSKGDTGAKGEPGPVGVQGPP

GPAGEEGKRGARGEPGPTGLPGPPGERGGPGSRGFPGADGVAGPKGPAGERGSPGPAGPK

GSPGEAGRPGEAGLPGAKGLTGSPGSPGPDGKTGPPGPAGQDGRPGPPGPPGARGQAGVM

GFPGPKGAAGEPGKAGERGVPGPPGAVGPAGKDGEAGAQGPPGPAGPAGERGEQGPAGSP

GFQGLPGPAGPPGEAGKPGEQGVPGDLGAPGPSGARGERGFPGERGVQGPPGPAGPRGAN

GAPGNDGAKGDAGAPGAPGSQGAPGLQGMPGERGAAGLPGPKGDRGDAGPKGADGSPGKD

GVRGLTGPIGPPGPAGAPGDKGESGPSGPAGPTGARGAPGDRGEPGPPGPAGFAGPPGAD

GQPGAKGEPGDAGAKGDAGPPGPAGPAGPPGPIGNVGAPGAKGARGSAGPPGATGFPGAA

GRVGPPGPSGNAGPPGPPGPAGKEGGKGPRGETGPAGRPGEVGPPGPPGPAGEKGSPGAD

GPAGAPGTPGPQGIAGQRGVVGLPGQRGERGFPGLPGPSGEPGKQGPSGASGERGPPGPM

GPPGLAGPPGESGREGAPGAEGSPGRDGSPGAKGDRGETGPAGPPGAPGAPGAPGPVGPA

GKSGDRGETGPAGPAGPVGPVGARGPAGPQGPRGDKGETGEQGDRGIKGHRGFSGLQGPP

GPPGSPGEQGPSGASGPAGPRGPPGSAGAPGKDGLNGLPGPIGPPGPRGRTGDAGPVGPP

GPPGPPGPPGPPSGGFDFSFLPQPPQEKAHDGGRYYRAQYD-GKGVGLGPGPMGLMGPRG

PPGAAGAPGPQGFQGPAGEPGEPGQTGPAGARGPAGPPGKAGEDGHPGKPGRPGERGVVG

PQGARGFPGTPGLPGFKGIRGHNGLDGLKGQPGAPGVKGEPGAPGENGTPGQTGARGLPG

ERGRVGAPGPAGARGSDGSVGPVGPAGPIGSAGPPGFPGAPGPKGELGAVGNAGPAGPSG

PRGEVGLPGLSGPVGPPGNPGANGLTGAKGAAGLPGVAGAPGLPGPRGIPGPVGAAGATG

ARGLVGEPGPAGSKGESGNKGEPGSAGPQGPPGPSGEEGKRGPNGEAGSAGPPGPPGLRG

SPGSRGLPGADGRAGVMGPPGSRGASGPAGVRGPNGDAGRPGEPGLMGPRGLPGSPGNIG

PAGKEGPVGLPGIDGRPGPIGPAGARGEPGNIGFPGPKGPTGDPGKNGDKGHAGLAGARG

APGPDGNNGAQGPPGPQGVQGGKGEQGPAGPPGFQGLPGPSGPAGEVGKPGERGLHGEFG

LPGPAGPRGERGPPGESGAAGPTGPIGSRGPSGPPGPDGNKGEPGVVGAVGTAGPSGPSG

LPGERGAAGIPGGKGEKGEPGLRGEIGNPGRDGARGAPGAVGAPGPAGATGDRGEAGAAG

PAGPAGPRGSPGERGEVGPAGPNGFAGPAGAAGQPGAKGERGAKGPKGENGVVGPTGPVG

AAGPAGPNGPPGPAGSRGDGGPPGMTGFPGAAGRTGPPGPSGISGPPGPPGPAGKEGLRG

PRGDQGPVGRTGEVGAVGPPGFAGEKGPSGEAGTAGPPGTPGPQGLLGAPGILGLPGSRG

ERGLPGVAGAVGESGPLGIAGPPGARGPPGAVGSPGVNGAPGEAGRDGNPGNDGPPGRDG

QPGHKGERGYPGNIGPVGAAGAPGPHGPVGPAGKHGNRGETGPSGPVGPAGAVGPRGPSG

PQGIRGDKGEPGEKGPRGLPGLKGHNGLQGLPGIAGHHGDQGASGSVGPAGPRGPAGPSG

PAGKDGRTGHPGTVGPAGIRGPQGHQGPAGPPGPPGPPGPPGVSGGGYDFGYDGDFYRA

>Homo

QLSYGYDEKSTGG-ISVPGPMGPSGPRGLPGPPGAPGPQGFQGPPGEPGEPGASGPMGPR

GPPGPPGKNGDDGEAGKPGRPGERGPPGPQGARGLPGTAGLPGMKGHRGFSGLDGAKGDA

GPAGPKGEPGSPGENGAPGQMGPRGLPGERGRPGAPGPAGARGNDGATGAAGPPGPTGPA

GPPGFPGAVGAKGEAGPQGPRGSEGPQGVRGEPGPPGPAGAAGPAGNPGADGQPGAKGAN

GAPGIAGAPGFPGARGPSGPQGPGGPPGPKGNSGEPGAPGSKGDTGAKGEPGPVGVQGPP

GPAGEEGKRGARGEPGPTGLPGPPGERGGPGSRGFPGADGVAGPKGPAGERGSPGPAGPK

GSPGEAGRPGEAGLPGAKGLTGSPGSPGPDGKTGPPGPAGQDGRPGPPGPPGARGQAGVM

GFPGPKGAAGEPGKAGERGVPGPPGAVGPAGKDGEAGAQGPPGPAGPAGERGEQGPAGSP

GFQGLPGPAGPPGEAGKPGEQGVPGDLGAPGPSGARGERGFPGERGVQGPPGPAGPRGAN

GAPGNDGAKGDAGAPGAPGSQGAPGLQGMPGERGAAGLPGPKGDRGDAGPKGADGSPGKD

GVRGLTGPIGPPGPAGAPGDKGESGPSGPAGPTGARGAPGDRGEPGPPGPAGFAGPPGAD

GQPGAKGEPGDAGAKGDAGPPGPAGPAGPPGPIGNVGAPGAKGARGSAGPPGATGFPGAA

GRVGPPGPSGNAGPPGPPGPAGKEGGKGPRGETGPAGRPGEVGPPGPPGPAGEKGSPGAD

GPAGAPGTPGPQGIAGQRGVVGLPGQRGERGFPGLPGPSGEPGKQGPSGASGERGPPGPM

GPPGLAGPPGESGREGAPAAEGSPGRDGSPGAKGDRGETGPAGPPGAPGAPGAPGPVGPA

GKSGDRGETGPAGPAGPVGPVGARGPAGPQGPRGDKGETGEQGDRGIKGHRGFSGLQGPP

GPPGSPGEQGPSGASGPAGPRGPPGSAGAPGKDGLNGLPGPIGPPGPRGRTGDAGPVGPP

GPPGPPGPPGPPSAGFDFSFLPQPPQEKAHDGGRYYRAQYD-GKGVGLGPGPMGLMGPRG

PPGAAGAPGPQGFQGPAGEPGEPGQTGPAGARGPAGPPGKAGEDGHPGKPGRPGERGVVG

PQGARGFPGTPGLPGFKGIRGHNGLDGLKGQPGAPGVKGEPGAPGENGTPGQTGARGLPG

ERGRVGAPGPAGARGSDGSVGPVGPAGPIGSAGPPGFPGAPGPKGELGAVGNAGPAGPAG

PRGEVGLPGLSGPVGPPGNPGANGLTGAKGAAGLPGVAGAPGLPGPRGIPGPVGAAGATG

ARGLVGEPGPAGSKGESGNKGEPGSAGPQGPPGPSGEEGKRGPNGEAGSAGPPGPPGLRG

SPGSRGLPGADGRAGVMGPPGSRGASGPAGVRGPNGDAGRPGEPGLMGPRGLPGSPGNIG

PAGKEGPVGLPGIDGRPGPIGPAGARGEPGNIGFPGPKGPTGDPGKNGDKGHAGLAGARG

APGPDGNNGAQGPPGPQGVQGGKGEQGPAGPPGFQGLPGPSGPAGEVGKPGERGLHGEFG

LPGPAGPRGERGPPGESGAAGPTGPIGSRGPSGPPGPDGNKGEPGVVGAVGTAGPSGPSG

LPGERGAAGIPGGKGEKGEPGLRGEIGNPGRDGARGAPGAVGAPGPAGATGDRGEAGAAG

PAGPAGPRGSPGERGEVGPAGPNGFAGPAGAAGQPGAKGERGAKGPKGENGVVGPTGPVG

AAGPAGPNGPPGPAGSRGDGGPPGMTGFPGAAGRTGPPGPSGISGPPGPPGPAGKEGLRG

PRGDQGPVGRTGEVGAVGPPGFAGEKGPSGEAGTAGPPGTPGPQGLLGAPGILGLPGSRG

ERGLPGVAGAVGEPGPLGIAGPPGARGPPGAVGSPGVNGAPGEAGRDGNPGNDGPPGRDG

QPGHKGERGYPGNIGPVGAAGAPGPHGPVGPAGKHGNRGETGPSGPVGPAGAVGPRGPSG

PQGIRGDKGEPGEKGPRGLPGLKGHNGLQGLPGIAGHHGDQGAPGSVGPAGPRGPAGPSG

PAGKDGRTGHPGTVGPAGIRGPQGHQGPAGPPGPPGPPGPPGVSGGGYDFGYDGDFYRA

>Pan

QLSYGYDEKSTGG-ISVPGPMGPSGPRGLPGPPGAPGPQGFQGPPGEPGEPGASGPMGPR

GPPGPPGKNGDDGEAGKPGRPGERGPPGPQGARGLPGTAGLPGMKGHRGFSGLDGAKGDA

GPAGPKGEPGSPGENGAPGQMGPRGLPGERGRPGAPGPAGARGNDGATGAAGPPGPTGPA

GPPGFPGAVGAKGEAGPQGPRGSEGPQGVRGEPGPPGPAGAAGPAGNPGADGQPGAKGAN

GAPGIAGAPGFPGARGPSGPQGPGGPPGPKGNSGEPGAPGSKGDTGAKGEPGPVGVQGPP

GPAGEEGKRGARGEPGPTGLPGPPGERGGPGSRGFPGADGVAGPKGPAGERGSPGPAGPK

GSPGEAGRPGEAGLPGAKGLTGSPGSPGPDGKTGPPGPAGQDGRPGPPGPPGARGQAGVM

GFPGPKGAAGEPGKAGERGVPGPPGAVGPAGKDGEAGAQGPPGPAGPAGERGEQGPAGSP

GFQGLPGPAGPPGEAGKPGEQGVPGDLGAPGPSGARGERGFPGERGVQGPPGPAGPRGAN

GAPGNDGAKGDAGAPGAPGSQGAPGLQGMPGERGAAGLPGPKGDRGDAGPKGADGSPGKD

GVRGLTGPIGPPGPAGAPGDKGESGPSGPAGPTGARGAPGDRGEPGPPGPAGFAGPPGAD

GQPGAKGEPGDAGAKGDAGPPGPAGPAGPPGPIGNVGAPGAKGARGSAGPPGATGFPGAA

GRVGPPGPSGNAGPPGPPGPAGKEGGKGPRGETGPAGRPGEVGPPGPPGPAGEKGSPGAD

GPAGAPGTPGPQGIAGQRGVVGLPGQRGERGFPGLPGPSGEPGKQGPSGASGERGPPGPM

GPPGLAGPPGESGREGAPGAEGSPGRDGSPGAKGDRGETGPAGPPGAPGAPGAPGPVGPA

GKSGDRGETGPAGPAGPVGPVGARGPAGPQGPRGDKGETGEQGDRGIKGHRGFSGLQGPP

GPPGSPGEQGPSGASGPAGPRGPPGSAGAPGKDGLNGLPGPIGPPGPRGRTGDAGPVGPP

GPPGPPGPPGPPSAGFDFSFLPQPPQEKAHDGGRYYRAQYD-GKGVGLGPGPMGLMGPRG

PPGAAGAPGPQGFQGPAGEPGEPGQTGPAGARGPAGPPGKAGEDGHPGKPGRPGERGVVG

PQGARGFPGTPGLPGFKGIRGHNGLDGLKGQPGAPGVKGEPGAPGENGTPGQTGARGLPG

ERGRVGAPGPAGARGSDGSVGPVGPAGPIGSAGPPGFPGAPGPKGELGAVGNAGPAGPAG

PRGEVGLPGLSGPVGPPGNPGANGLTGAKGAAGLPGVAGAPGLPGPRGIPGPVGAAGATG

ARGLVGEPGPAGSKGESGNKGEPGSAGPQGPPGPSGEEGKRGPNGEAGSAGPPGPPGLRG

SPGSRGLPGADGRAGVMGPAGSRGASGPAGVRGPNGDAGRPGEPGLMGPRGLPGSPGNIG

PAGKEGPVGLPGIDGRPGPIGPAGARGEPGNIGFPGPKGPTGDPGKNGDKGHAGLAGARG

APGPDGNNGAQGPPGPQGVQGGKGEQGPAGPPGFQGLPGPSGPTGEVGKPGERGLHGEFG

LPGPAGPRGERGPPGESGAAGPTGPIGSRGPSGPPGPDGNKGEPGVVGAVGTAGPSGPSG

LPGERGAAGIPGGKGEKGEPGLRGEIGNPGRDGARGAPGAVGAPGPAGATGDRGEAGAAG

PAGPAGPRGSPGERGEVGPAGPNGFAGPAGAAGQPGAKGERGAKGPKGENGVVGPTGPVG

AAGPAGPNGPPGPAGSRGDGGPPGMTGFPGAAGRTGPPGPSGISGPPGPPGPAGKEGLRG

PRGDQGPVGRTGEVGAVGPPGFAGEKGPSGEAGTAGPPGTPGPQGLLGAPGILGLPGSRG

ERGLPGVAGAVGEPGPLGIAGPPGARGPPGAVGSPGVNGAPGEAGRDGNPGNDGPPGRDG

QPGHKGERGYPGNIGPVGAAGAPGPHGPVGPAGKHGNRGETGPSGPVGPAGAVGPRGPSG

PQGIRGDKGEPGEKGPRGLPGLKGHNGLQGLPGIAGHHGDQGAPGSVGPAGPRGPAGPSG

PAGKDGRTGHPGTVGPAGIRGPQGHQGPAGPPGPPGPPGPPGVSGGGYDFGYDGDFYRA

>Sarcophilus

QMSYGYDEKSGGG-MSVPGPMGPSGPRGLPGPPGSPGPQGFQGPPGEPGEPGASGPMGPR

GPAGPPGKNGDDGEAGKPGRPGERGPPGPQGARGLPGTAGLPGMKGHRGFSGLDGAKGDS

GPAGPKGEPGSPGENGAPGQMGPRGLPGERGRPGPPGPAGARGNDGATGAAGPPGPTGPA

GPPGFPGAVGAKGEAGPQGSRGSEGPQGVRGEPGPPGPAGSPGPSGNPGADGQPGAKGAN

GAPGIAGAPGFPGARGPSGPQGPSGAPGPKGNSGEPGTPGNKGDPGAKGEPGPVGVQGPP

GPAGEEGKRGSRGEPGPAGLPGPAGERGGPGSRGFPGADGVAGPKGAPGERGAPGPAGPK

GSPGESGRPGEAGLPGAKGLTGSPGSPGPDGKTGPPGPAGQDGRPGPPGPPGARGQAGVM

GFPGPKGAAGEPGKAGERGVPGPPGAVGPAGKDGEAGAQGAPGPAGPAGERGEQGPAGSP

GFQGLPGPAGPPGEAGKPGEQGVPGDAGAPGPSGARGERGFPGERGVQGPPGPQGPRGAN

GAPGNDGAKGDAGAPGAPGGQGPPGLQGMPGERGAAGLPGAKGDRGDAGPKGADGAPGKD

GVRGLTGPIGPPGPAGPSGDKGESGPSGPAGPTGARGAPGERGEPGPPGPAGFAGPPGAD

GQPGAKGEPGDAGAKGDAGPPGPAGPTGAPGPAGNVGAPGPKGARGSAGPPGATGFPGAA

GRVGPPGPSGNAGPPGPPGPAGKEGGKGPRGETGPIGRPGEVGPPGPPGPSGEKGSPGAD

GPAGAPGTPGPQGIAGQRGVVGLPGQRGERGFPGLPGPSGEPGKQGPSGVSGERGPPGPA

GPPGLAGPPGESGREGSPGAEGSPGRDGAPGPKGDRGETGPAGPPGAPGAPGAPGPVGPA

GKAGDRGETGPSGPAGPAGPTGARGPAGPQGPRGDKGETGEQGDRGMKGHRGFSGLQGPP

GPPGSPGEQGPSGASGPAGPRGPPGSAGAAGKDGLNGLPGPIGPPGPRGRTGDAGPAGPP

GPPGPPGPPGPPSGGFDFSFLPQPPQEKAHDSGRYYRAQYDASKGLDMGPGPMGLMGPRG

PPGASGPPGAQGFQGPAGEPGEPGQTGPAGARGPPGPPGKSGEDGHPGKPGRPGERGIVG

PQGARGFPGTPGLPGFKGIRGHNGLDGLKGQAGAPGVKGEPGAPGENGTPGQAGARGLPG

ERGRIGGPGPAGARGSDGSVGPVGPAxxxxxxGPPGFPGAPGPKGELGPVGNPGPAGPAG

PRGELGLPGMTGPVGPAGNPGANGLTGAKGAAGLPGVAGAPGLPGPRGIPGPAGAAGASG

PRGLAGEPGPAGSKGESGNKGEPGSAGPQGPPGPNGEEGKRGPNGEPGSTGPMGPPGLRG

VPGSRGLPGADGRAGGMGPPGNRGPSGPAGARGPNGDAGRPGEPGLMGPRGLPGSPGNVG

PTGKEGPAGLPGIDGRPGPTGPAGNRGEPGNIGFPGPKGPNGDPGKAGEKGHAGLAGARG

APGPDGNNGAQGPPGPTGVQGGKGEQGPAGPPGFQGLPGPSGPAGEGGKVGERGLPGEFG

LPGPAGPRGERGPPGESGAVGPTGSIGSRGPSGPPGPDGNKGEPGVVGAPGNAGPAGSGG

VPGERGAAGVPGGKGEKGETGPRGEFGNPGRDGARGAPGAMGAPGPAGATGERGEAGPAG

PVGPTGNRGAPGDRGEAGPAGPNGFAGPPGAAGQAGAKGERGTKGPKGENGIVGPTGPVG

AAGPAGPNGPPGPVGGRGDGGPPGxxxxxxxxxxxxxxxxxxxxxxxxxxGAAGKEGPRG

PRGDQGPLGRAGETGAVGPPGFAGEKGPPGEAGASGPPGSSGPQGLLGAPGILGLPGSRG

ERGLPGVSGSLGEPGPLGISGPPGARGPPGAVGSPGVNGAPGEAGRDGNPGNDGPPGRDG

LAGHKGERGYPGNPGAVGNAGAPGPHGTVGPAGKPGNRGEPGPVGSVGPVGPFGARGPSG

PQGPRGDKGEVGDKGPRGMNGFKGHNGFQGLPGISGQHGDQGAPGSTGPAGPRGPAGPSG

PPGKDGRPGHAGAVGPAGLRGSQGSQGPAGPPGPPGLPGPPGPSGGGYDFGYEGDFYRA

>Echinops

QMSYGYDEKSGGGGISVPGPMGPSGPRGLPGPPGAPGPQGFQGPAGEPGEPGASGPMGPR

GPPGPPGKNGDDGEAGKPGRAGERGPPGPQGARGLPGTAGLPGMKGHRGFSGLDGAKGDS

GPAGPKGEPGSPGENGAPGQMGPRGLPGERGRPGAPGPAGARGNDGATGAAGPPGPTGPA

GPPGFPGAVGAKGESGPQGTRGSEGPQGVRGEPGPPGPAGAAGPAGNPGADGQPGGKGAN

GAPGIAGAPGFPGARGPSGPQGPSGAPGPKGNSGEPGAPGNKGDAGAKGEPGPTGVQGPP

GPAGEEGKRGARGEPGPSGLPGPPGERGGPGSRGFPGSDGVAGPKGPAGERGSPGPAGPK

GSPGESGRPGEAGLPGAKGLTGSPGSPGPDGKTGPPGPAGQDGRPGPPGPPGARGQAGVM

GFPGPKGAAGEPGKAGERGVPGAPGAIGAAGKDGEAGAQGPPGPSGPAGERGEQGPAGSP

GFQGLPGPAGPPGESGKPGEQGVPGDLGAPGPSGARGERGFPGERGVQGPPGPAGPRGSN

GAPGNDGAKGDAGAPGAPGSQGAPGLQGMPGERGAAGLPGPKGDRGDAGPKGADGSPGKD

GVRGLTGPIGPPGPAGSPGDKGESGPSGPAGPTGARGAPGDRGEPGPAGPAGFAGPPGAD

GQPGAKGEPGDAGAKGDAGPPGPAGPTGAPGPIGNVGAPGPKGARGSAGPPGATGFPGAA

GRVGPPGPSGNAGPPGPPGPSGKEGGKGPRGETGPAGRPGEVGPPGPPGPSGEKGSPGSD

GPAGAPGTPGPQGIGGQRGVVGLPGQRGERGFPGLPGPSGEPGKQGPSGSSGERGPPGPM

GPPGLAGPPGESGREGSPGAEGSPGRDGSAGPKGDRGETGPAGPPGAPGAPGAPGPVGPA

GKNGDRGETGPAGPAGPIGPVGARGPSGPQGARGDKGETGEQGDRGMKGHRGFSGLQGPP

GPPGSPGEQGPSGASGPAGPRGPPGSAGSPGKDGLSGLPGPIGPPGPRGRTGDAGPVGPP

GPPGPPGPPGPPSGGFDFSFMPQPPQEKAHDGGRYYRAQYD-GKGVGLGPGPMGLMGPRG

PPGANGPPGPPGFNGPAGEPGEPGQTGPAGSRGPAGPPGKAGEDGHPGKPGRSGERGVVG

PQGARGFPGTPGLPGFKGIRGHNGLDGLKGQPGAAGVKGEPGAPGENGTPGQTGARGLPG

ERGRVGGSGPAGARGSDGSVGPVGPAGPLGSAGPPGFPGAPGPKGELGPVGNPGPSGPAG

PRGEVGLPGVSGPVGPPGNPGANGLAGAKGAAGLPGVAGAPGLPGPRGIPGPAGSAGATG

ARGLVGEPGPAGSKGESGSKGEPGSAGPQGPAGPSGEEGKRGPNGEPGSAGPAGPPGLRG

SPGSRGLPGADGRAGVMGPPGNRGASGPAGSRGPSGDSGRPGEPGLMGPRGLPGSPGNVG

PAGKEGLGGLPGIDGRPGPTGPAGARGEPGNIGFPGPKGPTGDAGKPGDKGHAGLAGPRG

APGPDGNNGAQGPPGPQGVQGGKGEQGPAGPPGFQGLPGPAGPTGEVGKPGERGLHGEFG

LPGPAGPRGERGPPGQSGAAGPTGSIGSRGPSGPPGPDGNKGEPGVVGAPGTAGASGPGG

LPGERGAAGVPGGKGEKGETGLRGEIGNTGRDGARGAPGAVGAPGPAGATGDRGEAGAAG

PAGPAGPRGSPGERGEVGPAGPNGFAGPAGAAGQAGPKGERGTKGPKGENGAVGPTGPIG

SAGPSGPNGPPGPAGSRGDGGPPGMTGFPGAAGRTGPPGPSGITGPPGPPGAAGKEGLRG

PRGDQGPVGRTGETGASGPTGFTGEKGPSGEPGTAGPPGTPGPQGLLGPPGILGLPGSRG

ERGLPGVAGSLGEPGPLGISGPPGARGPPGAVGNPGVNGAPGEAGRDGNPGSDGPPGRDG

LPGHKGERGYPGNAGPVGNAGAPGPHGSVGPAGKYGNRGEPGPAGSVGPVGAVGPRGPSG

PQGPRGDKGEAGEKGPRGLTGFKGHNGLQGLPGLAGQHGDQGSPGTVGPAGPRGPAGPSG

PAGKDGRSGHPGAVGPAGVRxxxxxxxxxxxxxxxxxxxxxxxxxxxxxxxxxxxxxxx

>Chrysochloris

QMSYGYDEKSGGG-ISVPGPMGPSGPRGLPGPPGAPGPQGFQGPAGEPGEPGASGPMGPR

GPPGPPGKNGDDGEAGKPGRPGERGPPGPQGARGLPGTAGLPGMKGHRGFSGLDGAKGDS

GPAGPKGEPGSPGENGAPGQMGPRGLPGERGRPGAPGPAGARGNDGATGAAGPPGPTGPA

GPPGFPGAVGAKGESGPQGARGSEGPQGVRGEPGPPGPAGTAGPAGNPGADGQPGAKGAN

GAPGIAGAPGFPGARGPSGPQGPSGAPGPKGNSGEPGAPGNKGDAGAKGEPGPTGVQGPP

GPAGEEGKRGARGEPGPTGLPGPPGERGGPGSRGFPGSDGVAGPKGPAGERGSPGPAGPK

GSPGEAGRPGEAGLPGAKGLTGSPGSPGPDGKTGPPGPAGQDGRPGPPGPPGARGQAGVM

GFPGPKGAAGEPGKPGERGAPGSPGSVGAAGKDGEAGAQGPPGPAGPAGERGEQGPAGSP

GFQGLPGPAGPPGEAGKPGEQGVPGDLGAPGPSGARGERGFPGERGVQGPPGPAGPRGSN

GAPGNDGAKGDAGAPGAPGSQGAPGLQGMPGERGAAGLPGPKGDRGDAGPKGADGSPGKD

GVRGLTGPIGPPGPAGAPGDKGESGPSGPAGPTGARGAPGDRGESGPPGPAGFAGPPGAD

GQPGAKGEPGDAGAKGDAGPAGPAGPTGAPGPIGNVGAPGPKGARGSAGPPGATGFPGAA

GRVGPPGPSGNAGPPGPPGPSGKEGGKGPRGETGPAGRPGEVGPPGPPGPSGEKGSPGAD

GPAGAPGTPGPQGIGGQRGVVGLPGQRGERGFPGLPGPSGEPGKQGPSGSSGERGPPGPM

GPPGLAGPPGESGREGSPGAEGSPGRDGSAGPKGDRGETGPAGPPGAPGAPGAPGPVGPA

GKNGDRGETGPSGPAGPVGPAGARGPAGPQGPRGDKGETGEQGDRGMKGHRGFSGLQGPP

GPPGSPGEQGPSGASGPAGPRGPPGSAGTPGKDGLNGLPGPIGPPGPRGRTGDAGPVGPP

GPPGPPGPPGPPSGGFDFSFMPQPPQEKSHDGGRYYRAQYD-GKGVGLGPGPMGLMGPRG

PPGAVGPPGPPGFSGAVGEPGEPGQTGPAGSRGPAGPPGKAGEDGHPGKPGRPGERGVVG

PQGARGFPGTPGLPGFKGIRGHNGLDGLKGQPGAPGIKGEPGAPGENGTPGQTGARGLPG

ERGRVGGSGPAGARGSDGSVGPVGPAGPIGSAGPPGFPGAPGPKGELGPVGNPGPSGPAG

PRGEVGLPGVSGPVGPPGNPGANGLAGAKGAAGLPGVAGAPGLPGPRGIPGPVGAAGAAG

ARGLVGEPGPAGSKGESGNKGEPGSAGPQGPPGPSGEEGKRGSNGEAGSAGPAGPPGLRG

SPGSRGLPGADGRAGVMGPPGNRGASGPAGVRGPSGDSGRPGEPGLMGPRGLPGSPGNTG

PAGKEGLGGLPGIDGRPGPTGPAGARGEPGNIGFPGPKGPTGDPGKSGEKGHAGLAGPRG

APGPDGNNGAQGPPGQQGVQGGKGEQGPAGPPGFQGLPGPAGTTGEVGKPGERGLHGEFG

LPGPAGPRGERGPPGQSGAAGPTGPIGSRGPSGPPGPDGNKGEAGVVGAPGTAGASGPGG

LPGERGAAGVPGGKGEKGETGLRGEIGNTGRDGARGAPGAVGAPGPAGATGDRGEAGAAG

PAGPAGPRGSPGERGEVGPAGPNGFAGPAGAAGQPGAKGERGTKGPKGENGPVGPTGPIG

SAGPSGPNGPPGPAGSRGDGGPPGVTGFPGAAGRTGPPGPSGITGPPGPPGAAGKEGLRG

PRGDQGPVGRTGETGASGPPGFTGEKGPSGEPGTAGPPGNPGPQGLLGAPGILGLPGSRG

ERGLPGVAGSLGEPGPLGIAGPPGARGPPGAVGNPGVNGAPGEAGRDGNPGSDGPPGRDG

LPGHKGERGYPGNAGPVGALGAPGPHGSVGPSGKHGNRGEPGPAGSVGPVGAVGPRGPSG

SQGPRGDKGEPGEKGPRGLSGFKGHNGLQGLPGNAGQHGDQGSPGTVGPAGPRGPAGPSG

PAGKDGRTGHPGAVGPAGVRxxxxxxxxxxxxxxxxxxxxxxxxxxxxxxxxxxxxxxx

>Loxodonta

QLSYGYDEKSAGG-ISVPGPMGPSGPRGLPGPPGAPGPQGFQGPPGEPGEPGASGPMGPR

GPPGPPGKNGDDGEAGKPGRPGERGPPGPQGARGLPGTAGLPGMKGHRGFSGLDGAKGDA

GPAGPKGEPGSPGENGAPGQMGPRGLPGERGRPGAPGPAGARGNDGATGAAGPPGPTGPA

GPPGFPGAVGAKGEAGPQGARGSEGPQGVRGEPGPPGPAGAAGPAGNPGADGQPGAKGAN

GAPGIAGAPGFPGARGPAGPQGPSGAPGPKGNSGEPGAPGSKGDAGAKGEPGPVGIQGPP

GPAGEEGKRGARGEPGPTGLPGPPGERGGPGSRGFPGADGVAGPKGPAGERGSPGPAGPK

GSPGEAGRPGEAGLPGAKGLTGSPGSPGPDGKTGPPGPAGQDGRPGPPGPPGARGQAGVM

GFPGPKGAAGEPGKAGERGVPGPPGAVGAAGKDGEAGAQGPPGPAGPAGERGEQGPAGSP

GFQGLPGPAGPPGEAGKPGEQGVPGDLGAPGPSGARGERGFPGERGVQGPPGPAGPRGSN

GAPGNDGAKGDAGAPGAPGSQGAPGLQGMPGERGAAGLPGPKGDRGDAGPKGADGSPGKD

GPRGLTGPIGPPGPAGAPGDKGEAGPSGPAGPTGARGAPGDRGEPGPPGPAGFAGPPGAD

GQPGAKGEPGDAGAKGDAGPPGPAGPTGAPGPIGNVGAPGPKGARGSAGPPGATGFPGAA

GRVGPPGPSGNAGPPGPPGPAGKEGGKGPRGETGPAGRPGEVGPPGPPGPAGEKGSPGAD

GPAGAPGTPGPQGIGGQRGVVGLPGQRGERGFPGLPGPSGEPGKQGPSGSSGERGPPGPA

GPPGLAGPPGESGREGAPGAEGSPGRDGSPGPKGDRGETGPSGPPGAPGAPGAPGPVGPA

GKSGDRGETGPAGPAGPAGPAGVRGPAGPQGPRGDKGETGEQGDRGIKGHRGFSGLQGPP

GPPGSPGEQGPSGASGPAGPRGPPGSAGAPGKDGLNGLPGPIGPPGPRGRTGDAGPVGPP

GPPGPPGPPGPPSGAFDFSFLPQPPQEKAHDGGRYYRAQYD-AKGIGLGPGPMGLMGPRG

PPGATGPPGSPGFQGPPGEPGEPGQTGPAGSRGPAGPPGKAGEDGHPGKPGRPGERGVVG

PQGARGFPGTPGLPGFKGIRGHNGLDGLKGQPGAPGVKGEPGAPGENGTPGQIGARGLPG

ERGRVGGPGPAGARGSDGSVGPVGPAGPIGSAGPPGFPGAPGPKGELGPVGNPGPSGPAG

PRGEAGLPGVSGPVGPPGNPGANGLAGAKGAAGLPGVAGAPGLPGPRGIPGPVGAAGATG

ARGLVGEPGPAGSKGESGSKGEPGSAGPQGPPGPSGEEGKRGSSGEAGSAGPAGPPGLRG

GPGSRGLPGADGRAGVMGPPGSRGASGPAGVRGPSGDSGRPGEPGVMGPRGLPGSPGNVG

PAGKEGPAGLPGIDGRPGPIGPAGARGEPGNIGFPGPKGPAGDPGKNGDKGHAGLAGPRG

APGPDGNNGAQGPPGLQGVQGGKGEQGPAGPPGFQGLPGPSGTAGEAGKPGERGLPGEFG

LPGPAGPRGERGPPGQSGAAGPTGPIGSRGPSGPPGPDGNKGEPGVVGAPGTAGPSGPGG

LPGERGAAGIPGGKGEKGETGLRGDTGNTGRDGARGAPGAVGAPGPAGATGDRGEAGPAG

SAGPAGPRGSPGERGEVGPAGPNGFAGPAGAAGQAGAKGERGTKGPKGENGPVGPTGPVG

AAGPAGPNGPPGPAGSRGDGGPPGATGFPGAAGRTGPPGPAGITGPPGPPGAAGKEGLRG

PRGDQGPVGRTGETGASGPPGFAGEKGSSGEPGTAGPPGTPGPQGLLGPPGILGLPGSRG

ERGLPGVAGAVGEPGPLGIAGPPGARGPPGAVGSPGVNGAPGEAGRDGNPGSDGPPGRDG

LPGHKGERGYPGNAGPVGTAGAPGPQGPLGPAGKHGNRGEPGPAGSVGPVGAVGPRGPSG

PQGARGDKGEAGDKGPRGLPGFKGHNGLQGLPGLAGQHGDQGSPGSVGPAGPRGPAGPSG

PVGKDGRPGHAGAVGPAGVRGSQGSQGPSGPPGPPGPPGPPGPSGGGYDFGYDGDFYRA

>Microcebus

QMSYGYDEKSTG--ISVPGPMGPSGPRGLPGPPGAPGPQGFQGPPGEPGEPGASGPMGPR

GPPGPPGKNGDDGEAGKPGRPGERGPPGPQGARGLPGTAGLPGMKGHRGFSGLDGAKGDA

GPAGPKGEPGSPGENGAPGQMGPRGLPGERGRPGASGPAGARGNDGATGAAGPPGPTGPA

GPPGFPGAVGAKGEAGPQGARGSEGPQGVRGEPGPPGPAGAAGPAGNPGADGQPGAKGAN

GAPGIAGAPGFPGARGPSGPQGPSGPPGPKGNSGEPGAPGNKGDAGAKGEPGPAGVQGPP

GPAGEEGKRGARGEPGPAGLPGPPGERGGPGSRGFPGADGVAGPKGPAGERGSPGPAGPK

GAPGEAGRPGEAGLPGAKGLTGSPGSPGPDGKTGPPGPAGQDGRPGPPGPPGARGQAGVM

GFPGPKGAAGEPGKAGERGVPGPPGAVGPAGKDGEAGAQGAPGPAGPAGERGEQGPAGSP

GFQGLPGPAGPPGESGKPGEQGVPGDLGAPGPSGARGERGFPGERGVQGPPGPAGPRGSN

GAPGNDGAKGDAGAPGAPGSQGAPGLQGMPGERGAAGLPGPKGDRGDAGPKGADGSPGKD

GVRGLTGPIGPPGPAGAPGDKGETGPSGPAGPTGARGAPGDRGEPGPPGPAGFAGPPGAD

GQPGAKGEPGDAGAKGDAGPPGPAGPAGPPGPIGNVGAPGPKGARGSAGPPGATGFPGAA

GRVGPPGPSGNAGPPGPPGPAGKEGSKGLRGETGPAGRPGEVGPPGPPGPAGEKGSPGAD

GPAGAPGTPGPQGIAGQRGVVGLPGQRGERGFPGLPGPSGEPGKQGPSGASGERGPPGPM

GPPGLAGPPGESGREGAPGAEGSPGRDGAPGPKGDRGETGPSGPPGAPGAPGAPGPVGPA

GKSGDRGETGPAGPAGPIGPAGARGPAGPQGPRGDKGETGEQGDRGIKGHRGFSGLQGPP

GPPGSPGEQGPSGASGPAGPRGPPGSAGAAGKDGLNGLPGPIGPPGPRGRTGDAGPVGPP

GPPGPPGPPGPPSGGYDFSFLPQPPQEKA-DSGRYYRAQYD-SKGVGLGPGPMGLMGPRG

PPGAAGAPGPQGFQGPAGEPGEPGQTGPAGSRGPAGPPGKAGEDGHPGKPGRSGERGVVG

PQGARGFPGTPGLPGFKGLRGHNGPDGLKGQPGAPGVKGEPGSPGENGTPGQTGARGLPG

ERGRVGAPGPAGARGSDGSVGPVGPAGPIGSAGPPGFPGAPGPKGELGPVGNPGPAGPAG

PRGEVGLPGLSGPVGPPGNPGANGLTGAKGAAGLPGVAGAPGLPGPRGIPGPAGAAGATG

ARGLVGEPGPAGSKGEGGNKGEPGSAGPQGPPGPSGEEGKRGPNGEPGSAGPAGPPGLRG

TPGSRGLPGADGRAGVMGPPGNRGASGPAGGRGPSGDSGRPGEPGLMGPRGLPGSPGNVG

PAGKEGPVGLPGIDGRPGPIGPAGARGEPGNIGFPGPKGPTGDPGKAGDRGHAGLAGARG

APGPDGNNGAQGPPGPQGVQGGKGEQGPAGPPGFQGLPGPSGPAGELGKPGERGLPGEFG

LPGPAGARGERGPPGESGAAGPTGPIGSRGPSGPPGPDGNKGEPGAVGAPGTAGASGPGG

LPGERGAAGIPGGKGEKGESGLRGEIGNPGRDGARGAPGAVGAPGPAGATGDRGEAGAAG

PAGPAGPRGSPGERGEVGPAGPNGFAGPAGAAGQAGAKGERGAKGPKGENGGVGATGPAG

PAGPSGPNGPPGPAGGRGDGGPPGVTGFPGAAGRTGPPGPSGITGPPGPPGAAGKEGLRG

PRGDQGPVGRTGETGASGPPGFAGEKGPSGESGTAGPPGTPGPQGLLGAPGILGLPGSRG

ERGLPGVAGSVGEPGPLGISGPPGARGPPGAVGSPGVNGAPGEAGRDGNPGNDGPPGRDG

QAGHKGERGYPGNIGPVGAAGAPGPHGSVGPAGKHGNRGEPGPAGSVGPVGAVGPRGPSG

PQGVRGDKGEAGDKGPRGLPGLKGHAGLQGLPGLAGHHGDQGAPGSVGPAGPRGPAGPSG

PVGKDGRSGHPGTVGPAGIRGPQGHQGPAGPPGPPGPPGPPGAGGGGYDFGFDGDFYRA

>Otolemur

QMSYGYDEKSAG--VSVPGPMGPSGPRGLPGPPGAPGPQGFQGPPGEPGEPGSAGPMGPR

GPPGPPGKNGDDGEAGKPGRPGERGPPGPQGARGLPGTAGLPGMKGHRGFSGLDGAKGDA

GAPGPKGEPGSPGENGAPGQMGPRGLPGERGRPGPSGPAGARGNDGATGAAGPPGPTGPA

GPPGFPGAAGAKGEAGPQGARGSEGPQGVRGEPGPPGPAGAAGPAGNPGADGQPGAKGAN

GAPGIAGAPGFPGARGPSGPQGPSGPPGPKGNSGEPGAPGNKGDTGAKGEPGPAGVQGPP

GPAGEEGKRGARGEPGPTGLPGPPGERGGPGSRGFPGADGVAGPKGPAGERGSPGPAGPK

GSPGEAGRPGEAGLPGAKGLTGSPGSPGPDGKTGPPGPAGQDGRPGPPGPPGARGQAGVM

GFPGPKGAAGEPGKAGERGVPGPTGAVGAPGKDGEAGAQGPPGPAGPAGERGEQGPAGSP

GFQGLPGPAGPPGEAGKPGEQGVPGDLGAPGPSGARGERGFPGERGVQGPPGPAGPRGGN

GAPGNDGAKGDAGAPGAPGSQGAPGLQGMPGERGAAGLPGPKGDRGDAGPKGADGSPGKD

GARGLTGPIGPPGPAGAPGDKGESGPSGPAGPTGARGAPGDRGEPGPPGPAGFAGPPGAD

GQPGAKGEPGDAGAKGDAGPAGPAGPAGPPGPVGNVGAPGPKGARGSAGPPGATGFPGAA

GRVGPPGPSGNAGPPGPPGPAGKEGSKGPRGETGPAGRTGEVGPPGPPGPAGEKGSPGAD

GPAGAPGTPGPQGIAGQRGVVGLPGQRGERGFPGLPGPSGEPGKQGPSGASGERGPPGPM

GPPGLAGPPGESGREGAPGAEGSPGRDGAPGPKGDRGETGPAGPPGAPGAPGAPGPVGPA

GKSGDRGETGPSGPAGPVGPAGARGPAGPQGPRGDKGETGEQGERGIKGHRGFSGLQGPP

GPPGSPGEQGPSGASGPAGPRGPPGSAGAAGKDGLNGLPGPIGPPGPRGRTGDAGPVGPA

GPPGPPGPPGPPSGGFDFSFLPQPPQEKAQDSGRYYRAQYD-GKAAGLGPGPMGLMGPRG

PPGASGAPGPQGFQGPAGEPGEPGQTGPAGARGPAGAPGKAGEDGHPGKPGRPGERGVVG

PQGARGFPGTPGLPGFKGLRGHSGPDGLKGQAGLPGAKGEPGSPGENGTPGQTGARGLPG

ERGRVGAPGPSGARGSDGSVGPVGPAGPVGSAGPPGFPGAPGPKGELGPVGNPGPAGPAG

PRGEVGLPGLSGPVGPPGNPGANGLTGAKGAAGLPGVAGAPGLPGPRGIPGPVGAAGATG

ARGLVGEPGPAGSKGESGNKGEPGSAGPQGPPGPSGEEGKRGSNGEPGSAGPSGPPGLRG

SPGSRGLPGADGRGGVMGPPGNRGQSGPAGVRGPSGDSGRPGEPGLMGPRGLPGSPGNVG

PAGKEGPAGLPGVDGRPGPVGPAGARGEPGNIGFPGPKGPSGDPGKAGDKGHPGLAGARG

APGPDGNNGAQGPPGPQGVQGGKGEQGPAGPPGFQGLPGPSGPAGEVGKPGERGLHGEFG

LPGPAGPRGERGPPGESGAAGPSGPIGSRGPSGPPGPDGNKGEPGVVGAPGTAGPSGPSG

LPGERGAAGMPGGKGEKGETGPRGEMGTTGRDGARGAPGAVGAPGPAGATGDRGEAGAAG

PAGPAGPRGSPGERGEVGPAGPNGFAGPAGAAGQPGAKGERGAKGPKGENGAVGPAGAVG

PAGPSGPNGPPGPAGGRGDGGPPGMTGFPGAAGRTGPPGPSGMSGPPGPPGPSGKEGLRG

PRGDQGPVGRSGETGPSGPPGFAGEKGPSGEAGAAGPPGTPGPQGLLGAPGILGLPGSRG

ERGLPGVAGAVGEPGPLGVAGPPGARGPSGGVGNPGVNGAPGEAGRDGNPGNDGPPGRDG

QPGHKGERGYPGNVGPAGAVGAPGSHGPVGPAGKHGNRGEPGAVGPVGPTGAVGPRGPSG

AQGVRGDKGEPGDKGPRGLPGLKGHGGLQGLPGIAGHHGDQGAPGSVGPAGPRGPAGPSG

PVGKDGRNGHPGTVGPAGVRGPQGHQGPAGPPGPPGPPGPPGASGGGYDFGYDGDFYRA

>Callithrix

QLSYGYDEKSTGG-ISVPGPMGPSGPRGLPGPPGSPGPQGFQGPPGEPGEPGASGPMGPR

GPPGPPGKNGDDGEAGKPGRPGERGPPGPQGARGLPGTAGLPGMKGHRGFSGLDGAKGDA

GPAGPKGEPGSPGENGAPGQMGPRGLPGERGRPGPPGPAGARGNDGATGAAGPPGPTGPA

GPAGFPGAVGAKGEAGPQGPRGSEGPQGVRGEPGPPGPAGAAGPAGNPGADGQPGAKGAN

GAPGIAGAPGFPGARGPSGPQGPSGPPGPKGNSGEPGAPGSKGDTGAKGEPGPVGVQGPP

GPAGEEGKRGARGEPGPTGLPGPPGERGGPGSRGFPGADGVAGPKGPAGERGSPGPAGPK

GSPGEAGRPGEAGLPGAKGLTGSPGSPGPDGKTGPPGPAGQDGRPGPPGPPGARGQAGVM

GFPGPKGAAGEPGKAGERGVPGPPGAVGPAGKDGEAGAQGPPGPAGPAGERGEQGPAGSP

GFQGLPGPAGPPGEAGKPGEQGVPGDLGAPGPSGARGERGFPGERGVQGPPGPAGPRGAN

GAPGNDGAKGDAGAPGAPGSQGAPGLQGMPGERGAAGLPGPKGDRGDAGPKGADGSPGKD

GVRGLTGPIGPPGPAGAPGDKGETGPSGPAGPTGARGAPGDRGEPGPPGPAGFAGPPGAD

GQPGAKGEPGDAGAKGDAGPPGPAGPAGPPGPIGNVGAPGPKGARGSAGPPGATGFPGAA

GRVGPPGPSGNAGPPGPPGPAGKEGGKGPRGETGPAGRPGEVGPPGPPGPAGEKGSPGAD

GPAGAPGTPGPQGIAGQRGVVGLPGQRGERGFPGLPGPSGEPGKQGPSGTSGERGPPGPM

GPPGLAGPPGESGREGAPGAEGSPGRDGSPGPKGDRGETGPAGPPGAPGAPGAPGPVGPA

GKSGDRGETGPAGPAGPIGPVGSRGPAGPQGPRGDKGETGEQGDRGIKGHRGFSGLQGPP

GPPGSPGEQGPSGASGPAGPRGPPGSAGAPGKDGLNGLPGPIGPPGPRGRTGDAGPVGPP

GPPGPPGPPGPPSGGFDFSFLPQPPQEKAHDGGRYYRAQYD-GKGVGLGPGPMGLMGPRG

PPGAAGAPGPQGFQGPAGEPGEPGQTGPAGARGPPGPPGKAGEDGHPGKPGRPGERGVVG

PQGARGFPGTPGLPGFKGIRGHNGLDGLKGQPGAPGVKGEPGAPGENGTPGQTGARGLPG

ERGRVGAPGPAGARGSDGSVGPVGPAGPIGSAGPPGFPGAPGPKGELGAIGNPGIAGPAG

PRGEVGLPGLSGPVGPPGNPGANGLTGAKGAAGLPGVAGAPGLPGPRGIPGPVGAAGATG

ARGLVGEPGPAGSKGESGNKGEPGSAGPQGPPGPSGEEGKRGPNGEAGSAGPPGPPGLRG

SPGSRGLPGADGRAGVMGPAGSRGATGPAGVRGPNGDAGRPGEPGLMGPRGLPGSPGNIG

PAGKEGPVGLPGIDGRPGPIGPAGARGEPGSIGFPGPKGPTGDPGKNGDKGHAGLAGARG

APGPDGNNGAQGPPGPQGVQGGKGEQGPAGPPGFQGLPGPSGPAGELGKPGERGLPGEFG

LPGPAGPRGERGPPGESGAAGPTGPIGSRGPSGPPGPDGNKGEPGVVGAAGTAGPSGPSG
LPGERGAAGIPGGKGEKGEPGLRGEIGNPGRDGARGAPGAVGAPGPAGATGDRGEAGAAG
PAGPAGPRGSPGERGEVGPAGPNGFAGPAGAAGQPGAKGERGAKGPKGENGVVGPTGPVG
AAGPSGPNGPPGPAGSRGDGGPPGMTGFPGAAGRTGPPGPSGISGPPGPPGPAGKEGLRG
PRGDQGPVGRTGETGAVGPPGFAGEKGPSGEAGTAGPPGTPGPQGLLGAPGILGLPGSRG
ERGLPGVAGAVGEPGPLGIAGPPGARGPPGAVGSPGVNGAPGEAGRDGNPGNDGPPGRDG
QPGHKGERGYPGNIGPVGAAGAPGPHGPVGPAGKHGNRGETGPSGPVGPAGAVGPRGPSG
PQGIRGDKGEPGDKGPRGLPGLKGHNGLQGLPGLAGHHGDQGAPGSVGPAGPRGPAGPSG
PAGKDGRTGHPGTVGPAGIRGPQGHQGPAGPPGPPGPPGPPGVSGGGYDFGYDGDFFRA
>Dasypus
QFSYGYDEKSAGG-VSVPGPMGPSGPRGLPGPPGSPGPQGFQGPPGEPGEPGSSGPMGPR
GPPGPPGKNGDDGEAGKPGRPGERGPPGPQGARGLPGTAGLPGMKGHRGFSGLDGAKGDA
GPAGPKGEPGSPGENGAPGQMGPRGLPGERGRPGASGPAxxxxxxxxxxxxxxxxxxxxx
xxxxxxxxxxxxGEAGPQGARGSEGPQGVRGEPGPPGPAGAAGPAGNPGADGQPGAKGAN
GAPGIAGAPGFPGARGPSGPQGPSGAPGPKGNSGEPGAPGNKGDTGAKGEPGPTGIQGPP
GPAGEEGKRGARGEPGPTGLPGAPGERGGPGSRGFPGADGIAGPKGPAGERGSPGPAGPK
GSPGEAGRPGEAGLPGAKGLTGSPGSPGPDGKTGPPGPAGQDGRPGPAGPPGARGQAGVM
GFPGPKGAAxxxxxxxxxxxxxxxxxxxxxxxxxxxxxxxxxxxxxxxGERGEQGPAGAP
GFQGLPGPAGPPGEAGKPGEQGVPGDLGAPGPSGARGERGFPGERGVQGPPGPAGPRGAN
GAPGNDGAKGDAGAPGAPGSQGAPGLQGMPGERGAAGLPGPKGDRxxxxxxxxxxxxxxx
xxxxxxxxxxxxxxxxxxxxxGETGPSGPAGPTGARGAPGDRGEPGPPGPAGFAGPPGAD
GQPGAKGEPGDAGAKGDAGPPGPAGAAGPPGPMGNVGAPGPKGARGSAGPPGATGFPGAA
GRVGPPGPSGNAGPPGPPGPVGKEGGKGPRGETGPAGRPGEVGPPGPPGPSGEKGSPGAD
GPAGAPGTPGPQGIAGQRGVVGLPGQRGERGFPGLPGPSGEPGKQGPSGSSGERGPPGPM
GPPGLAGPPGEAGREGSPGAEGSPGRDGSPGPKGDRGETGPAGPPGAPGAPGAPGPVGPA
GKSGDRGETGPSGPAGPAGPAGARGPSGPQGPRGDKGETGEQGDRGIKGHRGFSGLQxxx
xxxxxxxxxxxxxxxxxxxxxGPPGSAGTPGKDGLNGLPGPIGPPGPRGRTGDAGPVGPP

GPPGPPGPPGPPSGGFDFSFLPQPPQEKGHDGGRYYRAXXXxxxxxxxxxxxxxxMGPRG

PPGASGAPGPQGFQGPAGEPGEPGQTGPAGARGPAGPPGKAGEDGHPGKPGRPGERGVVG

PQGARGFPGTPGLPGFKGIRGHNGLDGLKGQAGAPGVKGEPGAPGENGTPGQTGARGLPG

ERGRVGAPGPAGARGSDGSVGPLGPAGPLGSAGPPGFPGAPGPKGELGPVGNPGPAGPAG

PRGEQGLPGVSGPVGPPGNPGANGLTGSKGAAGLPGVAGAPGLPGPRGIPGPVGAVGATG

ARGLVGEPGPAGSKGESGNKGEPGSAGPQGPPGPSGEEGKRGANGEAGSTGPSGPPGLRG

GPGSRGLPGADGRAGVMGPAGSRGASGPAGVRGPNGDPGRPGEPGLMGPRGLPGSPGNVG

PAGKEGPVGLPGIDGRPGPVGPAGPRGEAGNIGFPGPKGPTGDPGKVGEKGHAGLAGNRG

APGPDGNNGAQGPPGPQGVQGGKGEQGPAGPPGFQGLPGPAGTTGEVGKPGERGLHGEFG

LPGPAGPRGERGPPGESGAAGPVGSIGSRGPSGPPGPDGNKGEPGVVGAPGTAGPSGSGG

LPGERGGAGIPGGKGEKGETGLRGEVGTTGRDGARGAPGAIGAPGPAGATGDRGEAGAAG

PAGPSGPRGTPGERGEVGPAGPNGFAGPAGAAGQPGAKGERGTKGPKGENGIAGPTGPVG

AAGPSGPNGAPGPAGGRGDGGPPGVTGFPGAAGRTGPPGPSGITGPPGPPGAAGKEGLRG

PRGDQGPVGRTGETGAGGPPGFAGEKGPSGEPGTAGPPGTAGPQGLLGAPGILGLPGSRG

ERGLPGVAGAVGEPGPLGISGPPGARGPSGAVGSPGVNGAPGETGRDGNPGNDGPPGRDG

LPGHKGERGYAGNAGPVGAAGAPGPHGSVGPAGKHGNRGEPGPVGPVGPVGAVGPRGPSG

PQGVRGDKGEPGEKGPRGLPGLKGHNGLQGLPGLAGQHGDQGSPGPVGPAGPRGPAGPSG

PAGKDGRTGHPGAVGPAGIRGSQGSQGPSGPAGPPGPPGPPGASGGGYDFGYEGDFYRA

>Bradypus

XXXXXXXXXXXXxXxXXXXXXXXXXXXXXXXXXXXXXXXXXXXXXXXXXXXXXX

GPPGPPGKXXXXXXXXXXXXXXXXXGPPGPQGARGLPGTAGLPGMKGHRGFSGLDGAKGDA

GPAGPKGEPGSPGENGAPGQMGPRXXXXXXGRPGASGPAGARGNDGATGAAGPPGPTGPA

GPPGFPGAVGAKGEAGPQGARGSEGPQGVRGEPGPPGPAGAAGPAGNPGADGQPGAKGAN

GAPGIAGAPGFPGARGPSGPQGPSGPPGPKGNSGEPGAPGSKXXXXXXGEPGPTGIQGPP

GPAGEEGKRGARGEPGPTGLPGPPGERGGPGSRGFPGADGVAGPKXXXXXXXXXXXXXXX

GSPGEAGRPGEAGLPGAKGLTGSPGSPGPDGKTGPPGPAGQDGRPGPPGPPGARGQAGVM

GFPGPKXXXXXXXXXXXXXGVPGPPGAVGPAGKDGEAGAQGPPGPAGPAGERXXXXXXXXX

XXXXXXXXXXXXXXXXXXXXXXXXXXXXXXXXXXXXXXXXXXXXXGVQGPPGPAGPRGSN

GAPGNDGAKGDAGAPGAPGSQGAPGLQGMPGERGAAGLPGPKGDRGDAGPKXXXXXXXXX

XXXGLTGPIGPPGPAGAPGDKGESGPSGPAGPTGARGAPGDRGEPGPPGPAGFAGPPGAD

GQPGAKXXXXXXXXXGDAGPPGPAGPTGPPGPIGNVGAPGPKXXXGSAGPPGATGFPGAA

GRVGPPGPSGNAGPPGPPGPVGKEGGKGPRGETGPAGRPGEVGPPGPPGPTGEKGSPGAD

GPAGAPGTPGPQGIAGQRGVVGLPGQRXXXGFPGLPGPSGEPGKQGPSGSSGERGPPGPV

GPPGLAGPPGESGREGSPGAEGSPGRXXXXXXXGDRGESGPAGPPGAPGAPGAPGPVGPA

GKNGDRGETGPAGPAGPAGPAGARGPAGPQGPRGDKGETGEAGDRXXXXXXGFSGLQGPA

GPPGSPGEQGPSGASGPAGPRGPPGSAGSPGKDGLNGLPGPIGPPGPRXXXXXXXXXXXX

XXXXXXXXXXXXXXXXXXXXXXXXXXXXXXXXXXXXXXXXXxXXGVGLGPGPMGLMGPRX

XXXXXXXXXXXXXXXXXXXXXXXXXXXXXXXXXXXXXXXXXXXXXXXXXXXXXXXXGVVG

PQGARGFPGTPGLPGFKGIRGYNGLDGLKGQPGAAGVKGEPGAPGENGTPGQTGARXXXX

XXXXVGAPGPAGSRGSDGSVGPVGPAGPIGSAGPPGFPGAPGPKGELGPVGNTGPSGPAG

PRGEQGLPGVSGPVGPPGNPGANGLTGAKGAAGLPGVAGAPGLPGPRGIPGPVGASGATG

ARGLVGEPGPAGSKGESGGKGEPGSAGPQGPPGSSGEEGKRGPSGESGSTGPTGPPGLRX

XXXXXGLPGADGRAGVIGPAGARGASGPAGVRGPSGDTGRPGEPGLMGARGLPGSPGNVG

PAGKEGPAGLPGIDGRPGPIGPAGARGEAGNIGFPGPKXXXXXXXXXXXXXXXXXXXXXG

APGPDGNNGAQGPPGLQGVQGGKXXXXXXXXXXXXXXXXXXXXXXXXXXXXXXGLPGEFG

LPGPAGPRGERGPPGESGAVGPSGAIGSRXXXXXXXXXXXXGEPGVVGAPGTAGPAGSGG

LPGERGAAGIPGGKXXXGETGLRGEVGTTGRDGARGAPGAVGAPGPAGATGDRXXXXXXX

XXXXXXXXXXXXXXGEVGPAGPNGFAGPAGAAGQPGAKXXXXXXGPKGENGIVGPTGPVG

SAGPAGPNGPAGPAGSRGDGGPPGVTGFPGAAGRXXXXXXXXXXXXXXXXXXXXXXXXXX

XXGDQGPLGRAGETGAGGPPGFTGEKGPSGEPGTAGPPGTAGPQGLLGAPGILGLPGSRG

ERGLPGVAGAVGEPGPLGIQGPPGARGPSGAVGGPGVNGAPGEAGRDGNPGSDGPPGRXX

XXXXXXXXGYAGNPGPVGAAGAPGPHGAVGPAGKHGNRGEPGPVGSAGPVGAIGPRGPSG

PQGIRXXXXXXXXXXXXXXXXXXXXXXXXXXXXXXXXXXXXXXXXXXXXXXXXGPSGPSG

PPGKDGRTGHPGAVGPAGIRXXXXXXXXXXXXXXXXXXXXXXXXXXXXXXXXXXXXXXXX

>Choleopus

XXXXXXXXXXXXxXxXXXXXXXXXXXXXXXXXXXXXXXXXXXXXXXXXXXXXXXXXXXXXX

GPPGPPGKNGDDGEAGKXXXXXXXGPPGPQGARGLPGTAGLPGMKGHRGFSGLDGAKGDA

GPAGPKGEPGSPGENGAPGQMGPRXXXXXXGRPGASGPAGARGNDGATGAAGPPGPTGPA

GPPGFPGAVGAKGEAGPQGARGSEGPQGVRGEPGPPGPAGAAGPAGNPGADGQPGAKGAN

GAPGIAGAPGFPGARGPSGPQGPSGPPGPKXXXXXXXXXXXXXXXXXXGEPGPTGIQGPP

GPAGEEGKRXXXGEPGPTGLPGPPGERXXXXXXGFPGADGVAGPKXXXXXXXXXXXXXXX

GSPGEAGRPGEAGLPGAKGLTGSPGSPGPDGKXXXXXXXXXXXXXXXXXXXXXXGQAGVM

GFPGPKXXXXXXXXXAGERGVPGPPGAVGPAGKDGEAGAQGPPGPAGPAGERXXXXXXXX

XXXXXXXXXXXXXXXXXXXXXXXXXXXXXXXXXXXXXXXXXXXXXGVQGPPGPAGPRGSN

GAPGNDGAKGDAGAPGAPGSQGAPGLQGMPGERGAAGLPGPKGDRGDAGPKXXXXXXXXX

XXXGLTGPIGPPGPAGAPGDKGESGPSGPAGPTGARGAPGDRGEPGPPGPAGFAGPPGAD

GQPGAKXXXXXXXXXGDAGPPGPAGPTGPPGPIGNVGAPGPKXXXGSAGPPGATGFPGAA

GRVGPPGPSGNAGPPGPPGPVGKXXXXXXXGETGPAGRPGEVGPPGPPGPTGEKGSPGAD

GPAGAPGTPGPQGIAGQRGVVGLPGQRXXXGFPGLPGPSGEPGKQGPSGSSGERGPPGPV

GPPGLAGPPGESGREGSPGAEGSPGRXXXXXXXXXXGESGPAGPPGAPGAPGAPGPVGPA

GKNGDRGETGPAGPAGPAGPAGARGPAGPQGPRGDKGETGEAGDRXXXXXXGFSGLQGPA

GPPGSPGEQGPSGASGPAGPRGPPGSAGSPGKDGLNGLPGPIGPPGPRXXXXXXXXXXXX

XXXXXXXXXXXXXXXXXXXXXXXXXXXXXXXXXXXXXXXXXxXXGVGLGPGPMGLMGPRX

XXXXXXXXXXXXXXXXXXXXXXXXXXXXXXXXGPAGPPGKXXXXXXXXXXXXXXXXXXXX

XXXXXGFPGTPGLPGFKGIRGYNGLDGLKGQPGAAGVKGEPGAPGENGTPGQTGARXXXX

XXXXVGAPGPAGSRGSDGSVGPVGPAGPIGSAGPPGFPGAPGPKGELGPVGNTGPSGPAG

PRGEQGLPGVSGPVGPPGNPGANGLTGAKGAAGLPGVAGAPGLPGPRGIPGPVGASGATG

ARGLVGEPGPAGSKGESGGKGEPGSAGPQGPPGSSGEEGKRGPSGESGSTGPTGPPGLRX

XXXXXXXXXXXXXAGVIGPAGARGASGPAGVRXXXXXXXXXXXXXXXXXXGLPGSPGNVG

PAGKEGPAGLPGIDGRPGPIGPAGARGEAGNIGFPGPKXXXXXXXXXXXXXXXXXXXXXG

APGPDGNNGAQGPPGLQGVQGGKXXXXXXXXXXXXXXXXXXXXXXXXXXXXXXGLPGEFG

LPGPAGPRGERGPPGESGAVGPSGAIGSRGPSGPPGPDGNKGEPGVVGAPGTAGPAGSGG

LPGERXXXXXXXXXXXXXXXXXXXXXXXXXXXXXXGAPGAVGAPGPAGATGDRGEAGAAG

PAGPAGPRXXXXXXGEVGPAGPNGFAGPAGAAGQPGAKGERXXXGPKGENGIVGPTGPVG

SAGPAGPNGPAGPAGSRGDGGPPGVTGFPGAAGRTGPPGPSGITGPPGPSGAAGKXXXXX

XXGDQGPLGRAGETGAGGPPGFTGEKXXXXXXXXXXXXXXXXXXXXXXXXXXXXXXXXXX

XXGLPGVAGAVGEPGPLGIQGPPGARGPSGAVGGPGVNGAPGEAGRDGNPGSDGPPGRXX

XXXXXXXXGYAGNPGPVGAAGAPGPHGAVGPAGKHGNRGEPGPVGSAGPVGAIGPRGPSG

PQGIRXXXGEAGDKGPRGLPGLKGHNGLQGLPGLAGQHGDQGAPGSVGPAGPRGPSGPSG

PPGKDGRTGHPGAVGPAGIRXXXXXXXXXXXXXXXXXXXXXXXXXXXXXXXXXXXXXXX
