# Supplementary material for: Collagen Sequence Analysis Reveals Evolutionary History of Extinct West Indies Nesophontes (Island-Shrews)
Source: Mol Biol Evol. 2020 Jun 4;37(10):2931–43. doi: 10.1093/molbev/msaa137 (PMC7530613; doi:10.1093/molbev/msaa137)
Supplement: msaa137_supplementary_data [file msaa137_supplementary_data.zip › Nesophontes_TableS7.pdf]

Node	Hard minimum constraint	Soft maximum constraint	Source
Theria	156.3	169.6	Benton *et al.* 2015
Placentalia	61.6	164.6	Benton *et al.* 2016
Xenarthra	47.6	164.6	Benton *et al.* 2017
Afrotheria	56	164.6	Benton *et al.* 2018
Hominidae	11.6	33.9	Benton *et al.* 2019
Anthropoidea	24.44	34	Benton *et al.* 2020
Euarchontoglires	61.6	164.6	Benton *et al.* 2021
Lagomorpha	47.6	66	Benton *et al.* 2022
Rodentia	56	66	Benton *et al.* 2023
Laurasiatheria	61.6	164.6	Benton *et al.* 2024
Chiroptera	45	58.9	Phillips 2015
Carnivora	37.3	66	Benton *et al.* 2026

Benton MJ , *et al.* 2015. Constraints on the timescale of animal evolutionary history . *Palaeontol Electron.* 18: 1 – 106 .
Phillips MJ 2015. Four mammal fossil calibrations: balancing competing palaeontological and molecular considerations . *Palaeontol Electron.* 18: 1 – 16 .
