# Supplementary figures and images for: Collagen Sequence Analysis Reveals Evolutionary History of Extinct West Indies Nesophontes (Island-Shrews)
Source: Mol Biol Evol. 2020 Jun 4;37(10):2931–43. doi: 10.1093/molbev/msaa137 (PMC7530613; doi:10.1093/molbev/msaa137)

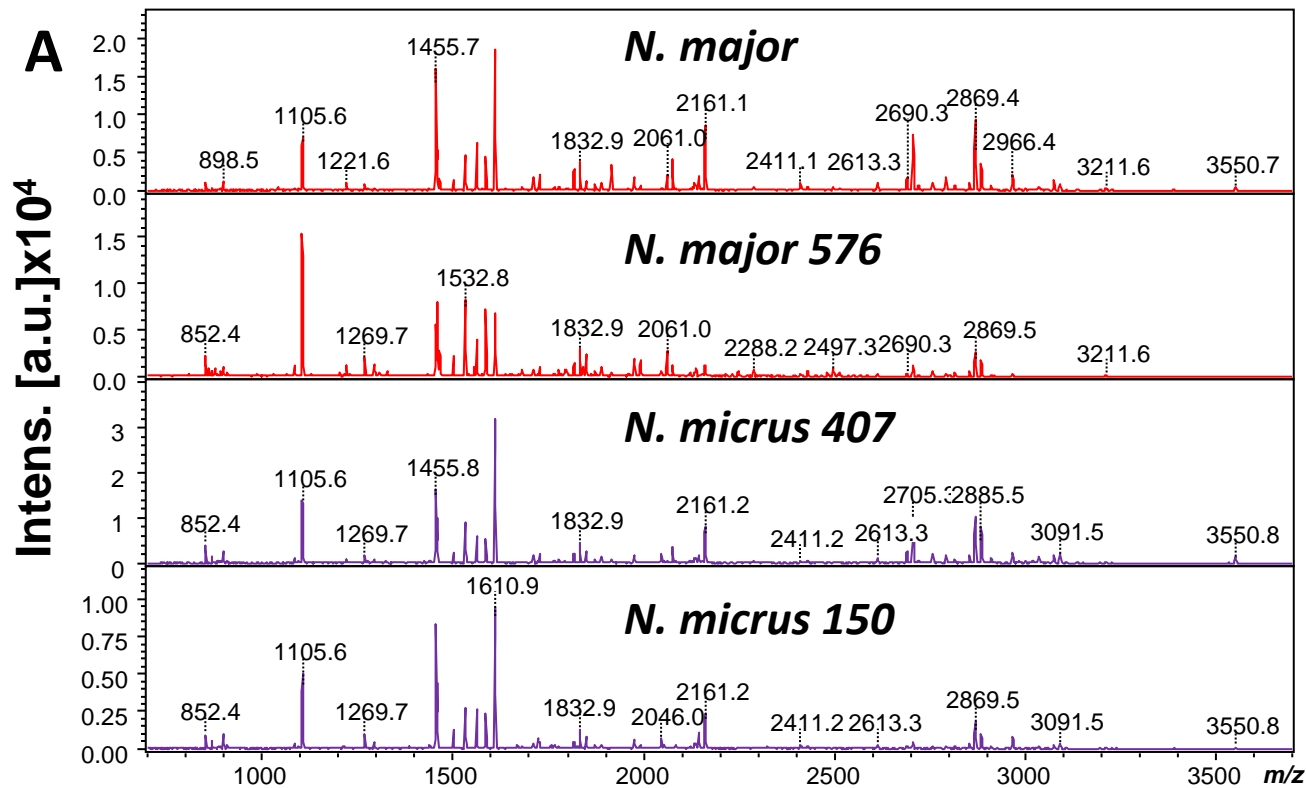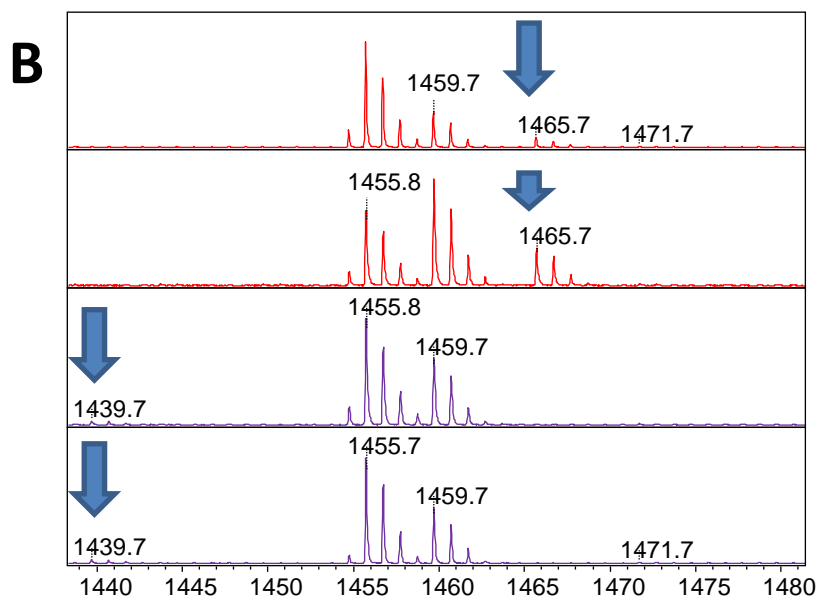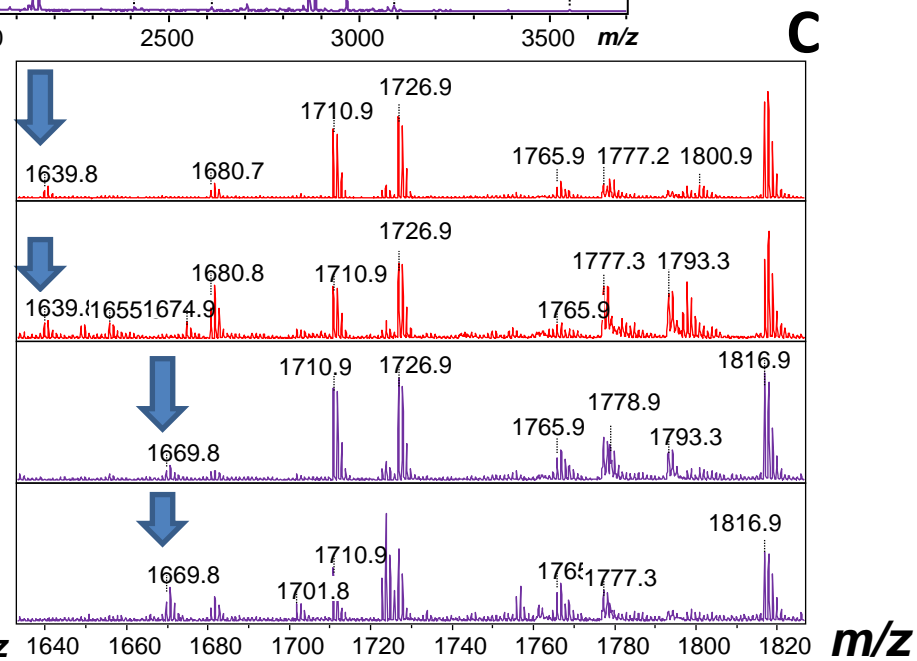

Supplement: msaa137_supplementary_data [file msaa137_supplementary_data.zip › fig_S1.pdf]

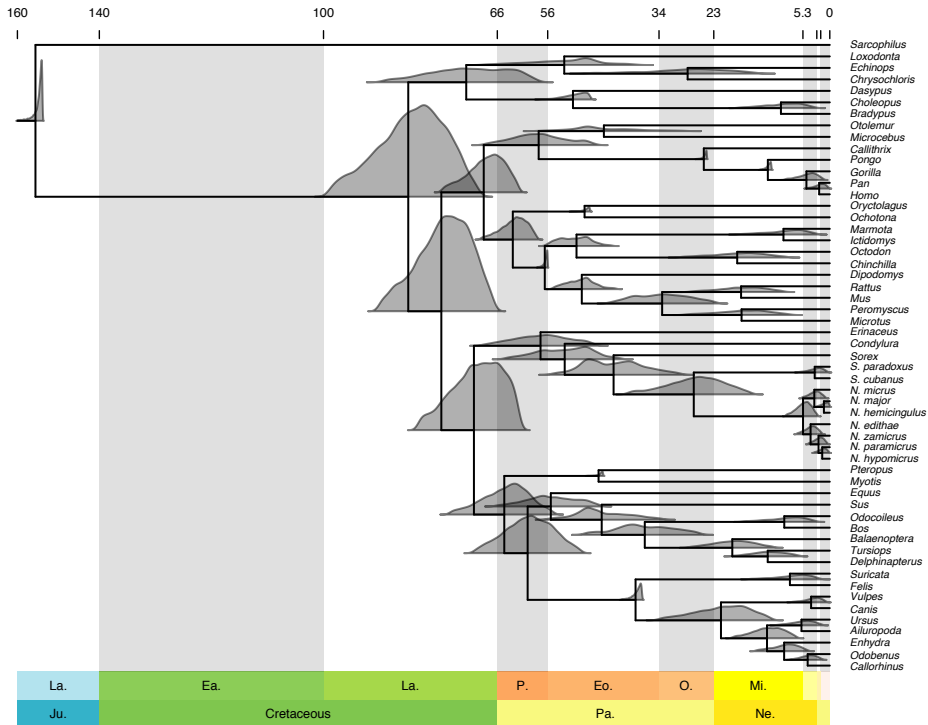

Supplement: msaa137_supplementary_data [file msaa137_supplementary_data.zip › fig_S10.pdf]

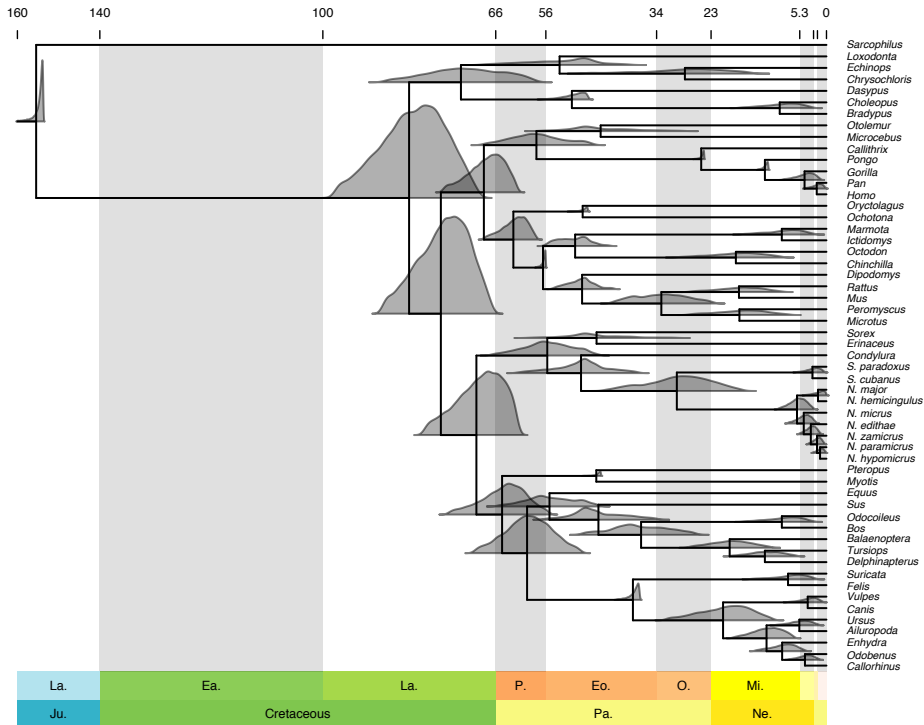

Supplement: msaa137_supplementary_data [file msaa137_supplementary_data.zip › fig_S11.pdf]

Intens. [a.u.]

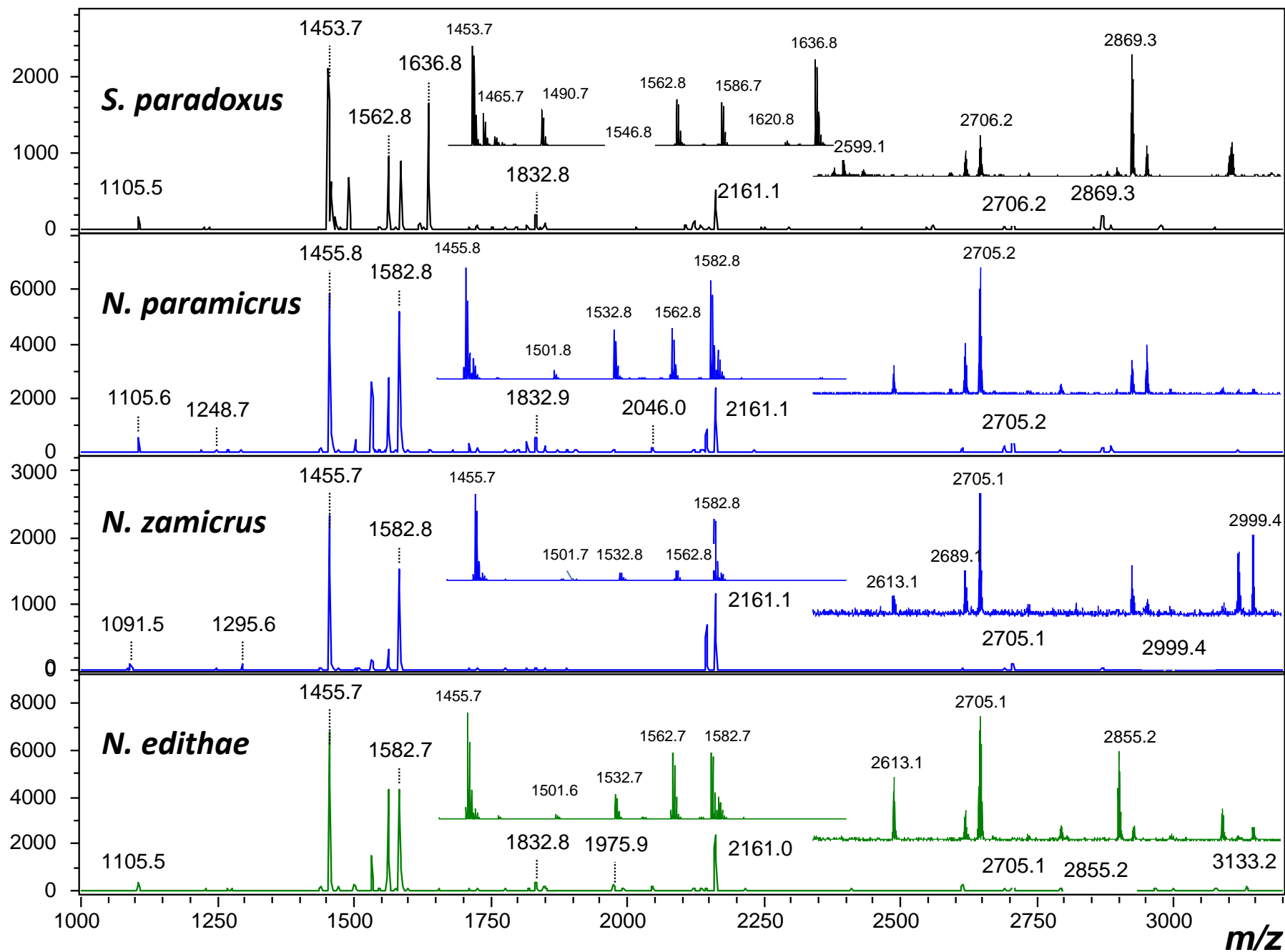

Supplement: msaa137_supplementary_data [file msaa137_supplementary_data.zip › fig_S2.pdf]

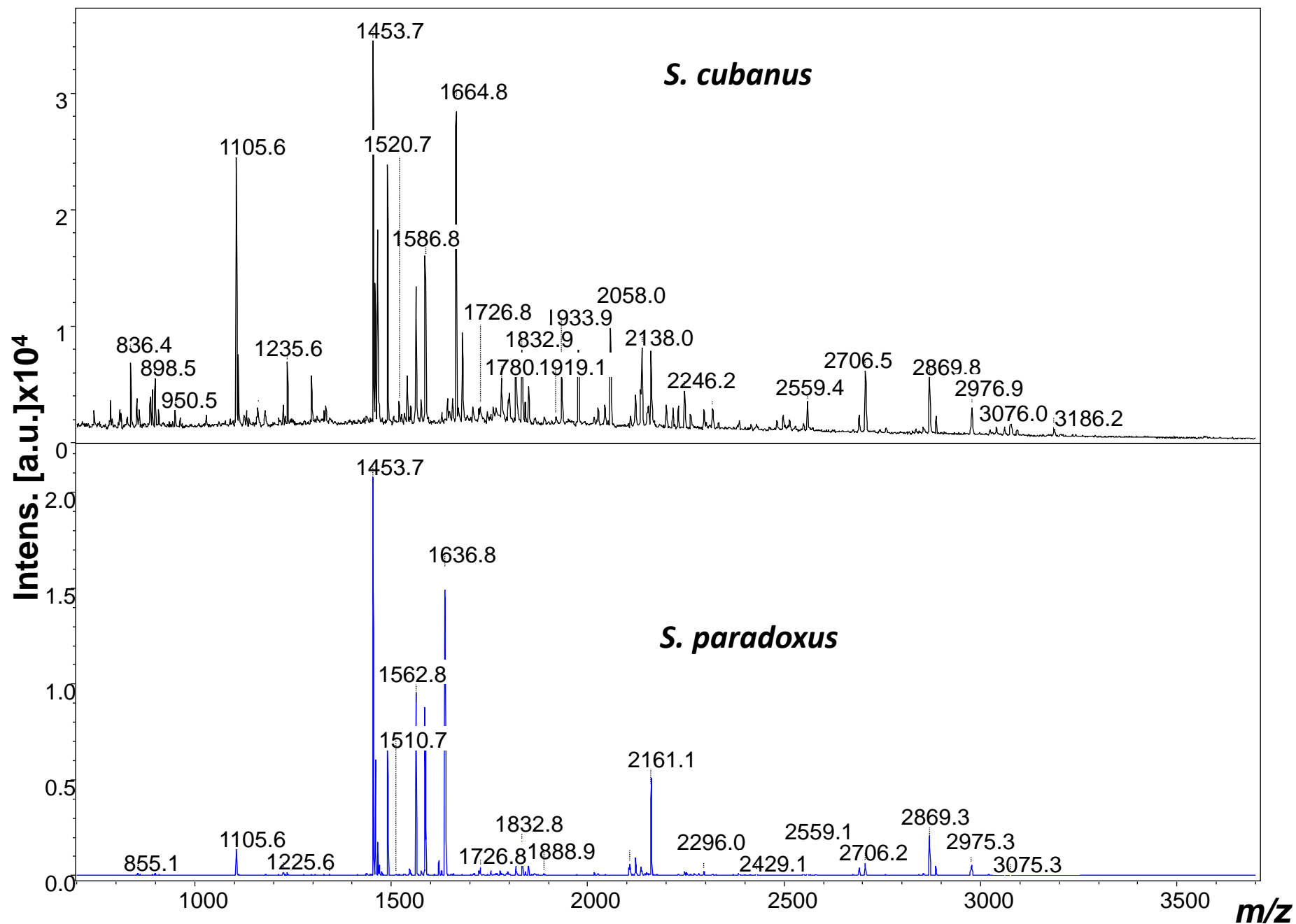

Supplement: msaa137_supplementary_data [file msaa137_supplementary_data.zip › fig_S3.pdf]

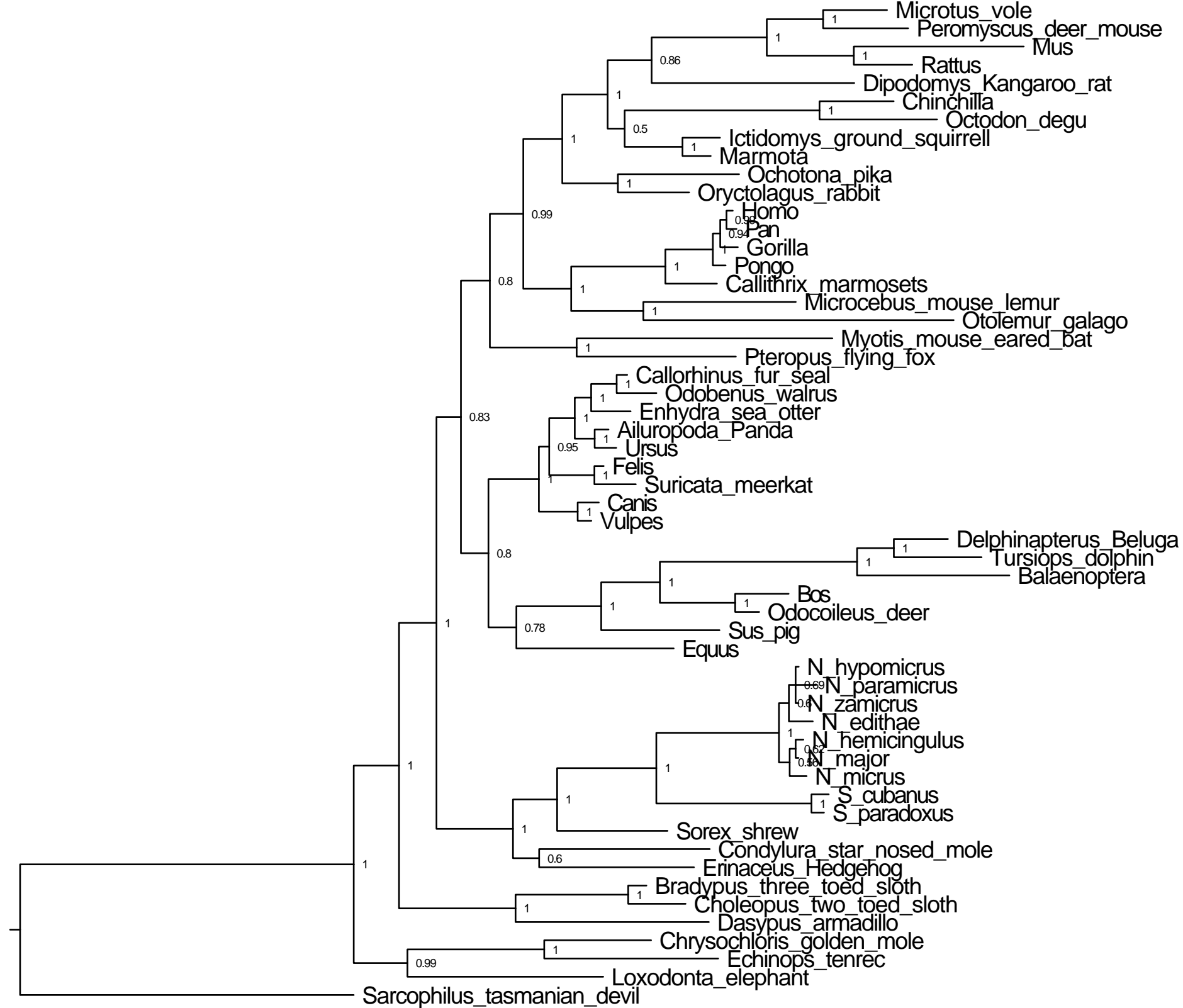

0.02

Supplement: msaa137_supplementary_data [file msaa137_supplementary_data.zip › fig_S4.pdf]

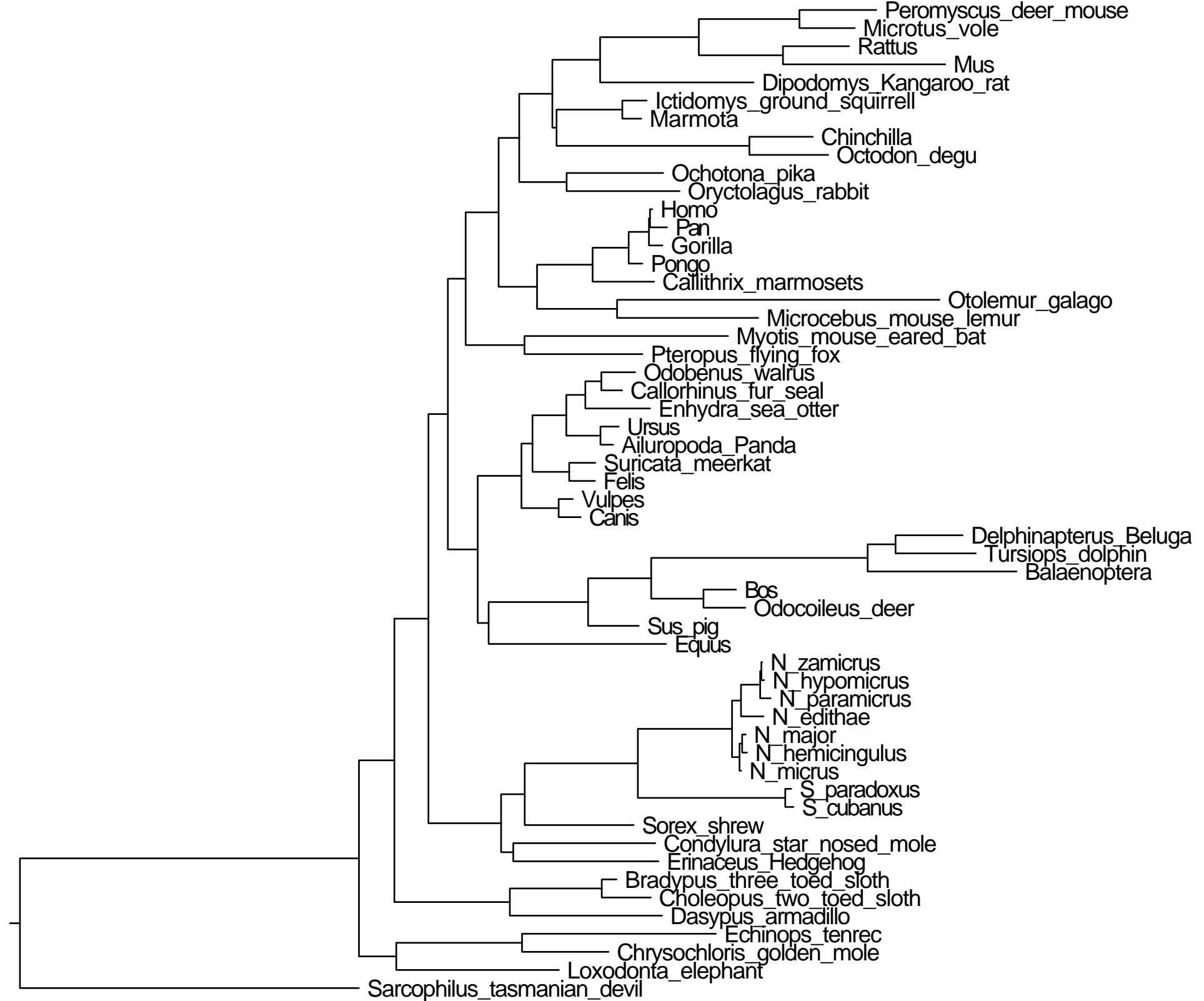

0.02

Supplement: msaa137_supplementary_data [file msaa137_supplementary_data.zip › fig_S5.pdf]

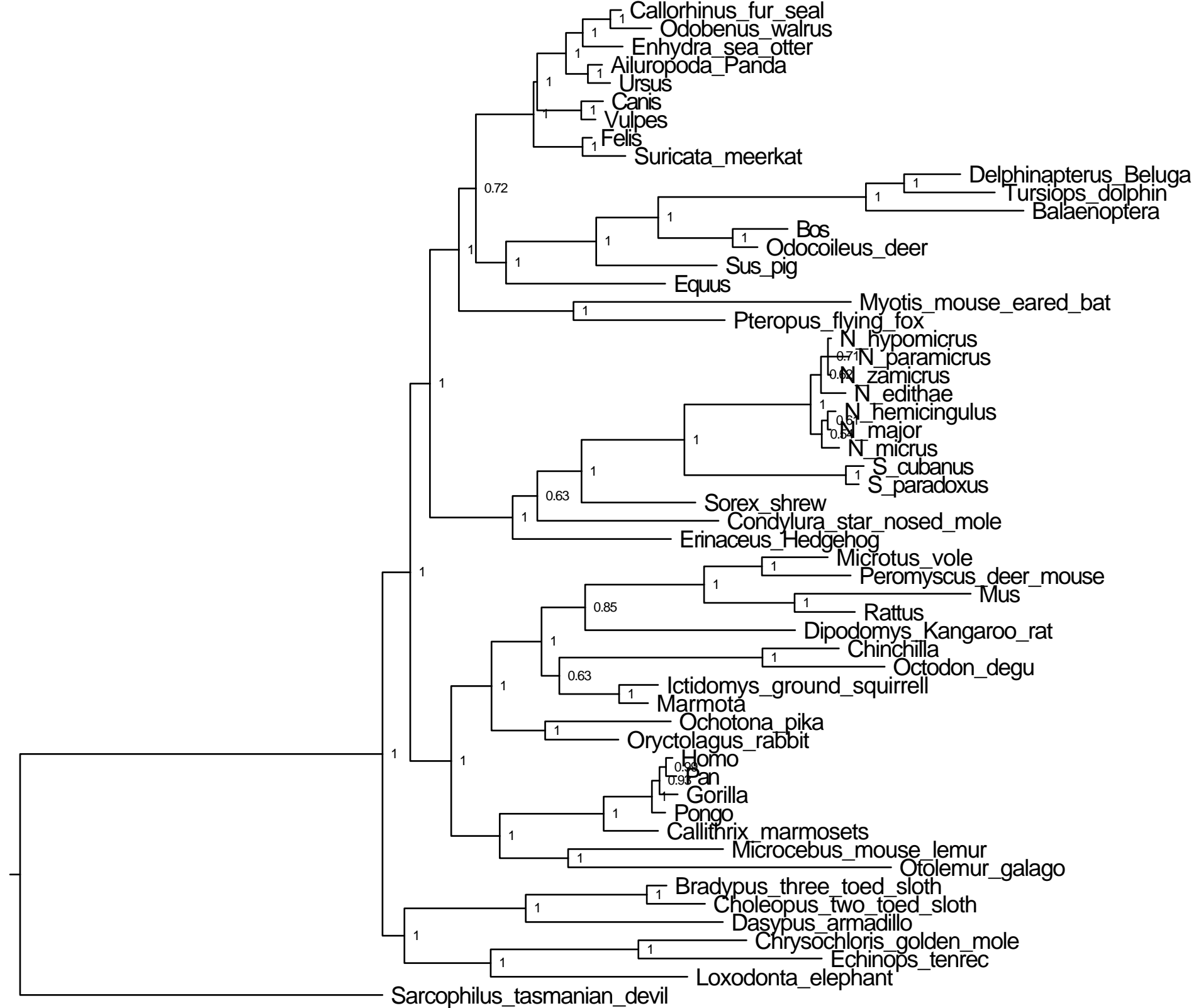

0.02

Supplement: msaa137_supplementary_data [file msaa137_supplementary_data.zip › fig_S6.pdf]

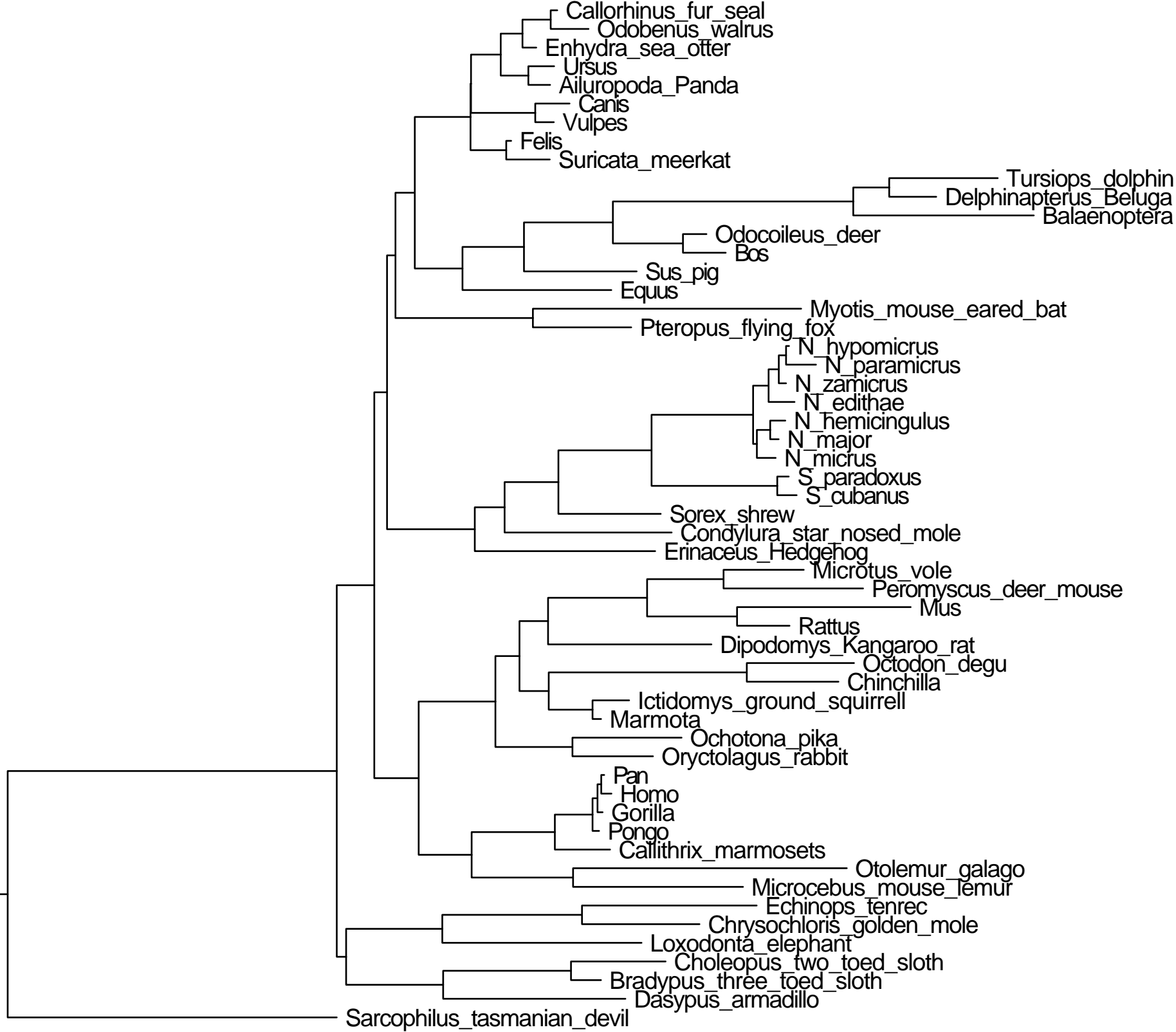

0.02

Supplement: msaa137_supplementary_data [file msaa137_supplementary_data.zip › fig_S7.pdf]

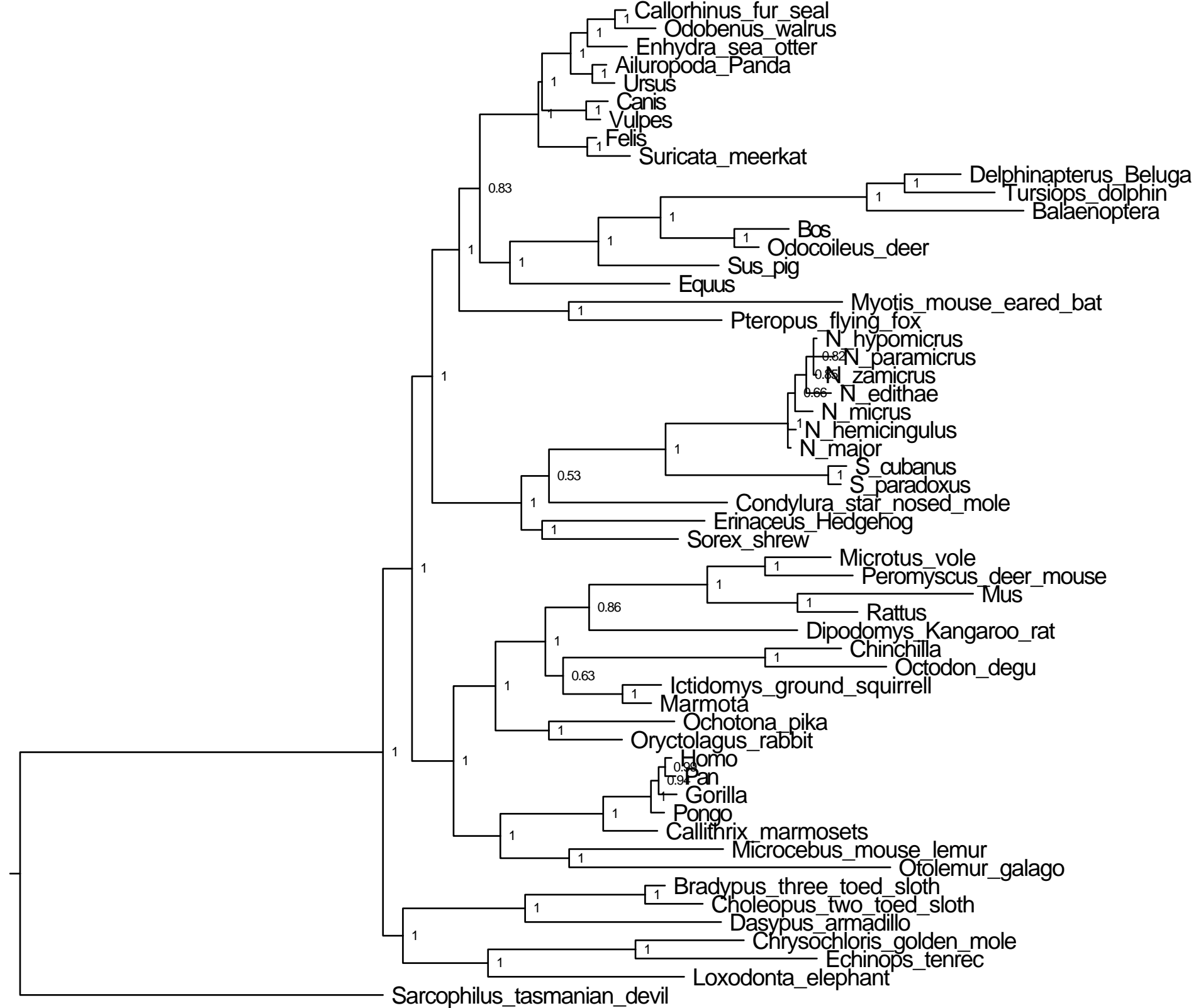

0.02

Supplement: msaa137_supplementary_data [file msaa137_supplementary_data.zip › fig_S8.pdf]

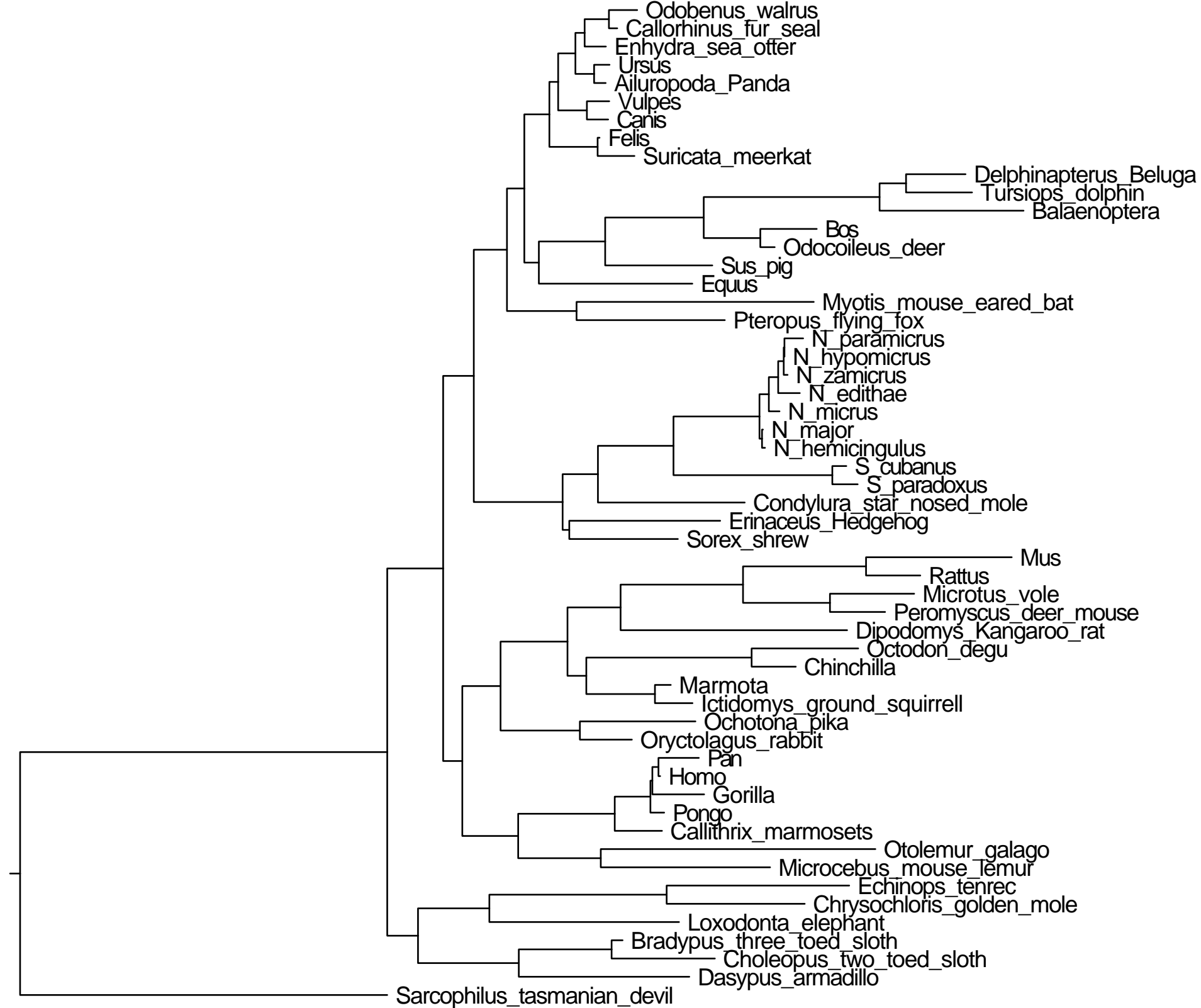

0.02

Supplement: msaa137_supplementary_data [file msaa137_supplementary_data.zip › fig_S9.pdf]
